# キリスト教系学校に在籍する生徒の一般的なイメージとキリスト教系学校の設立目的 - 東京・神奈川の若者を対象とした調査に基づく研究

**DOI:** 10.12688/f1000research.127483.2

**Published:** 2025-02-10

**Authors:** 後藤嘉宏 後藤, 片山ふみ 片山, 松井勇起 松井, 千錫烈 千, 照山絢子 照山, 大澤文人 大澤, 土屋深優 土屋, 横山幹子 横山

**Affiliations:** 1 Faculty of Library, Information and Media Science, University of Tsukuba, Tsukuba, Ibaraki, Japan; 2 Faculty of Literature, Seitoku University, Matsudo, Chiba, Japan; 3 Wako University, Tokyo, Japan; 4 Office of Institute Strategy, Institute of Science Tokyo, Tokyo, Japan; 5 Department of Sociology, College of Sociology, Kanto Gakuin University, Yokohama, Kanagawa, Japan; 6 Akikusa Gakuen Junior College, Tokorozawa, Saitama, Japan

**Keywords:** Christian schools, founding purpose, popular image of students, triple faith, mission

## Abstract

抄録

キリスト教主義学校が一定の人気を誇るにもかかわらず、日本のクリスチャンは日本の人口の1.6%しかいない。我々はこのギャップの原因を探ろうとしている。そこで2020年1月に東京都・神奈川県在住の15~29歳を対象にウェブモニター調査を行った。対象者にはキリスト教主義学校通学経験者、それ以外の関与者、非関与者を含み、キリスト教主義学校関与度による意識の相違を測ろうとした。そこで井上章一ほかが示した、トリプルな信仰という着想に焦点を当てた。仏教や神道は洗礼のような明確なプロセスを要さないので、双方をダブルに信仰する者も日本には多いが、キリスト教は洗礼を要する。洗礼を抜きに考えると多くの日本人は実質キリスト教も含めたトリプルな信仰をしている。その証拠がキリスト教主義学校の隆盛であり、クリスマスの受容であり、キリスト教式結婚式の人気であるという。この井上らの考え方は、日本でキリスト教は根づかなかったという通説と対立する。主要な設問とキリスト教主義学校関与度とのクロス集計結果を、インタビュー調査の結果で補うことで、基本的にキリスト教主義学校に通った者の方が、このようなトリプルな信仰に相当する意識を持っていることが分かった。またこのような結果となった理由についても併せて考察した。

## Abstract (en)

Despite the popularity of Christian schools in Japan, Christians account for only 1.6% of the nation’s population. In the attempt to explore this inconsistency, we conducted an online survey in January 2020 to those between the ages of 15 and 29 living in Tokyo and Kanagawa. Our subjects include those who have enrolled in Christian schools, those who have some sort of affiliation, and those who do not have affiliation. We attempted to measure the difference in attitude by the degree of their affiliation. We focused on the notion of triple faith which was proposed by Inoue and others. Many people in Japan believe in both Buddhism and Shintoism because neither requires a rite such as baptism. Christianity requires baptism, but that aside, in practice, many Japanese have triple faith including Christianity. Prosperity of Christian schools, tolerance towards Christmas, and popularity of Christian-style weddings all testify to this. This argument by Inoue and others conflicts with the common belief that Christianity never got a foothold in Japan. The results of cross tabulation of key questions and affiliation with Christian schools, combined with our interview results, show that those who have enrolled in Christian schools are more likely to have what may be referred to as triple faith. We have also provided a discussion on the possible reasons behind this finding.

## はじめに

キリスト教主義学校は、当事者も含めた一般の人びとから、どのような存在として受け取られて、イメージされているのであろうか。本稿はそのことをキリスト教主義学校の生徒・学生のイメージとキリスト教主義学校の設置目的の推測に焦点を当てて究明しようとした。

本稿の筆頭筆者が研究代表者を務める、科研費挑戦的萌芽研究「キリスト教主義学校から見る日本人の寛容と洋化－ステークホルダーらの期待と文化資本」では、キリスト教主義学校が日本の私立学校のなかで一定程度以上の定員比率を誇り、また人気を集める学校も多いにもかかわらず、日本のクリスチャンの数は日本の人口比で 0.8% しかいない（令和 5 年度版文化庁『宗教年鑑』）ことのギャップを探ろうとしている。そこで東京都・神奈川県在住の 15~29 歳を対象とした、ウェブモニター調査を行った。

本稿はまずはこの調査全体の背景や目的を述べるが、紙幅の関係もあり、今回の調査のうちキリスト教主義学校の設置目的を推測させる問いと、キリスト教主義学校生徒・学生のイメージについて聞く問いがあるが、それらを中心に分析し、考察する。

## 1. 先行研究と問題の所在

### 1.1 先行研究の概括的批評

本科研費研究の先行研究としては、國學院大學日本文化研究所教授（当時）で日本宗教学会長及び「宗教と社会」学会長を歴任した井上順孝を研究代表者にした科学研究費の研究、「現代日本における宗教教育の実証的研究」(1998~1999) がある。この研究は冊子体の報告書（井上順孝（研究代表者）科研費報告書『 「現代日本における宗教教育の実証的研究」(1998~1999) 報告書』(2000) 國學院大學）が刊行されている。この研究は宗教系及び非宗教系の学校全般を対象とし、この研究の報告書である 井上ほか (2000) には 10 編の報告論攷が載っているが、多くの論攷は非宗教系学校のみを対象としたり、宗教系及び非宗教系の学校全般を比較したりしている。また宗教系の学校についてもキリスト教主義学校に絞った研究ではない。しかしそれらのうち「宗教系大学別の分析」 （磯岡哲也）と「宗教系高等学校の入学者と卒業生の傾向についての一考察」（市川誠）は本研究の直接の先行研究といえる。これら二つの論攷は、宗教系の高校あるいは大学についてその学校の宗教ごとに比較しているので、キリスト教主義学校の特徴も分析されているからである。ただ基本的にこの報告書は、当該科研費助成期間以前の 1995 年から「宗教と社会」学会と國學院大學日本文化研究所で共同で毎年行っていた、質問紙調査の分析に充てていて、これらの調査は高等教育機関の学生の宗教意識を探る目的のものである。出身高校名や在籍高等教育機関の名前を自由記述式で聞く問いがあるので、それらを設置母体の宗派でカテゴライズしクロス集計することによって市川や磯岡の上記論攷は成り立っている。また國學院大學日本文化研究所編井上順孝責任編集『宗教と教育』(1997)も宗教系及び非宗教系の学校全般を対象としているが、カトリック及びプロテスタントそれぞれの明治期の宗教教育についての章があり、また質問紙調査については宗教系大学生の宗教意識、宗教系高校生の宗教意識の章はあるが、これもキリスト教主義学校に絞った調査及び分析ではない。

直接キリスト教主義学校に絞った研究は我々の科学研究費申請時には佐藤八寿子『ミッション・スクール―あこがれの園』 (2006) しか存在していなかった。この本は新書ではあるが竹内洋の指導の下に佐藤が京都大学大学院教育学研究科に提出した修士論文を加筆・修正したもので、キリスト教主義学校を見る周囲の視線に注目し、それが「運命の女性」という男性目線の理想の女性像に通じる点を明らかにした。またピエール・ブルデューの階層再生産の議論を援用しながら、良妻賢母を生むミッションスクールが卒業生の女性に、結婚を通じて良い社会階層を得る機会を創出してきたと論じた先駆的な研究である。しかしこの研究は小説、雑誌、漫画等の過去のメディア上のコンテンツの分析を主たる方法とするため、研究のバイアスとして佐藤自身が記しているように（ 佐藤 2006 p.89）、キリスト教主義学校の男子学生についての議論は充分でないし、実際に人びとのいだく意識等の調査はしていない。

そのあと我々の科研費助成期間初年度の 2018 年に、現在、日本文化研究センターの所長を務める井上章一が筆頭著者の『ミッションスクールになぜ美人が多いのか』（ 井上ほか　2018）が出た。これも新書であるが、4 章で構成される同書のうち、井上担当の「まえがき」と第 1 章「プロテスタント校はあなどれない」は一般受けを狙ったエッセー風評論であるが、井上の後輩同僚日本文化研究センター（当時）の郭南燕（現在、明治大学教授）による第 2 章「ミッション系大学の成功物語」、上智大の川村信三の第 3 章「変遷するキリスト教イメージ」第 4 章「 『お嬢様学校』を生み出したカトリック」を含めた全体は学術的であるし、井上のエッセー的な章へのデータの裏づけも含めて今回の調査票を設計した。

しかし佐藤同様、この本もキリスト教主義学校の女子のみに焦点が当てられ、キリスト教主義学校全般を知るには不足があるし、少なくとも第 1、2 章は小中高校ではなく大学に議論の焦点は絞られる。他方第 4 章に関してはタイトルにある「お嬢様学校」について、「 『お嬢様』とは幼稚園、小学校という低学年層から私立学校に通わせる裕福な家庭出身者ということになろうか」（ 井上ほか 2018 p.222）と記され、小学校への目配りもある。とはいえ第 3 章は学校に限らないキリスト教イメージ全般を対象としているので、本全体のトーンは第 1、2 章のキリスト教大学の女子が決めている。

しかし校種（あるいは課程）相互の定員の差から察せられるように、初等中等教育課程からの持ち上がりで高等教育課程に入った者の方が、少数精鋭でキリスト教主義学校の教育理念を身体感覚として身につけていることは、本稿では紙幅の関係であまり言及しないが本研究のインタビュー調査でも示されていることである。キリスト教主義に限らず、附属・系列学校等、大学より若い年齢の課程のある私立大学の場合、附属学校等の出身者の方がその学園独自のスクールカラーに染まって社会に旅立つ。他方ラサールや栄光学園のように東京大学合格者上位に名を連ねるカトリックの中高一貫男子校の多くは、大学附属ではない ^1^。したがって井上らの対象としているキリスト教主義学校の校種、生徒・学生のカテゴリーには偏りがある ^2^。

またこの井上らの本は第 2 章で統計資料、第3章、第4章で歴史的な文献資料を扱っているが、現在の人びとの意識についての実証に乏しい。「はじめに」や第1章で統計資料、文献資料以外で現代の人びとの意識を知る術は、ほぼ井上の非常勤先の学生との会話や、現代風俗研究会での報告会、ICU でのシンポジウム等で交わされた応答のみである。

しかし井上の現代の人びとに対する知見には説得力はあるので、それも調査票の設計では勘案した。

またやはり本研究採択年の 2018 年にクリーグ波奈の「キリスト教主義学校の役割とその教育的意義」という論文が東大の教育学研究科の紀要に載った。この研究はキリスト教主義学校での価値の社会化、すなわち生徒が学び身につける価値に着目し、キリスト教主義学校の A 校の社会化装置を文献資料から検証する（ クリーグ 2017）。この研究の存在自体、しばらく我々は見落としていたし、ここでレビューされる研究も見落としているものが多かったが、この研究もこの研究のレビューした研究も、キリスト教主義学校の教員や生徒等の当事者の認識のみに焦点が当てられている。他方、本調査は次項 1.2 で述べるように、当事者と非当事者を含む、普通の人びとのキリスト教主義学校への意識を問う中で、場合によって両者の比較をめざしている点で、クリーグ及びそこでレファーされる研究とは違う。

### 1.2 本研究の目的　

「はじめに」で本科学研究費の研究全体の目的を概ね記したが、改めてここで確認しておく。日本のクリスチャンの数は日本の人口比で 0.8% 前後しかいないにもかかわらず、キリスト教主義学校が日本の私立学校のなかで一定程度以上の定員比率を誇り、また人気を集める学校も多いのはなぜかを究明することが、本研究の目的である。この科研費研究はウェブモニター調査とインタビュー調査、各種資料の内容の分析（B.ベレルソンのいう狭義の内容分析法に限らず、定性分析も含む）の 3 本立てで、この研究目的を達成しようとした。ただ折からの新型コロナウイルスの影響もあり、インタビュー調査、資料の内容の分析は充分にできているとはいいがたい。

本稿は、この本科学研究費の研究全体のうち、ウェブモニター調査の結果の一部（ただし我々が最重要と思う部分）の報告に留める。

当然本項第一段落の「日本の人口比で 0.8% 前後しかいない」という事実は、やはり本項第一段落にある文から引けば「キリスト教主義学校が日本の私立学校のなかで一定程度以上の定員比率を誇り、また人気を集める学校も多い」という状況と矛盾しうる。なぜならキリスト教主義学校が布教、ないしは布教の地ならしをそこでの教育でしているとすればクリスチャンは日本の人口比の 0.8% を遥かに越えるはずであるからである。ではキリスト教主義学校は何を目的として設立されているのであろうか。この「キリスト教主義学校は何を目的として設立されているか」ということの解明が、《 「日本の人口比で 0.8% 前後しかいない」という事実が、「キリスト教主義学校が日本の私立学校のなかで一定程度以上の定員比率を誇り、また人気を集める学校も多い」という状況と矛盾する状況》の解明に役立つ作業仮説といえる（これを作業仮説 1 とする）。

もちろんこのことをキリスト教主義学校の経営陣に直接聞いたり、彼らの発言を文献で追ったりすることの方が、より直接的にこの問題の答えが出てくる。しかしそれだけだと人びとがどういう理由でキリスト教主義学校を現在、受け入れているかは見えてこない。キリスト教主義学校の強い当事者、弱い当事者、非当事者という層で人びとを分けて捉え、層ごとの意識の相違を探ることで、人びとがどういう理由でキリスト教主義学校を受け入れているかが朧気ながら浮かび上がる。

したがって、1.3 で後述する先行研究での議論とも関係してくるが、キリスト教主義学校の設置目的をどのように想像するかという問い (Q34) を聞き、その回答を、キリスト教主義学校の強い当事者、弱い当事者、非当事者という層で分けて捉えることで、《 「日本の人口比で 0.8% 前後しかいない」という事実が、「キリスト教主義学校が日本の私立学校のなかで一定程度以上の定員比率を誇り、また人気を集める学校も多い」という状況と矛盾する状況》の解明をしようと我々は考えた。このキリスト教主義学校の強い当事者、弱い当事者、非当事者という層で分けて捉えることは、1.3.1 や 2. で後述されるように、Q12 という変数を組変数にした Q12G によって行った。特にウェブモニター調査という性格上、キリスト教主義学校の通学経験者、通学経験者ならずとも家族に通学経験者がいるその他何らかの意味での当事者、双方の比率がウェブモニターの人びとに多い訳ではない。しかしある程度の比率、こういう当事者たちもモニターにいることが想定される。したがってキリスト教主義学校への強い当事者（通学経験者）、緩やかな当事者（家族に通学経験者がいる、受験したことがある、等）、非当事者、という三者での意識の差を探ることを、このウェブモニター調査の分析の軸にしようと考えた。

具体的には Q34 でキリスト教主義学校の設置目的をどのように想像するかという問いを設けた。そこの選択肢のうち、単純に考えれば、強い当事者、緩やかな当事者、非当事者の順に選ばれ、その選ばれ方の頻度の差に有意差のあるものは、設置目的に相当するものであると、当事者たちから捉えられている選択肢と考えられる。ただし実際には緩やかな当事者、強い当事者、非当事者の順に選ばれるもの等、色々なケースがありうる。

また《 「日本の人口比で 0.8% 前後しかいない」という事実は、「キリスト教主義学校が日本の私立学校のなかで一定程度以上の定員比率を誇り、また人気を集める学校も多い」という状況と矛盾する事態》であるが、その理由の解明にもう一つ役立つのは、キリスト教主義学校の生徒・学生イメージを、人びとがどのように捉えているかを見ることでもあり、それも作業仮説であると考えられる（これを作業仮説 2 とする）。

具体的には Q29 でキリスト教主義学校の生徒・学生のイメージを尋ねる問いを設けた。これも Q34 について述べたのと同様、そこの選択肢のうち、単純に考えれば、強い当事者、緩やかな当事者、非当事者の順に選ばれ、その結果に有意差のあるものは、キリスト教主義学校のイメージと、当事者から捉えられている選択肢と考えられる。ただし後述する結果の説明をやや先取りするが、こちらの問いの場合には Q34 の場合について以上に、実際には緩やかな当事者、強い当事者、非当事者の順に選ばれるものがあったので、その点についても結果の分析の箇所で考察を加えた。

なお、これら Q34、Q29 の選択肢として具体的に何を選ぶかについては、先行研究の議論を踏まえて我々は考えたので、次の 1.3 でその議論を見て、そのうえで作業仮説について、もう少し言及する。

### 1.3 先行研究と本稿でとりあげる、本研究の調査票設計との具体的な対応関係

1.3.1 概況　

前項でとりあげた 井上ほか (2018) の内容の概略を、本稿に関連する限りで、以下に5つ列挙する。①日本にキリスト教徒は少ないが、日本人はダブルに信仰する民族であり、キリスト教に洗礼という手続きが不要であればキリスト教を含めトリプルな信仰をする人は多かったはずである。なお、このダブル、トリプルの問題は、本稿の分析・考察の軸になるものであるので、その詳細は 1.3.2 で説明し、また調査結果としては 3.3 で述べる。②その証拠はキリスト教式結婚式及びそれと類似したキリスト教主義学校の隆盛である。③女子アナウンサーにキリスト教主義学校出身率が高いのは、愛と奉仕の精神を育むとそれらの学校のウェブページに記載されていることに照応する。④皇族女子のキリスト教主義学校入学が多いのも、愛と奉仕の精神を育むとの期待があるからである。⑤キリスト教主義大学女子はきれい、金持ち、キリストの 3K といわれる。

さらに「現代日本における宗教教育の実証的研究」 (1998~1999) のなかで指摘された、本研究に密接にかかわるものとして以下の⑥を挙げる。⑥キリスト教主義に限定した話ではないが、宗教系の学校の設立目的には、その宗派の信徒の子弟の教育機会の確保と、その宗派のシンパを作ることにある ^3^。

1.3.2 以下で必要に応じてこれらの補足説明を加えつつ、本稿にほぼ関わる限りでの本調査の設計に言及する。

なお、今回の調査では 1.2 ですでに若干ふれ、また後で 2. で詳述するように、Q12 で「あなたのキリスト教主義学校への関与についてお聞きします。当てはまるものを選んでください」という設問を設けた。2. で後述するようにこの Q12 の回答を 3 つのカテゴリーに再グループ化した Q12G という変数を、他の多くの変数を分析する際の軸として考えて分析を進める。

1.3.2 首尾一貫性かトリプルな信仰か、およびキリスト教式結婚式、等

まず、1.3.1 の①についてであるが、郭は「宗派に帰属しない日本人のトリプルな信仰」（ 井上ほか 2018 p.136）という見出しの下、以下のように論じる。神道、仏教あわせた信者数は 1 億 7244 万人で日本の総人口より 5000 万人多い。これはダブルな信者が多いためであるが、入信等の儀式はほとんど受けてはない。他方、キリスト教では入信に洗礼を要するという知識を多くの日本人は得ていて、この人数の膨れ上がりにキリスト教は加担しない。しかし、狭義に入信したとはいわれないが、日本人の意識としては仏教、神道のダブルな信仰に加えてキリスト教も加えた「トリプルな信仰」という意識をもつのではないか、と郭はいう。その証拠がキリスト教式結婚式の隆盛とキリスト教主義学校の人気、そして国民的行事と化したクリスマスであるという。

当然この郭の議論への反論として、クリスマスは言うに及ばず、キリスト教式結婚式も一つの風俗にすぎないとの見方もありうる。しかしながら濱田陽によるこの領域の研究（ 濱田 2001 pp.23−46）を読むと、郭の議論をある意味で裏づける事実に遭遇する。濱田のいうには、キリスト教式結婚式は、戦後間もなくYMCA が会員男性から非クリスチャンの一般信徒へとキリスト教式結婚式の門戸を開いたことが端緒であるが、これはその当時としては例外で（ 濱田 2001 p.25）、1960 年代末に、高度経済成長を背景に青学会館、東京 YMCA ホテル、等が一般人向けに開放され、またキリスト教徒も所属教会での挙式が手狭である場合には、これらを選択したことが事実上の始まりという。もっとも今日隆盛しているホテル等でのキリスト教式結婚式は、1980 年キリスト教ブライダル宣教団の牧師たちが日本人の若年層がキリスト教にふれるきっかけとして始めた（ 濱田 2001 p.33）。またカトリックについて濱田は日本のカトリック中央協議会が1973年にローマ教皇庁に提出した文書を紹介する。この文書では、キリスト教式結婚式を望む非キリスト教徒について、神社仏閣でなくあえてカトリックを選ぶことは彼らがカトリックを評価している証であると記し、またそういう申し出をする者の多くがカトリック系の学校の卒業生であると述べている点も濱田は指摘する（ 濱田 2001 p.29）。要するにキリスト教式結婚式は風俗の面も強いが、プロテスタント、カトリック共に聖職者の一部がそれを信仰の一歩手前のものとして肯定的に評価する動きもあり、またキリスト教主義学校との関連性も少なくともカトリックの聖職者たちからは意識されていることが、この濱田の研究で示される。もっともその一方でカトリックに関しては、日本側は宣教手段として結婚式を位置づけるべくお伺いを立てたが、バチカン当局は宣教ではなく霊的指導に留めるようにと指導し、他方、指導の後も日本のカトリックの側は霊的指導と共に宣教的方針を文書で明記しているという食い違いがあるとのことである（ 濱田 2001 p.30）。

このキリスト教式結婚式については本調査では Q22 で「Q22. キリスト教徒ではない日本人のカップルが、教会で結婚式を挙げることについて、あなたはどのように思いますか」という設問で 3 選択肢 1 択の SA で聞いている。

またトリプルな信仰という郭の指摘に対しては、「あなたが思う、キリスト教主義学校に対する設置母体のメリットに当てはまるものを上位 3 つまでの間で選び、中でも最もメリットだと思うものを 1 つ選んでください」とキリスト教主義学校の設立目的を聞くQ34 における、選択肢 9「信者にならなくても、キリスト教の教えを守ることによって、救われる可能性のある人を増やすこと」や、入信していない場合の救済やご利益の有無について尋ねる Q49 や Q50 を聞くことで、妥当性の有無が分かる。これらの質問と 1.3.1 の最後のパラグラフで記した Q12 とのクロス集計の結果に有意差があれば、郭のいうトリプルな信仰の存在の予測の側面から、キリスト教式結婚式を捉える可能性が拓けるといえるし、有意差がなければその可能性は低く、それは習俗の面に過ぎないといえよう。しかしいずれにせよキリスト教主義学校やキリスト教式結婚式についてはそれがトリプルな信仰の一環、つまり洗礼なき信仰心がありうるという予測に通じる面がある可能性はあるが、国民行事と化したクリスマスについては日本人の非信徒の場合、イエス・キリストとはほぼ縁もゆかりもないものと化しているというのが、多くの日本人あるいは日本に永く住んでいる外国人の偽らざる実感であろう ^4^。とはいえ、この Q34 選択肢 9「信者にならなくても、キリスト教の教えを守ることによって、救われる可能性のある人を増やすこと」と キリスト教主義学校関与度を示す Q12 に関連があればトリプルな信仰そのものの可能性は拓ける。

また同書は「まえがき」でつぎのように記す。「われわれは素行の悪いクリスチャンを、よくなじる。・・・しかし浄土真宗や日蓮宗の人びとが浮気をしても、宗教にからめて批判しない。・・・つまり、日本ではキリスト教の方が良い宗教であるかのように、思われている。・・・江戸期には、淫祠邪教だとみなされた。そんなキリスト教が百数十年の時を経て、倫理面では信頼される宗教になりおおせている。そのサクセスストーリーが、これまでのキリスト教受容史では、見すごされている。講壇学説の一般通念には、その意味でこまったかたよりがある」（ 井上ほか 2018 pp.10-11〔・・・は中略。特記しない限り、以下同様〕）。

ここで批判される「講壇学説」の内容を井上は次のように纏める。「近代の日本社会は、けっきょくキリスト教をうけいれなかったと、よく言われる。信者の人口は、日本人ぜんたいの二パーセントにも満たない」（ 井上ほか 2018 p.9）。「また、日本社会は、キリスト教を歪曲してうけとめたとも、評されやすい」 （ 井上ほか 2018 p.9）。

この「はじめに」の文言を、先にあげた同じ著書の郭の先述の主張に照らしていい直してみると、次の 《 》に入れた文章のようになろう。《キリスト教は日本社会に定着せず敗者のようにいわれるが、キリスト教式結婚式、キリスト教主義学校、クリスマスの隆盛等に示される、仏教徒でありつつ神道の信者でもありキリスト教徒でもほぼあるという、トリプルな信仰の表れの状況を踏まえると、日本においてキリスト教を敗者とみなす見方は誤りである》。

井上章一自身は第 2 章末尾で、先行研究の 佐藤 (2006) の日本でのキリスト教主義学校のもたらしたものは文化でありふるまいであったという発言を引きつつ、次のようにいう。「私は「ふるまい」もさることながら、キリスト教の倫理観に共鳴している日本人が数多くいると思っている。宗教や宗派にこそ帰属しないものの、仏陀の悟り、神道の霊魂とともに、キリスト教の神をも尊敬している」（ 井上ほか 2018 p.160）。このように佐藤の「ふるまい」としてのキリスト教の考え方を表向き肯定しつつも、それについて「さることながら」と述べて、「ふるまい」、習俗としての受容以上に倫理観としての受容があると論じ、その倫理観を「仏陀の悟り」「神道の霊魂」「キリスト教の神」という信仰の中核の言葉に置き換える。一見すると、井上に要約された「限りでの」佐藤の著書の見解を肯定するように書きつつも、信仰から切り離された習俗・「ふるまい」としてのキリスト教受容という見方を否定しようとしている。井上は参照頁を明示せず内容に言及するに留まるので、佐藤自身のテキストのどこが井上による言及の該当箇所なのか想像するしかないが、佐藤が佐藤卓己 ^5^と共訳したモッセに依拠しつつ「TPO に応じたリスペクタブルなふるまい」（ 佐藤 2006 p.25）と書かれた箇所のことだと推察される。仮にそこだとすると、ウェーバーの ethos、ブルデューの habitus 由来の、形が本質を決めることにも通じる考え方が強調されるが、ここで信仰と切り離した捉え方を佐藤が標榜している訳ではない。日本でのキリスト教受容は習俗であるとは述べるが、習俗が信仰を決定づける可能性は排除されない。「キリスト教に根ざした教育とは、すなわちリスペクタビリティの教育でもあった。洋風生活におけるマナー、作法といった、些末な行為の数々は、単に些末な行為の数々であるだけではない。神への敬虔は、現実の中では「つつしみ」がある生活態度として表出されねばならなかったのだ」（ 佐藤 2006 p.25）。佐藤自身はこのように型と精神の相互作用を想定する。

その点で井上の佐藤への婉曲な批判は厳密にはミス・リーディングといえる。ただし結果的に両者とも、日本人のキリスト教受容に対して、それらは一見ふるまい、形としてのみの受容であるが、その根底に倫理や精神性、さらに信仰に通じるものを孕むと考える点では、むしろ共通する。

ここで井上章一らは丸山真男の名前を出していないし、彼を多少なりとも意識しているか否かも定かではない。しかしそもそもトリプルな信仰は、丸山の描くキリスト教の日本における受容の姿と真逆に相当する。というのも、首尾一貫性を重んじるものであるがゆえ、キリスト教は日本人あるいは日本文化の無限抱擁性と相容れず排除され、定着しなかったというのが、丸山の主張の骨子であるからである（ 丸山 (1961) の言い方は「マルクス主義やキリスト教」という言い方であって、キリスト教はややマルクス主義の添え物ではあるが）。

丸山『日本の思想』は日本の固有信仰に由来する日本人の心性として無限抱擁性（ 丸山 1961 p.14）と中心性の欠如を指摘する。これは丸山の言葉ではないが、神道には、祝詞はあるし、経典として古事記、日本書紀、風土記等が列挙されるが、神道は明確な正典を欠く。伊勢神宮の簾の向こうは、初代文部大臣で明六社会長の森有礼が参拝のおり、杖で簾を持ち上げ示してみせ、彼の暗殺の一因になったとの説もあるように、不在で、中心がない。神道に典型的に示される日本の固有信仰の影響を受けた日本人は、神道に中心がないだけに、何でも受け入れる傾向にある。本地垂迹説や反本地垂迹説があるように、多様な宗教を受け入れる。別の著書である加藤周一・木下順二・丸山真男・武田清子『日本文化の隠れた形』（岩波書店）のなかで次のように丸山はいう。「神道というのは、はじめは仏教と習合して両部神道のような教義が生まれ、後には儒教と習合して、吉田神道とか吉川神道とかが出て来る。神道は、そういう他の世界観の助けを借りない と「教義」としての体系を持てないのです」（ 加藤ほか 2004 p.141）。以下の例も丸山にある訳でないが、洋食和食中華のいずれも好んで食べ ^6^、年末年始の一週間はクリスマスを祝い除夜の鐘を衝き初詣に参拝し初日の出を拝むことを同じ人がやっても違和感なく受け入れられる等、文化の雑食性、「無限抱擁」性が日本人にはある。よくいえば、日本の文化は思想的宗教的に寛容な文化であると解釈することが可能である ^7^。しかしそのような無限抱擁的な日本人の主に庶民が不寛容になる対象がある。丸山のいうにはそれが一神教のキリスト教であり、マルクス主義であるという（ 丸山 1961 p.15）。この言葉も丸山の言としては確認していないが、双方とも『聖書』『資本論』という正典とでも称すべき絶対的典拠がある。丸山の言葉を使えば、両者とも座標軸をもって物事を見ていく （ 丸山 1961 p.5）。他方、日本の固有信仰、神道は中心が欠如する（ 丸山 1961 p.21）がゆえに「寛容」であるのであるのだから、逆にそういった日本の宗教をはじめとする思想・文化は、正典という中心から首尾一貫性をもって世界を眺め解釈し正邪を判断するような一神教的な宗教や思想とは、まったく相容れないことになる。そういう首尾一貫性、いいかえるとある種の不寛容性をもつキリスト教、マルクス主義に対して、無限抱擁的な雑食文化のなかにいる日本人、とくに日本の庶民は、不寛容性を示す。さらに庶民がそうであるのと同時に、一人の人間の中に二つの傾向が併存しうるので、その点では知識人にも同様の側面がある（ 丸山 1961 p.52）と丸山はいう。

このような丸山の趣旨は《不寛容なものに対する不寛容》性を強く有する無限抱擁性（寛容性）と要約できようが、その議論からすると、ダブルな信仰は日本人の無限抱擁性に通じる。他方、郭はダブルとトリプルを連続して捉えるが、キリスト教をその一つに含めたトリプルな信仰という、郭を含めた 井上ほか (2018) の着想は、丸山の文脈では、首尾一貫性を重視するキリスト教という位置づけから考えてあり得ないことになる。丸山からするとダブルと（キリスト教をその一つに含めた限りでの）トリプルとの間には断絶があるが、 井上ほか (2018) では、これらは連続する。ただしキリスト教の宗派や学校の側、クリスチャン自身の側がどう考えているかは今回の調査対象者の基本属性からひとまず措いて、日本の一般の人びとの対キリスト教意識を今回は測定し議論する。よって、丸山の時代とは大きく変わっている可能性もある。そしてそもそも、現代の人びとは首尾一貫した体系を有する宗教としてキリスト教を眺めない可能性がある。

丸山を法学部助手に抜擢した南原繁は宗教的には内村鑑三の弟子で新渡戸稲造の影響も強く受けた。一方、唯物論研究会の発起人会議長も務めた長谷川如是閑が父親の親友で彼に幼少期より可愛がられ、第一高等学校在学中に如是閑の出る唯研の講演会を聞きに行ったがため警察に拘留された経験もある丸山に、当然クリスチャン歴はない。もっとも 遠藤 (2016) では、丸山のキリスト教からの影響を次のように記す。 「南原、三谷は内村鑑三から薫陶を受けた無教会キリスト者であり、丸山は両者から高校時代には法律哲学(法制)を、大学に入ってからは政治哲学を学ぶ経験は持ったものの、その後、キリスト教を介して、同信となることはなかった。しかし、丸山にとってこのドストエフスキーは、一方で自らの社会主義観とも深くつながっており、その意味で重要な作家であったが、他方キリスト教の理解という点でも、深く実存的な影響を受ける作家となった」。したがって、内村、南原を通じてキリスト理解の下地を得て、さらにドストエフスキーを通じて丸山のキリスト教観は形成されているといえる。いわば丸山は「 『イエスを信じる者』の契約」を誓った（ 内村 1958 p.28）札幌農学校二期生（新渡戸、内村も二期生）の孫弟子にあたる点は、彼の、首尾一貫性を求めるキリスト教像の背景として考えられる。また丸山には、長い禁教の時代を経た、明治期というキリスト教の事実上の復活期から数えると、まだキリスト教が事実上「新宗教」であった時代の息吹を、南原から伝え聞く機会は何度もあったはずである ^8^。したがって丸山から幾世代も隔たった、令和のいわゆる既成キリスト教は日本においても既成宗教と化しており、その令和のさらに若年層に聞いた今回の調査結果は、丸山のイメージするキリスト教と異なる可能性も否定できない。実際、クリスマス、キリスト教式結婚式、キリスト教主義学校は、習俗としてであれ倫理の表れとしてであれ信仰の入り口としてであれ、排他的ではないキリスト教というイメージを日本人の間に醸成するのに貢献した可能性のあることは否めない。

その意味も籠めて今回の調査票を設計した。トリプルな信仰についての設問は先述の通りだが、それ以外にも首尾一貫したものへの寛容性についての設問もいくつか設けたが、そちらについては紙幅の関係もあり、別稿で論じる。

1.3.3 愛と奉仕の精神、および一部女性皇族のキリスト教主義学校への入学について

郭は女子アナウンサー出身校上位を占めるキリスト教主義大学の建学の精神を探るため、これらの大学のウェブサイトを探ると、いずれもそこで愛と奉仕の精神を謳っていると指摘する（ 井上ほか 2018 p.98）。しかし建学の精神がそのまま在校生や卒業生に浸透していると考えるのは、教育効果を楽観視し過ぎている。もっとも一般人ではなく、愛と奉仕の精神をご公務で日々示される皇族方にあらせられてはその限りではなく、皇族女子がキリスト教主義学校にご入学する、あるいはキリスト教主義学校出身者を男子皇族の配偶者にお迎えすることは、実際に行われてもきた。

キリスト教主義学校の生徒・学生・卒業生の愛と奉仕の精神については、調査票の Q29 でキリスト教主義学校の生徒・学生のイメージについて聞いた選択肢のなかに含めている。Q29 の選択肢 4「他人を思いやる心が豊かである」、選択肢 7「社会的活動への関心が高い」でこれらについて測定しようとしている。

また女性皇族については、Q11 で「女性皇族にキリスト主義学校出身者が複数人いることについて、あなたはどのように評価しますか」と 5 者 1 択の SA で聞いている。ただし調査実施時期の 2020 年 1 月の時点で ICU 出身の秋篠宮眞子内親王（当時；現、小室真子氏）の婚約並びに結婚延期の報道がなされていたため、回答にそのことによるバイアスがあった可能性はある。

1.3.4 きれい、金持ち、キリストの「3K」

井上は上記の著書の「はじめに」でキリスト教主義大学の女子を「きれい、金持ち、キリストの 3K」であるという噂についてその信憑性を検証しようとしている。非常勤先の仏教系女子大生が、自己卑下するなかでキリスト教主義女子大生をこう評して井上に語ったことが井上の着想の契機である。「検証」といっても先にもふれたが、非常勤先や会合等での私的な会話のなかでその噂が確からしいことを確認しているにすぎない。

とはいえキリスト教主義大学の女子のメディアへの登場頻度を独自に数え上げる等、マス・コミュニケーション学の内容分析的な作業をした資料は井上も作成し載せている。例えば 2005 年から 2014 年の「女性ファッション誌への学生モデル登場率の推移」という表（ 井上ほか 2018 p.74）では、読者モデル起用率で上位にキリスト教主義大学の女子学生が位置づけられていることを、数字をあげて説明している。

しかし、ファッション誌編集者の眼にキリスト教主義大学の女子学生が 3K ^9^ であると映っていることは、この表から証明されるが、実際に編集者以外の、ふつうの市井の人びとが 3K の意識でキリスト教主義大学女子を捉えているか否かはこれだけでは分からない。

本調査ではこれもキリスト教主義学校の生徒・学生・卒業生のイメージを聞く、Q29 の選択肢に、この 「きれい」「金持ち」のイメージの有無を測定するものとして、「裕福である」「清貧である」と「容姿端麗である（みめうるわしい） 」「容姿端麗ではない（みめうるわしくない） 」を加えた。ただし 佐藤 (2006) や 井上ほか (2018) と異なり、現代は女性に対するジェンダーバイアス（以下、女性バイアスと略す）を当然視できない時代ゆえ、生徒・学生等の性別を問わない表現にした。

1.3.5 キリスト教主義学校の設立目的について

「現代日本における宗教教育の実証的研究」(1998~1999) では、宗教系の学校全般の特徴として、信徒の子弟の教育機会の確保とその宗派へのシンパの獲得が、設立目的の例として示されていた ^10^。このことはキリスト教主義学校に限定してもそのまま妥当するのかどうかを検証する必要がある。もっともこの研究ではキリスト教主義学校については、同志社の 1930 年頃までの卒業生の例を示して、現在とは比べ物にならない入信率を誇っていた時期のあることも示してもいる。この点については、後述するように 佐藤 (2006 p.17) でもミッション系高等女学校についてのみだが、表に示している。また「現代日本における宗教教育の実証的研究」では教育基本法並びに学校教育法が国公立学校に対してであるが、戦前の国家神道への反省から特定宗教の宗教教育に否定的であることも、宗教系学校が特定の宗教への布教に結びつくような宗教教育に及び腰な理由として挙げられていた ^11^。

もっとも教育基本法の制定を推進したリーダーは丸山真男の恩師南原繁で、南原は官立第一高等学校在籍時に校長新渡戸稲造に感化され、同高元教員内村鑑三に弟子入りし、その新渡戸と内村は官立札幌農学校二期生で、内村のいうには自身及び新渡戸ら二期生は、そのほとんどが入信した一期生の先輩たちに「 『イエスを信じる者』の契約」をせよと、クリスチャンになることを迫られたとのことである。「カレッジの世論はあまりにつよく余に反対であった、それに対抗することは余の力におよばなかった。彼らは左に掲げる契約に署名するよう余に強制した、・・・余はついに屈した、そしてそれに署名した」（ 内村 1958 p.22）。

したがって本研究では、信者の獲得という先行研究では一応否定されている選択肢も含めて、キリスト教主義学校の設立目的を幅広い選択肢で想定した Q34 を設けた。ここでは「あなたが思う、キリスト教主義学校に対する設置母体のメリットに当てはまるものを上位 3 つまでの間で選び、中でも最もメリットだと思うものを1つ選んでください」という MA、SA 複合の設問をし、選択肢は「その他」「答えたくない」「よく分からない」とそれら以外に 12 個用意した。選択肢の 1 は「信者の子弟の教育要求への対応」、選択肢の 7 は「キリスト教への共感者（シンパサイザー）を増やすこと」である。他に選択肢の 8 では「キリスト教の信者を増やすこと」を入れ、シンパを作ることより直接的な目的があると考えるか否かも問うた。他方、例えば選択肢 9 は「信者にならなくても、キリスト教の教えを守ることによって、救われる可能性のある人を増やすこと」とした。これはある意味で「キリスト教への共感者（シンパサイザー）を増やすこと」にも近い内容であるが、それと同時に 1.2 の①や 1.2.2 で言及した、首尾一貫した信仰かトリプルな信仰かということに密接にかかわる選択肢として用意した。

この設問のように MA と SA 双方での答えを求める設問を本調査では多く用意したが、これらは MA ベースで回答者の比較的多い選択肢については、その選択肢を選んだ人を 1、選ばなかった人を 0 とした二択の変数を作り、他の多くの質問群とクロス集計させうる。このようにしてこの調査の軸となる変数を設定することも意図した。　

### 1.4 概念図と分析の軸―また改めて作業仮説の提示

本稿のもとになったウェブモニター調査全体の概念図（ 図1）は以下の通りである。この図に記されている番号は我々の調査の問の番号で、調査票は https://doi.org/10.6084/m9.figshare.21367632.v1 ( Goto, 2022c) にて閲覧でき、そこに調査のローデータも置いてある。

**図1.**
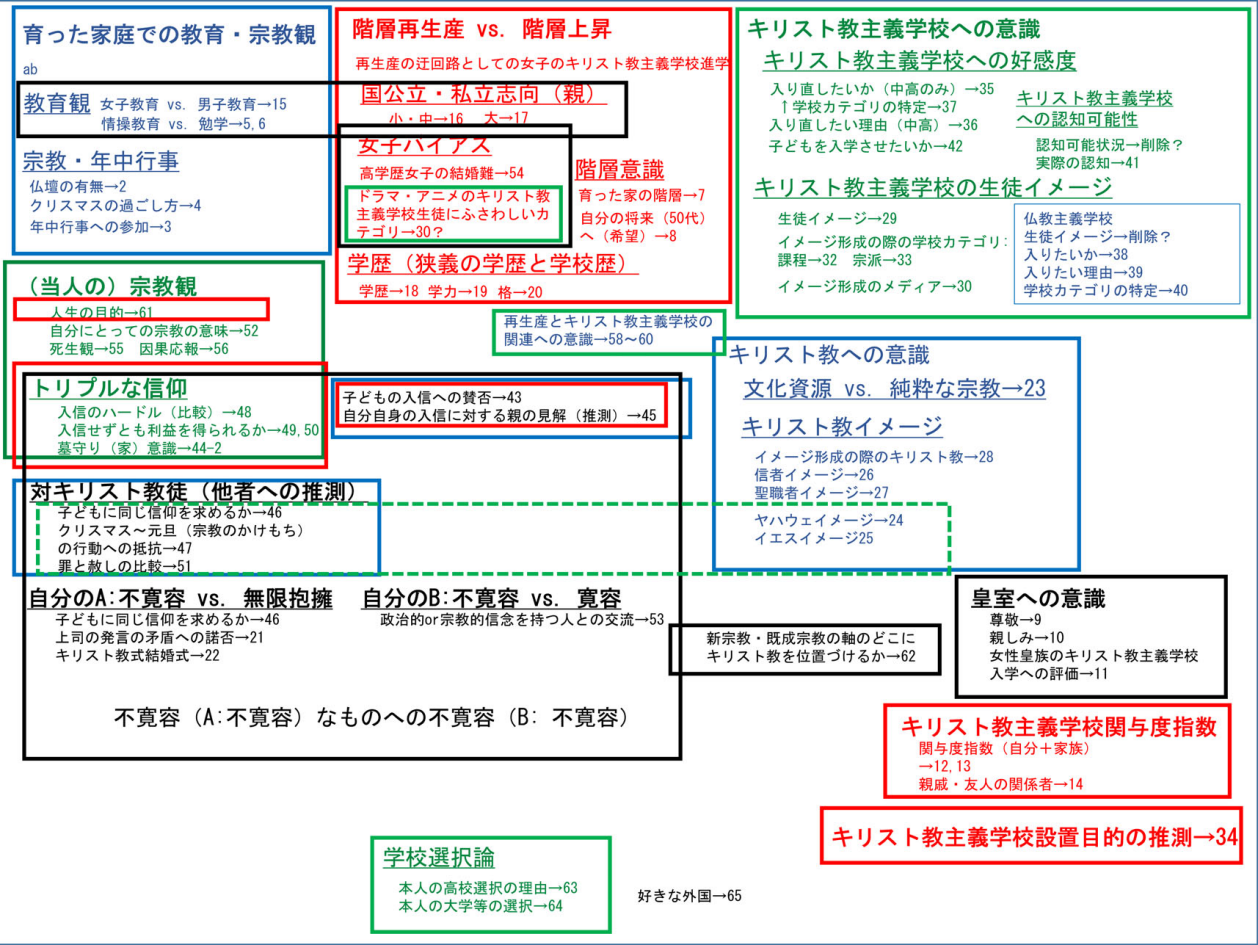
ウェブモニター調査全体の概念図.

このうち今回の報告（本稿）では、分析の一番の軸としてキリスト教主義学校への関与を示す Q12 を想定し、分析の二番目の軸に女性皇族のキリスト教主義学校への関与の是非を問う Q11 を想定する。

これらを主な独立変数としながらキリスト教主義学校の設立目的を推測させる Q34 並びにキリスト教主義学校の児童・生徒・学生イメージについての Q29 の各選択肢を主な従属変数として分析していく。

「1.2 本研究の目的」で本研究の目的とそれと係わらせてウェブモニター調査での作業仮説を示し、1.3 で本稿の調査票設計を先行研究との拘りで示してきたので、ここではそのことを受けて、改めて本稿での作業仮説を示す。

1.2 でみたように、《 「日本の人口比で 0.8% 前後しかいない」という事実は、「キリスト教主義学校が日本の私立学校のなかで一定程度以上の定員比率を誇り、また人気を集める学校も多い」という状況と矛盾する状況》であるが、その理由の解明に役立つのは、「Q34 でキリスト教主義学校の設置目的をどのように想像するかという問い」と考えられる。よってその問いを設け、その結果をみることを考えた。特に「その Q34 の各選択肢のうち、強い当事者、緩やかな当事者、非当事者の順に選ばれ、その選ばれ方の頻度の差に有意差のあるものは、設置目的に相当するものであると、当事者たちから捉えられている選択肢と考えられる」と1.2で述べた。この 1.2 での記述では、Q12 あるいは Q12 をグループ化した Q12G を独立変数として、Q34 の各選択肢を選んだ者、選ばなかった者を 1、0 とする従属変数とをクロス集計することが想定されている。

一方、1.3 でみた限りで、キリスト教主義学校と皇室女子との関係が、一般の人びとの、キリスト教主義学校のイメージ向上に関与しうる可能性が示唆された。したがって上述のように Q11 も独立変数として、Q34 の各選択肢を選んだ者、選ばなかった者を 1、0 とする従属変数とをクロス集計することをここで想定した。つまりキリスト教主義学校と皇室女子との関係を肯定的に捉える者は、キリスト教主義学校への通学経験等のキリスト教主義学校関与度とはある程度別の次元で（もちろん双方が完全に独立しているという訳ではない）、キリスト教主義学校に肯定的な可能性の高い人々と想定できる。したがってそういう人たちとそうでない人を Q11 でみた結果を独立変数として、Q34 の各選択肢を選ぶ選ばないを従属変数としてクロス集計でみていくことで、一般の人々のなかでのいわゆるファン層が、キリスト教主義学校設立目的をどう捉えているかが分かる（作業仮説1の具体的な形）。このように Q11 × Q34 の各選択肢を見ることで、Q12G × Q34 の各選択肢のクロス集計で得られた知見を補足することが可能となる。

また、これも 1.2 でみたように、《 「日本の人口比で 0.8% 前後しかいない」という事実は、「キリスト教主義学校が日本の私立学校のなかで一定程度以上の定員比率を誇り、また人気を集める学校も多い」という状況と矛盾する状況》であるが、その理由の解明にもう一つ役立つのは、キリスト教主義学校の生徒・学生イメージを、人びとがどのように捉えているかを見ることでもあり、それも作業仮説であると 1.2 で考えた。そこで具体的には Q29 でキリスト教主義学校の生徒・学生のイメージを尋ねる問いを設けた。これも Q34 について述べたのと同様、Q12G とクロス集計し、Q34 の選択肢のうち、単純に考えれば、強い当事者、緩やかな当事者、非当事者の順に選ばれ、その結果に有意差のあるものは、キリスト教主義学校のイメージと、当事者から捉えられている選択肢と考えられる。

そしてこの Q29 についても、1.3 での検討結果を経て、上述の Q34 の場合と同様、Q11 の皇室女子のキリスト教主義学校への関与の是非の問いとのクロス集計も有効であると考えられる。要するにこれも上述のQ34 の場合とほぼ同様の記述をくり返すが、キリスト教主義学校と皇室女子との関係を肯定的に捉える者は、キリスト教主義学校への通学経験等のキリスト教主義学校関与度とはある程度別の次元でキリスト教主義学校に肯定的な可能性の高い人々である。したがって Q11 の回答で肯定的な人たちとそうでない人とを Q11 で独立変数として、Q29 の各選択肢を選ぶ選ばないをクロス集計でみていくことで、一般の人々のなかでのいわゆるファン層が、キリスト教主義学校の学生・生徒イメージをどう捉えているかが分かる。つまり Q11 × Q29 の各選択肢を見ることで、Q12G × Q29の各選択肢のクロス集計で得られた知見を補足することが可能となる（作業仮説2の具体的な形）。

1.3 で示された、先行研究の最重要な知見は、トリプルな信仰があるか否かである。本科研費研究全般の目的、「日本のクリスチャンの数は日本の人口比で 0.8% 前後しかいないにもかかわらず、キリスト教主義学校が日本の私立学校のなかで一定程度以上の定員比率を誇り、また人気を集める学校も多いのはなぜかを究明すること」と、この「トリプルな信仰」の有無を照らしあわせてみる。

いままで Q34 や Q29 での議論は、「 「日本のクリスチャンの数は日本の人口比で 0.8% 前後しかいないにもかかわらず、キリスト教主義学校が日本の私立学校のなかで一定程度以上の定員比率を誇り、また人気を集める学校も多いのはなぜかを究明すること」という命題の後半部分、「キリスト教主義学校が日本の私立学校のなかで一定程度以上の定員比率を誇り、また人気を集める学校も多いのはなぜかを究明すること」にのみ焦点を当てていたといえる。他方、もしもトリプルな信仰が成立するとすれば、「日本のクリスチャンの数は日本の人口比で 0.8% 前後しかいないにもかかわらず」という部分を吟味することに なる。

つまり、 井上ほか (2018) における郭の議論からすると、既成仏教や神社神道においては、入信というプロセスを経ないでも、それらの宗教の信仰者といえる。それと同様、キリスト教も、一般の日本人の意識からすると、もしも洗礼というプロセスが不要であれば、仏教や神道と同様、三つ目の信仰の対象としうるということになるからである。要するに、その場合には「0.8%」というのは見かけ上の数値に過ぎないということになるからである。

そこでトリプルな信仰の有無について問う質問としてどのようなものを用意したかについていくつか例を述べる。前後の議論とやや重複するかもしれないが、例えばキリスト教主義学校設置目的の推測を問うQ34においては選択肢9として「信者にならなくても、キリスト教の教えを守ることによって、救われる可能性のある人を増やすこと」を置いた。この MA で選択肢を選んだ人を 1、選ばなかった人を 0 として、Q12G とクロス集計して、キリスト教主義学校への通学経験者が 1 の人が多ければ、トリプルな信仰的な意識の醸成に、キリスト教主義学校は寄与しているという可能性が拓ける。

また本稿では、幼少期から原家族でキリスト教以外の宗教行事にどの程度参加してきたかを聞いている(Q3)。もしも Q12G とクロス集計することで、もしもキリスト教主義学校通学経験者の方が仏教や神道の宗教行事にも参加してきたという結果が出れば「トリプルな信仰」の存在をより強く裏づけることになろう。また Q49 では仏教、神道、キリスト教、イスラム教の四つの宗教について「宗教において、入信していなくても教えを守ることによって、救済やご利益（ごりやく）が得られると思いますか。当てはまるものをそれぞれ選んでください」という設問をしている。このキリスト教に関して「そう思う」という人がキリスト教主義学校通学経験者の方に多いとすれば、やはりトリプルな信仰的な意識の醸成に、キリスト教主義学校は寄与しているという可能性が拓けるといえる。

このようにトリプルな信仰に関わるこれらの設問を従属変数として、Q12G を独立変数としてクロス集計することを作業仮説3とする。

なお、今回、インタビュー調査からの引用、並びに定性的な内容の分析を、これらウェブモニター調査の報告に交えている。しかしコロナ禍及び研究代表者の非力による研究の進捗の停滞もあって、まだインタビュー調査並びに各種コンテンツ及び文献の内容の分析は一定の仮説を証明する段階に至っていない。今回、これらの一部を引用しているが、それらはあくまでもウェブモニター調査報告の内容を具体的に肉付けするためのものであり、これら二種の調査の結果報告ではないことを申し添えておく。

### 1.5 本研究の方法、等

本稿の報告は科研費研究のウェブモニター調査が中心であり、そこでの調査方法は 2. 「ウェブモニター調査の概要」の章に委ねる。なおウェブモニター調査については分析方法に限っては、本節で記す。

ウェブモニター調査の結果報告に用いたインタビュー調査は、機縁法でインタビュイー（インフォーマント）をみつけている。今回 3 名についてのインタビュー結果を載せているが、B さんについては、ウェブモニター調査のプレ調査として倫理審査を受け筑波大学図書館情報メディア系倫理審査委員会の承認を得たものである。その倫理申請の際に提出した同意書において、プレ調査ではあるが調査結果を論文に使う場合もあるが、その場合には掲載の前に、内容を示し、事前にインタビュイーの許可を貰う旨、記してある。インタビュー調査前に B さんに同意書に署名してもらい、今回の論文投稿前に、内容を確認いただき、掲載の同意書も得ている。A さん、C さんについては、ウェブモニター調査のプレ調査兼インタビュー調査という形で倫理申請をし、筑波大学図書館情報メディア系倫理審査委員会の承認を得ている。A さん、C さんについても、インタビュー調査前に同意書に署名してもらい、さらに今回の投稿に際し、B さん同様、内容を確認いただき、掲載の同意書ももらっている。なお、A さん、B さん、C さん共に、調査時、20 歳を越えている。

このようにウェブモニター調査のプレ調査を兼ねたインタビュー調査なので、スノーボールサンプリングではなく機縁法でインタビュイーを得ているし、分析もグラウンデッド・セオリーやKJ法やベレルソン流の狭義の内容分析法によるのではなく、定性分析、つまり文学研究や思想哲学研究でテキストを読み込むのと同様の方法で分析し、あくまでもウェブモニター調査の結果分析の肉付け・補足として用いている。ただしウェブモニター調査の結果に対してそれに添う内容以外の発言も含め、事象を立体的・複合的に把握できるように努めた。COREQ のチェックリストの Data analysis に準じる記載をしておくと、24 番のコーダーの数であるが、そもそもコーディングをして積み上げる形をとっていないので該当せずというか0である。25 番のコーディングツリーについても該当せずである。26 番の、テーマが予めあったかこれらに由来するかについては、科研費申請時のテーマは当然これらのインタビュー前のものであるので、その点では「予めあった」といえるが、今回はウェブモニター調査のプレ調査を兼ねたインタビューであるので、ウェブモニター調査の作業仮説はこのインタビューから派生している部分もある。27番のソフトウェアは何を使ったかは該当しない。28 番のインタビュイーが知見をチェックさせたかについては、findingsという形で分離して示してはいないが、本論文草稿全体を渡し、インタビュイーの発言箇所を青字に記し、どことどこでそのインタビュイーの発言に言及したかの一覧表も渡し、さらに掲載についての承諾書も得ている。

上述の、「文学研究や思想哲学研究でテキストを読み込むのと同様の方法で分析し、あくまでもウェブモニター調査の結果分析の肉付け・補足として用いている」という点はウェブコンテンツ、各種文献資料についての広義の内容分析の場合もほぼ同様である。当初はキリスト教主義学校のウェブページやキリスト教主義学校の〇〇年史を系統的に収集し、それらの傾向を量的な内容分析で測定しようと考えていたが、それらの進捗は芳しくはない。今回これらはあくまでもウェブモニター調査の補足として用いているに過ぎない。

このように「文学研究や思想哲学研究でテキストを読み込むのと同様の方法で分析し、あくまでもウェブモニター調査の結果分析の肉付け・補足として用いている」のは、本稿の筆頭著者が思想・哲学寄りの社会学者であることが大きい。そして 1.3 の先行研究の箇所で多く名前を出した、 井上ほか (2000) の井上順孝は宗教学、 井上ほか (2018) の井上章一は風俗論、 佐藤 (2006) の佐藤八寿子は教育社会学、 濱田 (2001)の濱田陽は宗教学である。風俗研究は色々な分野からのアプローチは可能であるが、社会学を日本では初期に世態学と訳したように（東京帝大の最初の Sociology の授業は美学者フェノロサによる「世態学」であった）社会学との繋がりは弱くない。そして社会学と宗教学は元々哲学から分かれた分野であり、本稿の先行研究として挙げた研究においても思想的な議論が多くなされている。また 1.4 でウェブモニター調査の分析の軸にすると述べた、 井上ほか (2018) のトリプルな信仰という言葉は、丸山真男のキリスト教についての見解と大いに反することも、1.3 で述べたとおりである。また本稿 5. での「考察」の議論をやや先取りする感があるが、考察においても ICU の 50 年史の『未来をきり拓く大学―国際基督教大学五十年の理念と軌跡―』(2000) が、著名な思想史研究者の武田清子の単著のものであったので、思想の科学研究会のオリジナル・メンバーとして武田の古くからの友人である丸山の議論との接点も「考察」において記すこととした。

このように思想史的な文脈での研究報告であり、また 1.3 でウェブモニター調査の調査票の選択肢を決める際も、先行研究の議論を踏まえているので、今回は多変量解析等はせずに、ウェブモニター調査の集計・分析もクロス集計に留めることとした。なお今後本ウェブ調査のより多くの変数を取り上げながら多変量解析することも視野に入れている。

具体的には 1.4 で記したように、キリスト教主義学校への関与度を示す Q12 あるいは皇族女子のキリスト教主義学校への関与への是非を問う Q11 あるいはそれをグループ化した変数を、分析の軸にする独立変数と考える。まず主な従属変数としては、キリスト教主義学校設立の目的への推測を問う Q34 とキリスト教主義学校の生徒・学生のイメージを問う Q29 を想定している。これら Q34 と Q29 は選択肢のうち最もそう思うもの 1 つを選ぶ SA と、そう思うものすべてを選ぶ MA、双方を聞いている。いずれも選択肢の数が多いので、SA はクロス集計に使うには度数が低い。したがって、MA でそれぞれの選択肢を選んだ人を 1、選ばなかった人を 0 とする離散変数を各選択肢ごとに想定し、それらを従属変数とし、Q12Gや Q11 を独立変数としてクロス集計をした。1.3 でみた先行研究に照らして各選択肢が選ばれているので、選択肢ごとの分析に努めた。Q34 については 3.1 で、Q29 については 3.2 で記述する。あと 1.4 で示したトリプルな信仰についての従属変数も Q12G とのクロス集計をした。これについては 3.3 で記される。

## 2. ウェブモニター調査の概要

本研究では株式会社サーベイリサーチセンターに委託したウェブモニター調査を実施した。なお、それに先立ち、筑波大学図書館情報メディア系倫理審査委員会の承認を得た（「科研費挑戦的萌芽研究「キリスト教主義学校から見る寛容と洋化―ステークホルダーらの期待と文化資本」(2018~2023 年度（代表:後藤嘉宏））のウェブモニター調査」 （通知番号第 19-99 号））。調査対象者の属性は東京都・神奈川県に在住の 15 歳 ~29 歳の男女とした。

同意はウェブモニター管理会社楽天インサイトの同意画面によって当人の意思確認をする形によって求めた。ウェブモニター管理会社楽天インサイトのデータ収集と同意を含むすべての手続きについて、筑波大学図書館情報メディア系倫理審査委員会の承認を得ている。

調査は 2020 年 2 月 22 日に開始し、予定のサンプル数 700 に達した 3 月 3 日に終了した。なお、サンプル数は多ければ多いほど統計誤差が減るので望ましいが、この 700 という数値は、今回必要とされる、用意した質問数において今回使える予算の上限額目一杯のサンプル数であるので、この数値にした。

東京都と神奈川県に居住地域を絞ったのは、これらの地域にキリスト教主義学校が多いからである。特に神奈川県はフェリス女学院等明治時代初期からキリスト教主義学校があっただけでなく、戦後 GHQ の政策で、旧海軍用地や農地改革等で接収した土地を横須賀の進駐軍との絡みで、キリスト教主義学校の校地のために優先的に払い下げたとの説もある（ 島本 1994）。以下の数字にカトリックは含まれないし、プロテスタントでも漏れはあるものの、 一般社団法人キリスト教学校教育同盟の一覧では東京都 23 学校法人、神奈川県 11、愛知県 4、大阪府 7、兵庫県 8、福岡県 4 で、他の都道府県は 3 学校法人以下である （最終確認日 2022 年 10 月 17 日）。

キリスト教主義学校が身近にない調査対象者だとイメージを浮かべにくい点が、これらの地域の住民に限定した理由である。さらにそのことと関連するが、それ以上に、本調査全体の枠組みにもこの限定は関わっている。再三申し上げているように本調査ではキリスト教主義学校に通ったことがある・あるいはいま通っている当事者をいわばキリスト教主義学校当事者として、それらの者の保証人等家族や志望したことがある者、兄弟姉妹に通学者のいる者、オープンキャンパスに行ったことのある者などを、当事者の周辺の者・緩やかな当事者とし、これらに該当しない者を部外者の者として、これら三者の間での意識の差の有無をみようとしている。これらの差を本調査の他の質問とのクロス集計の分析の軸として想定した。よって当事者や当事者周辺の者（緩やかな当事者）を一定数以上確保するためには、キリスト教主義学校が通学圏にほとんどない地域の調査対象者を減らすことが、有効と考えた。

具体的には調査票 Q12 で「あなたのキリスト教主義学校への関与についてお聞きします。当てはまるものを選んでください」という設問をして、このキリスト教主義学校関与度を測定しようとしている。この設問の単純集計の結果は 表2-1のようになっている。

**表2-1.** キリスト教主義学校への関与について. Q12. あなたのキリスト教主義学校への関与についてお聞きします。当てはまるものを選んでください。

選択肢	回答数	割合
1. 通ったことがある	108	15.4%
2. 通ったことは一度もないが、受験したことがある	55	7.9%
3. 受験したことはないが、条件（学力、経済力、交通の便等）が見合えば受験してみたい学校があった、あるいは今現在ある	42	6.0%
4. 受験候補に加えたことは1度もないが、オープンキャンパスあるいは公開講座に行ってみたいと思ったことがある、あるいは実際に行った（向こうからあなたの在籍校に教えに来る、いわゆる「出前授業」「出張講義」は含めません）	23	3.3%
5. 自分自身は通ったことはないが、家族に通った者がいる	48	6.9%
6. 上記のいずれにも該当しない	424	60.6%
合計	700	100.0%

これらのうち、選択肢 1 の「通ったことがある」を選んだ者、選択肢 2 から5までを選んだ者、選択肢 6 の「上記のいずれにも該当しない」を選んだ者、これら 3 つのカテゴリーに再グループ化した Q12G という組変数とした。この Q12G を独立変数として、他の諸変数を従属変数にした項目間クロス集計を行い、この Q12G を分析の軸にすることを想定した。要は上述のように、キリスト教主義学校に通ったことがある者、通ったことはないが何らかの当事者性のある者、関与のまったくない者の3カテゴリーに分けた Q12G を分析の軸とすることで、キリスト教主義学校関与者とそれ以外との意識の差が測れると考 えた ^12^。

次に 15~29 歳の若年層に調査対象者を絞った理由は、まだ高校受験・大学受験・大学院受験の可能性のある者や、可能性は少なくなったが、進学を控えていた頃の記憶がまだ鮮明に甦る世代の意識を聞いてみる必要性を感じたからである。ただし今後、ほぼ類似の質問票で世代を替えて調査し、それと比較する可能性も想定してはいる。

なお、今回の調査対象者の基本属性は 表2-2 のようになる。

**表2-2.** 調査協力者の基本属性.

選択肢	回答数	割合
全体	700	100.0%
男性	362	51.7%
女性	338	48.3%

選択肢	回答数	割合
全体	700	100.0%
15~19 歳	184	26.3%
20~24 歳	149	21.3%
25~29 歳	367	52.4%

選択肢	回答数	割合
全体	700	100.0%
東京都	432	61.7%
神奈川県	268	38.3%

また今回の回答者の信仰する宗教についての設問の回答は 表2-3 のようになっている。これは、Q1-9「あなたの信仰する宗教に当てはまるものを選んでください」という設問の回答（SA）である ^13^。

**表2-3.** 調査協力者の信仰.

番号	選択肢	回答数	割合
1	神社神道	35	6.2%
2	既成(伝統)仏教(南都六宗、真言宗、天台宗、禅宗、浄土真宗、日蓮宗、等)	101	17.8%
3	既成(伝統)キリスト教(正教会、カトリック、プロテスタント、等)	17	3.0%
4	神道系の新宗教(PL教、天理教、大本教、等)	3	0.5%
5	仏教系の新宗教(霊友会、立正佼成会、幸福の科学、創価学会、等)	18	3.2%
6	キリスト教系の新宗教(ものみの塔(エホバの証人)、モルモン教、世界平和統一家庭連合(旧略称:統一教会)等)	5	0.9%
7	上記4,5,6に入らない新宗教	0	0.0%
8	上記1~7に入らない宗教	4	0.7%
9	特にない	301	53.2%
10	よく分からない	66	11.7%
11	答えたくない	16	2.8%
	計	566	100.0%

「よく分からない」の 11.7%、「答えたくない」の 2.8% を含めた中で、また「特にない」も 53.2% もある中で、「既成（伝統）キリスト教 ^14^」3.0%、「キリスト教系の新宗教」0.9% であわせると 3.9% に及び、全国平均のクリスチャンの比率 0.8% よりだいぶ大きい数値となっている。ただ Q12 に替えてクロス集計の軸にできるほどには大きい数値ではない。なおウェブモニター調査の性格上、モニター登録者のうち、このテーマに興味をもった者が答えるということから多めの数値になった可能性もある。

「よく分からない」「答えたくない」「特にない」を合わせると 68.0% もいて、残りの 3 割のみが信仰をもつということは少ない数字とも感じられるが、井上順孝の『「現代日本における宗教教育の実証的研究」(1998~1999) 報告書』と較べるとむしろ大きい数字であるともいえる。同研究報告書では「信仰あり」が22.8% である（ 井上ほか 2000 p.40)。ただし同研究の調査票は本研究に比して、この数値が低く出る設計となっている。「あなたは宗教にどの程度関心がありますか。次のうちから選び、さらにそれぞれの質問に答えてください」という質問文で、枝問はとりあえず措いて選択肢は以下の四つである。「1. 現在、信仰をもっている　2. 信仰をもっていないが、宗教に関心がある　3. 信仰をもっていないが、宗教にもあまり関心がない　 4. 信仰はもっていないし、宗教にもまったく関心がない」。

この 井上ほか (2000) の設問は、質問文の方が「関心」のみを聞いているのに、上記の選択肢は「信仰」の有無と「関心」の有無が混ざっている。本来「信仰」と「関心」は別次元であるので、別々に問うかファセット構造の質問にすべきで、おそらくファセット構造を目指しつつやや中途半端に終わっていると思われる。なぜならファセットなら質問文で「信仰」と「関心」の双方を聞く形に書くべきなのに「関心」のみを問うているし、選択肢の方もファセットで排他的かつ網羅的に回答者が選べるようにするには、「信仰をもっているが、宗教に関心がない」という選択肢が必要だが、それがない。また「1. 現在、信仰をもっている」はやや諄くなるがファセットであれば「1. 現在、信仰をもっているし、宗教に関心がある」の方がよい。したがってファセット構造が不充分であるので、幼児洗礼を受けたからキリスト教徒であるとか親が仏教徒だから何となく仏教を信じてはいるという者が、「信仰あり」から排除される可能性が生じる。

先ほどこの 井上ほか (2000) の枝問についてはとりあえず措くとしたが、選択肢 1.「現在、信仰をもっている」を選んだ場合、7 つの枝問を答えるように指示され、それらの枝問の 2 問目は、「その宗教には何歳のときに入信しましたか」とある。したがって何となく神道の信者であるとか、葬式仏教といわれるような先祖代々の宗教だから一応仏教の宗派を信じている人は、枝問まで眼に入ると、この本問の方の選択肢 1. 「現在、信仰をもっている」を選びにくくなっているのが、『 「現代日本における宗教教育の実証的研究」(1998~1999) 報告書』の調査票であるといえる。なおこのように自覚的な信仰のある者に信仰のある人を絞り込むことはこの研究であえてそうしている可能性があり、そのことで、この研究では宗教系高校等の入信に対する効果を測定できている側面もあることを付言しておく。

したがって我々の調査の調査対象者で信じている宗教を答える者が『 「現代日本における宗教教育の実証的研究」(1998~1999) 報告書』よりやや多いのは、ある意味当然ともいえる。

ただ 井上ほか (2018) の「トリプルな信仰」の根拠は神道と仏教合わせて信徒数が日本の総人口を超えるということにあったが、 井上ほか (2000) も我々も、それに比して信仰を持つ人が少ない。今後は自覚的な信仰と、親の信仰を何となく嗣ぐ場合のような緩やかな信仰を分けつつ、双方をきき取る調査票を設計したい。　

なお、ウェブモニター調査だと無作為抽出法の調査と違い、サンプルが母集団のほぼ正確な縮図になることはないという限界があることは我々も承知している。しかし、宗教に関する設問で郵送法・留置法だと回収率が期待できず、かといって新型コロナウイルスが流行りはじめた時期に、訪問面接法で、家の軒先で 40 分前後のやり取りを行うことは多くの調査対象者から敬遠されたと考えられる。また紙の調査とウェブモニター調査とを比較すると、それぞれに利点と欠点とがあり、紙の調査の回収率の低下している現代ではウェブモニター調査が学術目的の調査として有効ではないとは必ずしもいえず、特にセンシティブな内容の調査にはウェブモニター調査が適しているとの、 日本学術会議社会学委員会の報告もある（最終閲覧日 2022 年 10 月 17 日）。

なお今回、調査委託先のサーベイリサーチセンターを通じて、ウェブモニターの管理会社楽天インサイトの意向として、答えにくい設問については「よくわからない」や「答えたくない」の選択肢をつけるように指示されたため、無回答故の DKNA 以外に、「よくわからない」や「答えたくない」の選択肢の含まれる設問もある。なお、調査票は拡張データに含まれている ( Goto, 2022b)。

この章の最後に STROBE のチェックリスト 12 番の Statistical methods についての記載をしておく。a)「交絡の制御に用いる方法を含む、すべての統計的手法を説明する」であるが、諸変数の交錯した影響を統制しつつ見るには多変量解析が最適であることは承知しているが、本稿は、まずは先行研究で指摘された事項それぞれを選択肢にし、それが妥当するかどうかを丁寧にみていくことが重要かつ先決な作業であると考え、クロス集計による分析に留めている。したがって、ここはクロス集計とカイ二乗検定という古典的手法のみによったという答えになる。b)「サブグループと相互作用を調べるために使用された方法を説明する」についてであるが、Q12 というキリスト教主義学校関与度によって全回答者をキリスト教主義学校の関与度でサブグループに分けてクロス集計した。c)「欠損データにどのように対処したかを説明する」についてであるが、欠損データは「無回答」として処理した。また基本的に上述のように「よくわからない」や「答えたくない」をあえて選択肢に設けた設問も多いので、「無回答」も生じにくい。d) については「コホート研究」「症例対照研究」「横断的研究」それぞれに該当する場合の質問がチェックリストにあるが、このいずれにも該当しない。e) は「感度分析があれば説明する」であるが該当しない。

## 3. 調査結果とその分析

以下で調査結果とその分析を記すが、説明の都合上 2.1 の①から⑥の順ではなく、順序は前後する。

### 3.1 キリスト教主義学校の設置目的について

表3-1-1 は「Q34. あなたが思う、キリスト教主義学校に対する設置母体のメリットに当てはまるものを上位3つまでの間で選び、中でも最もメリットだと思うものを 1 つ選んでください」と MA と SA 双方で聞いた質問の回答である。

**表3-1-1.** キリスト教主義学校の設置母体のメリット(MA、SA).

選択肢	メリットだと思うもの (上位3つまで)		最もメリットだと思うもの (1つだけ)	
	n	%	n	%
信者の子弟の教育要求への対応	94	7.7	44	6.3
教団組織の資金集め	124	10.2	69	9.9
教会以外の、キリスト教に触れる敷居の低い舞台としての役割を果たすこと	105	8.7	53	7.6
同じ教団組織の日本以外の支部に対して、日本支部の活動実績を提示すること	46	3.8	10	1.4
教育を通じた地域貢献活動を行い、イメージの向上を図ること	90	7.4	37	5.3
海外からキリスト教徒が移住してきた際の、子弟の教育受け入れ先としての役割を果たすこと	59	4.9	20	2.9
キリスト教への共感者（シンパサイザー）を増やすこと	76	6.3	21	3.0
キリスト教の信者を増やすこと	91	7.5	37	5.3
信者にならなくても、キリスト教の教えを守ることによって、救われる可能性のある人を増やすこと	93	7.7	55	7.9
社会的影響力のある卒業生を増やすこと	54	4.5	18	2.6
自分たちの死後の救済を得るために、奉仕の精神で教育を行うこと	25	2.1	9	1.3
自分たちの信仰の意味を違った文化圏で問い直せること	44	3.6	15	2.1
その他	3	0.2	3	0.4
答えたくない	10	0.8	10	1.4
よく分からない	299	24.6	299	42.7
計	1213	100	700	100

先述のように本調査では設問により「答えたくない」や「よく分からない」を入れることを委託業者から求められたが、この設問では選択肢の 15 においた「よく分からない」を選んだ者が 42.7% と最も多かった。

この選択肢を選ばなかった者を 0、選んだものを 1 として従属変数にして、キリスト教主義学校当事者指数を 3 段階にグループ分けした Q12G を独立変数として、両者をクロス集計する（ 表3-1-2）。

**表3-1-2.** キリスト教主義学校関与度×Q34選択肢「よくわからない」.

		合計	よくわからないを選ばなかった	よくわからないを選んだ
全体		700	401	299
		100.0%	57.3%	42.7%
Q12G. あなたのキリスト教主義学校への関与についてお聞きします。当てはまるものを選んでください。	通ったことがある	108	85	23
		100.0%	78.7%	21.3%
	通ったことはないが、関わったことがある・関りのある人がいる	168	115	53
		100.0%	68.5%	31.5%
	上記のいずれにも該当しない	424	189	235
		100.0%	44.6%	55.4%

キリスト教主義学校に通ったことがある者は「よく分からない」を選ぶ者が相対的に少なく21.3%、通ったことはないが緩やかな当事者性のあるグループが中間で 31.5%、関与のまったくない者は「よく分からない」を選ぶものが 55.4% に及んでいる(カイ二乗<0.01)。当事者性の強い者ほど、学校側の考えるものと合致しているか否かは措いて、キリスト教主義学校の設置母体の目的を何らかの意味で意識していることが、この結果から分かる。それと同時に、キリスト教主義学校の在校生・通学経験者やその周辺部分以外、つまり当事者性のない者の過半の者から、キリスト教主義学校の理念あるいは場合によってその存 在自体がまったく意識されていない可能性も、この結果から示唆される。もっとも通ったことがある者でも「よく分からない」を選ぶ者が 21.3% いたが、進学・就職実績のみを理由に進路に選んだ者も相当数いるはずで、それがこの数値に示されていよう。

この「よく分からない」を除いた選択肢の度数の多い方から並べた図は 図3-1-1 のようになる。

**図3-1-1.**
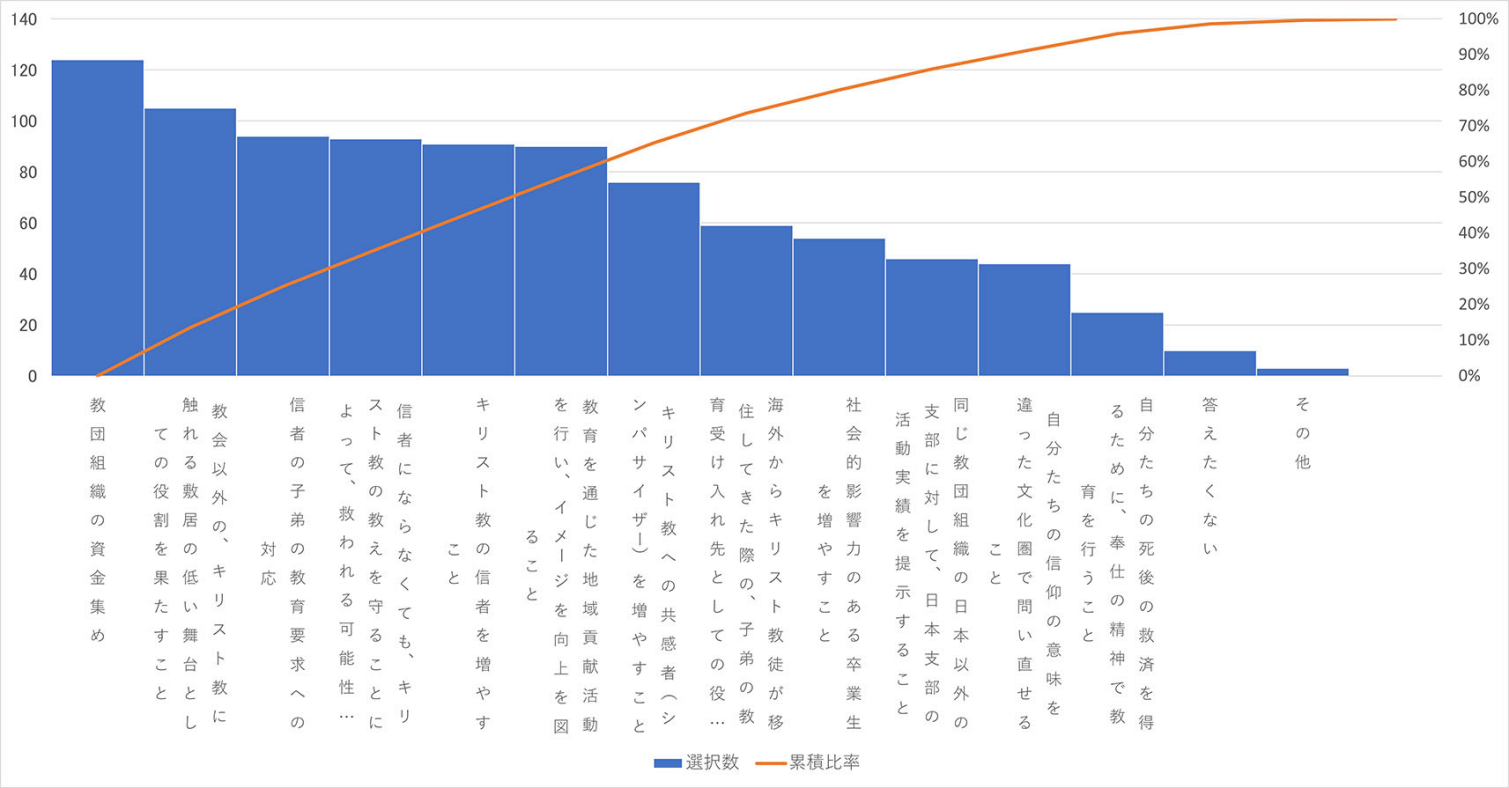
Q34「設置母体のメリット」の選択肢のMAで選ばれた数(度数順).

この Q34の選択肢2.「教団組織の資金集め」が MA で 17.7% とつぎに多い、いいかえると「よく分からない」を除くと実質、一番多い。私立学校で理事や教職員が一般の俸給生活者より遥かに多額の報酬を得ることはあるにせよ、学校法人の法的性格から法人が上げた利益を外部に流すことは通常ないし ^15^、またキリスト教主義学校の実態からも現実にはありえない。このようにこちらとしてはありえないはずの選択肢をあえて入れただけのつもりのこの選択肢が、なぜかこの設問の単純集計で実質、最も票を集めた。これとQ12G をクロス集計すると（ 表3-1-3）、関与の全くない者は予想に反して、「教団組織の資金集め」を選ぶ者が 14.4% で、最も少ない。僅差で続くのが、キリスト教主義学校に通ったことがある者で、これらのグループで「教団組織の資金集め」を選ぶ者は 14.8% で、このグループが少ないことは予想の通りであった。一方、通ったことはないが何らかの当事者性のあるグループが、意外にもこれら3カテゴリー中で最も多くこの「教団組織の資金集め」を選んでいて、28.0% いた（カイ二乗<0.01）。

**表3-1-3.** キリスト教主義学校への関与×Q34選択肢「教団組織の資金集め」.

		合計	教団組織の資金集めを選ばなかった	教団組織の資金集めを選んだ
全体		700	576	124
		100.0%	82.3%	17.7%
Q12G.あなたのキリスト教主義学校への関与についてお聞きします。当てはまるものを選んでください。	通ったことがある	108	92	16
		100.0%	85.2%	14.8%
	通ったことはないが、関わったことがある・関りのある人がいる	168	121	47
		100.0%	72.0%	28.0%
	上記のいずれにも該当しない	424	363	61
		100.0%	85.6%	14.4%

特に Q12 をグルーピングする前の形で分布をみると、「通ったことはないが、受験したことはある」者が40.0%と特に多くなっている。なお、各セルの実数は少ないがこの表もカイ二乗<0.01 となっている （ 表3-1-4）。

**表3-1-4.** キリスト教主義学校への関与（グルーピング前）×Q34選択肢「教団組織の資金集め」.

		合計	教団組織の資金集めを選ばなかった	教団組織の資金集めを選んだ
全体		700	576	124
		100.0%	82.3%	17.7%
Q12.あなたのキリスト教主義学校への関与についてお聞きします。当てはまるものを選んでください。	通ったことがある	108	92	16
		100.0%	85.2%	14.8%
	通ったことはないが、受験したことはある	55	33	22
		100.0%	60.0%	40.0%
	受験したことはないが、受験したい学校がある	42	32	10
		100.0%	76.2%	23.8%
	受験候補ではないが、行ってみたい・行ったことがある	23	17	6
		100.0%	73.9%	26.1%
	通ったことはないが、家族に通ったものがいる	48	39	9
		100.0%	81.3%	18.8%
	上記のいずれにも該当しない	424	363	61
		100.0%	85.6%	14.4%

受からずに受験料のみ徴収された者や、合格して入学金を納めたものの志望順位のより高い学校に受かりキリスト教主義学校に通わなかった者が、「通ったことはないが、受験したことはある」となることを考慮すると、実際に通った者と、それ以外の緩やかな当事者性のある者との間にある、この温度差は、納得もゆく。

次に選択肢 3「教会以外の、キリスト教に触れる敷居の低い舞台としての役割を果たすこと」を Q34 の MA で選んだ者は全体で 15.0% である。これについては、「よく分からない」を除いて Q34 の MA の単純集計で 2 番目になる。Q12G とクロス集計するとキリスト教主義学校に通った者はこの選択肢を 28.7% 選んでおり、ゆるやかな当事者は 22.6%、他方、まったく関与しない者は 8.5% である（カイ二乗<0.01） （ 表3-1-5）。

**表3-1-5.** キリスト教主義学校への関与×Q34「教会以外のキリスト教にふれる舞台」選択肢.

		合計	教会以外の、キリスト教に触れる敷居の低い舞台としての役割を果たすことを選ばなかった	教会以外の、キリスト教に触れる敷居の低い舞台としての役割を果たすことを選んだ
全体		700	595	105
		100.0%	85.0%	15.0%
Q12G.あなたのキリスト教主義学校への関与についてお聞きします。当てはまるものを選んでください。	通ったことがある	108	77	31
		100.0%	71.3%	28.7%
	通ったことはないが、関わったことがある・関りのある人がいる	168	130	38
		100.0%	77.4%	22.6%
	上記のいずれにも該当しない	424	388	36
		100.0%	91.5%	8.5%

これはキリスト教主義学校の当事者性の大小ときれいに相関する。この選択肢3は「信者にならなくても、キリスト教の教えを守ることによって、救われる可能性のある人を増やすこと」の選択肢9にも近いが、選択肢 7 の「キリスト教への共感者（シンパサイザー）を増やすこと」とも関係しうる。また先述のようにキリスト教式結婚式に関する 濱田 (2001) による、信仰への入口と一部の聖職者側が考えているという議論からも、この選択肢をキリスト教主義学校への通学者が多く選んだことは納得がゆく。やはり先述のように濱田の研究において日本のカトリック中央協議会が1973 年バチカンに示した文書で、カトリック学校の卒業生にキリスト教式結婚式の利用希望者が多いと記されていた（ 濱田 2001 p.29）。このようなキリスト教式結婚式と同様の機能をキリスト教主義学校が果たしているとも、考えられる。

Q34の選択肢1は「信者の子弟の教育要求への対応」であるが、これを選んだ者は MA での集計の全体で13.4% で、「よく分からない」を除いて MA の単純集計で 3 番目である。キリスト教主義学校に通ったことがある者は「信者の子弟の教育要求への対応」を選ぶ者が相対的に多く22.2%、通ったことはないが何らかの当事者性のあるグループが中間で 19.0%、関与のまったくない者は「信者の子弟の教育要求への対応」を選ぶものが 9.0% に留まっている（カイ二乗<0.01） （ 表3-1-6）。

**表3-1-6.** キリスト教主義学校への関与×Q34選択肢「信者の子弟の教育要求への対応」.

		合計	信者の子弟の教育要求への対応を選ばなかった	信者の子弟の教育要求への対応を選んだ
全体		700	606	94
		100.0%	86.6%	13.4%
Q12G. あなたのキリスト教主義学校への関与についてお聞きします。当てはまるものを選んでください。	通ったことがある	108	84	24
		100.0%	77.8%	22.2%
	通ったことはないが、関わったことがある・関りのある人がいる	168	136	32
		100.0%	81.0%	19.0%
	上記のいずれにも該当しない	424	386	38
		100.0%	91.0%	9.0%

これもキリスト教主義学校関与度指数と相関しているといえる。先行研究の『 「現代日本における宗教教育の実証的研究」(1998~1999)報告書』における市川の分析によると、全体でみると、出身高校の宗派は非宗教系高校 87.9%、神道系 0.6%、新宗教系 0.6%、仏教系 3.3%、カトリック系4.2%、プロテスタント系 3.4% で、非宗教系高校が圧倒的に多い（ 井上ほか 2000 p.100）。他方、カトリックの信仰をもつ者についても非宗教系高校が多いことは多いが、58.3% で全体の 87.9% よりは遥かに少ない。そしてカトリック高校は 29.2% と平均の 4.2% より非常に多い。あと全体に自分の信仰と違う宗教系高校は避けると市川は分析するが、例外はカトリックとプロテスタントで、カトリック信者がプロテスタント高校、プロテスタント信者がカトリック高校というケースは少なくないという。「同じキリスト教ということで、相異点よりも共通点の方が強く意識されている」可能性を市川は推測する（ 井上ほか 2000 p.101）。要するに信徒の教育要求に応える機能を、宗教系学校はもっているとこの研究は示唆する。井上順孝らの先行研究から 20 年隔たっているが、基本的に今回の結果も、キリスト教主義学校について似た形になっている。

また Q34の選択肢8は「キリスト教の信者を増やすこと」であるが、この選択肢を選んだ者は全体で13.0% であった。これは「よく分からない」を除き MA の単純集計で 5 番目である。この選択肢を選ばなかった者を 1、選んだものを 0 として従属変数として、Q12G を独立変数として、両者をクロス集計」すると（ 表3-1-7）、非常に残念ながら通常の検定で有意であるとする基準である危険度 0.05 以下では有意差はなくp値=0.1346 の水準であった。だが、キリスト教主義学校に通ったことがある（あるいは通っている）者は「キリスト教の信者を増やすこと」を選ぶ者が少なく7.4%、通ったことはないが緩やかな当事者性のあるグループが中間で 12.5%、関与のまったくない者は「キリスト教の信者を増やすこと」を選ぶものが 14.6% と 3 カテゴリーで最も多くなっている。また選択肢8の「キリスト教の信者を増やすこと」と趣旨として全く逆になるであろう、選択肢 9 の「信者にならなくても、キリスト教の教えを守ることによって、救われる可能性のある人を増やすこと」は、4パラグラフ後に申し上げるように、この選択肢 8 と全く逆の相関関係になっていて、そちらはカイ二乗 <0.01 である。したがって、この選択肢 8 で有意差がなかったのは、検出力不足（データ数の不足）のためであると我々は考察している。

**表3-1-7.** キリスト教主義学校への関与×Q34選択肢「信者を増やすこと」.

		合計	キリスト教の信者を増やすことを選ばなかった	キリスト教の信者を増やすことを選んだ
全体		700	609	91
		100.0%	87.0%	13.0%
Q12G. あなたのキリスト教主義学校への関与についてお聞きします。当てはまるものを選んでください。	通ったことがある	108	100	8
		100.0%	92.6%	7.4%
	通ったことはないが、関わったことがある・関りのある人がいる	168	147	21
		100.0%	87.5%	12.5%
	上記のいずれにも該当しない	424	362	62
		100.0%	85.4%	14.6%

有意差はないとはいえ、キリスト教主義学校の当事者指数の高い者ほど、キリスト教主義学校が信者獲得を目的としていないという認識をもっている可能性が示唆されたことは、興味深い結果である。われわれは、同じ系列のキリスト教主義学校に幼稚園から大学院修士課程まで在籍した男子大学院Aさんにインタビューしたことがあったが、彼は高校時代、洗礼を考えたことがあり、宗教担当の教員に相談までしたが、急ぐことはないのでゆっくり考えるようにいわれたとの発言をしているが（調査日 2019 年 5 月 31 日）、そういう発言と、この調査結果は照合しうる。なお A さんの調査に先立ち、筑波大学図書館情報メディア系倫理審査委員会より「科学研究費（萌芽）「キリスト教主義学校から見る寛容と洋化―ステークホルダーらの期待と文化資本」 （2018~2018 年度（代表:後藤嘉宏））の質的調査並びにウェブモニター調査のためのプレ調査」（通知番号第 19-10 号）の承認を得ている（以下、A さんの発言の引用の際は、この倫理審査についての記述を重複を避けるため控える）。

キリスト教主義学校を通常、ミッションスクールと称するが、ミッションスクールではないと謳うキリスト教主義学校も少なくない。同志社などもその例ではある。例えば佐藤八寿子は「同志社は、キリスト教主義の学校ではありますが、キリスト教の伝達を目的とするミッションスクールではないのです」という同志社総長の入学式式辞に言及しながら、多くのミッションスクールが設立当初のキリスト教伝道団体から独立した日本のキリスト教主義の学校として運営されていると指摘する（ 佐藤 2006 pp.8-9）。

他方、1951 年にはプロテスタント系の基督教学校教育同盟理事会が、キリスト教主義学校と呼称する申し合わせをしたが、キリスト教伝道団体から独立しているものの教育方針はそれ以前と同様であるため、ミッションスクールの呼称を容認する学校もあるという（ 佐藤 2006 p.11）。またミッションには「布教」と同時に「使命」の意味もあり、神に与えられたそれぞれの使命を生徒が果たせるような教育を行うという意味で、ミッションスクールを名乗っているカトリックの学校、長野清泉女学院の例も佐藤は紹介している （ 佐藤 2006 pp.12-13） ^16^。しかしミッションスクールのミッションは通常日本では「布教」を意味する。キリスト教徒を増やすというこの狭い意味での「ミッション」の意図を表明している学校の少ないことは、前段落の佐藤の説明からも分かるが、それとともに、このような当事者と非当事者との比較からも一応示唆されるであろう。

他方 Q34の選択肢9は「信者にならなくても、キリスト教の教えを守ることによって、救われる可能性のある人を増やすこと」である。順番は前後するが、これは全体で13.3%で「よく分からない」を除いて MA の単純集計で4番目である。この選択肢は、選択肢 8 の「キリスト教の信者を増やすこと」が狭義の宣教・布教だとすると、より緩やかな、広義の布教となろう。神から与えられた「使命」を果たすという前々段落で佐藤により示された長野清泉女学院の例もこの類に近いといえるであろう。先にみた Q34.の選択肢 8 の「キリスト教の信者を増やすこと」より若干のみ多い数値であったが、こちらは Q12G とのクロス集計において選択肢 8 とは逆の分布になっている（ 表3-1-8）。

**表3-1-8.** キリスト教主義学校への関与×Q34選択肢「信者にならなくても、教えを守り救われる人を増やす」.

		合計	信者にならなくても、キリスト教の教えを守ることによって、救われる可能性のある人を増やすことを選ばなかった	信者にならなくても、キリスト教の教えを守ることによって、救われる可能性のある人を増やすことを選んだ
全体		700	607	93
		100.0%	86.7%	13.3%
Q12G.あなたのキリスト教主義学校への関与についてお聞きします。当てはまるものを選んでください。	通ったことがある	108	78	30
		100.0%	72.2%	27.8%
	通ったことはないが、関わったことがある・関りのある人がいる	168	149	19
		100.0%	88.7%	11.3%
	上記のいずれにも該当しない	424	380	44
		100.0%	89.6%	10.4%

この選択肢を選ばなかった者を 1、選んだものを 0 として従属変数にして、キリスト教主義学校当事者指数の Q12G を独立変数として、両者をクロス集計すると、有意差<0.01 で、キリスト教主義学校に通ったことがある者はこの選択肢 9「信者にならなくても、キリスト教の教えを守ることによって、救われる可能性のある人を増やすこと」を選ぶ者が相対的に多く、27.8% いた。他方、通ったことはないが何らかの当事者性のあるグループは中間で 11.3%、関与の全くない者は選択肢 9 を選ぶ者が 10.4% であり、この設問の回答はキリスト教主義学校当事者性の大小ときれいに相関する。

1.2.2 で先述のように、 井上ほか (2018) 第 2 章では「宗派に帰属しない日本人のトリプル信仰」 （ 井上ほか 2018 p.136）に着目するが、この結果は、キリスト教主義学校の通学者はこのようなトリプルな信仰を想定している者が相対的に多いことを裏づける結果といえる。同書では「日本社会の「トリプル信者」を生み出す要素はいろいろあるだろうが、重要なのは中学校・高等学校の教育ではないかと思う」（ 井上ほか 2018 p.139）として、キリスト教主義の高校が全国の 4.2% と、キリスト教徒の比率よりは多く、しかも名門校が多いことも関連するとする。

当然このようなトリプルな信仰は融通無碍で無限抱擁的な宗教の性格に親和性がある。他方、先述の丸山真男『日本の思想』で記されるように、キリスト教が日本において首尾一貫性を重んじる姿勢を貫いているとすれば、このようなトリプルな信仰に通じることはあり得ない、ということになる。

基本的に「信者にならなくても、キリスト教の教えを守ることによって、救われる可能性のある人を増やすこと」を選ぶ者が、キリスト教主義学校出身者に多いので、洗礼のような明確な入信のプロセスよりも、トリプルな信仰の方に、少なくとも比較的若い世代のキリスト教主義学校出身者は親和的であることを示唆している。われわれの行った質的調査において、現在の同志社は「徳育としてのキリスト教」という言葉を強調していると、中学から大学まで同志社に学んだCさんは語っていたが（調査日 2020 年 1 月 28 日）、この同志社の「徳育としてのキリスト教」という教育理念もこの選択肢の趣旨に近い発想であるといえよう。なおCさんの調査に先立ち、A さん同様、筑波大学図書館情報メディア系倫理審査委員会よ り「科学研究費（萌芽）「キリスト教主義学校から見る寛容と洋化―ステークホルダーらの期待と文化資本」 （2018~2018 年度（代表:後藤嘉宏））の質的調査並びにウェブモニター調査のためのプレ調査」（通知番号第19-10 号）の承認を得ている（以下、C さんの発言の引用の際、この倫理審査についての記述は重複を避けるため控える））。なおこのトリプルな信仰については他の設問にて聞いているので 3.3 で詳述する。

ここまでみてきたこれら Q34 の選択肢 1，3，8，9 のそれぞれの単純集計及びそれらと Q12G とをクロス集計した結果を総合して考えてみると、キリスト教主義学校は、新たな信者を増やす場としてよりは、キリスト教的な徳育をする場として当事者たちから受け取られていると同時に、すでに信者である者の子弟に好個の教育施設としても位置づけられているといえる。そして狭義の布教（ミッション）をするよりはトリプルな信仰に通じる広義の布教(ミッション)をする場、さらには布教（ミッション）よりも神から与えられた使命（ミッション）を自覚させる場として意識されているといえる。要するにミッションという言葉の拡がり、偏差、バリエーションのなかで、狭義の意味以外のこの言葉の意味を選択している・あるいは模索していることが示唆されるといえよう。

選択肢 9 についで MA で多いのは選択肢 5 の「教育を通じた地域貢献活動を行い、イメージの向上を図ること」で、全体で 14.9% である。これを Q12G とクロス集計すると 表3-1-9 のようになり、カイ二乗<0.01で有意差がある。キリスト教主義学校非該当者の方が、「教育を通じた地域貢献活動を行い、イメージの向上を図ること」を選ぶ者が少ない。他方実際に通ったことのある人と緩やかな当事者で比較すると、緩やかな当事者の方が「教育を通じた地域貢献活動を行い、イメージの向上を図ること」を選ぶ者がやや多い。

**表3-1-9.** キリスト教主義学校への関与×Q34「教育を通じた社会貢献活動によるイメージ向上」.

		合計	教育を通じた地域貢献活動を行い、イメージを向上を図ることを選ばなかった	教育を通じた地域貢献活動を行い、イメージを向上を図ることを選んだ
全体		700	610	90
		100.0%	87.1%	12.9%
Q12G.あなたのキリスト教主義学校への関与についてお聞きします。当てはまるものを選んでください。	通ったことがある	108	89	19
		100.0%	82.4%	17.6%
	通ったことはないが、関わったことがある・関りのある人がいる	168	131	37
		100.0%	78.0%	22.0%
	上記のいずれにも該当しない	424	390	34
		100.0%	92.0%	8.0%

緩やかな当事者の分布の実情を見るため、組変数にせずに Q12 そのものとクロス集計してみると、 表3-1-10のようになる（カイ二乗<0.01）。「受験してみたい」「受験したことがある」者にこれを選ぶ者が多く、 「通ったことはないが、家族に通ったものがいる」者は非当事者とほぼ結果が等しいほどに、これを選ぶ者が少ない。要するに、この選択肢についてはイメージ先行で、当人あるいは家族が通学していない者から選ばれやすいということになる。

**表3-1-10.** Q12 キリスト教主義学校への関与(グルーピング前) × Q34「教育を通じた社会貢献活動によるイメージ向上」.

		合計	教育を通じた地域貢献活動を行い、イメージを向上を図ることを選ばなかった	教育を通じた地域貢献活動を行い、イメージを向上を図ることを選んだ
全体		700	610	90
		100.0%	87.1%	12.9%
Q12.あなたのキリスト教主義学校への関与についてお聞きします。当てはまるものを選んでください。	通ったことがある	108	89	19
		100.0%	82.4%	17.6%
	通ったことはないが、受験したことはある	55	40	15
		100.0%	72.7%	27.3%
	受験したことはないが、受験したい学校がある	42	28	14
		100.0%	66.7%	33.3%
	受験候補ではないが、行ってみたい・行ったことがある	23	20	3
		100.0%	87.0%	13.0%
	通ったことはないが、家族に通ったものがいる	48	43	5
		100.0%	89.6%	10.4%
	上記のいずれにも該当しない	424	390	34
		100.0%	92.0%	8.0%

この選択肢 5 についで MA で多いのは選択肢7の「キリスト教への共感者（シンパサイザー）を増やすこと」で全体で 10.9% である。また 井上ほか (2018) ではキリスト教主義学校がどの社会階層をターゲットにするかという議論が過去にあったことを記しているが、そのこととも関係するといえる。カトリックは当初、救貧、慈善活動を強く意識し、孤児院の延長で白百合学園などの学校の前身施設を設立していったが （ 井上ほか 2018 pp.191-192）、正規の学校の条件として宗教教育に制限をかけた 1899 年の文部省訓令 12 号に圧力を受けたことや、プロテスタント系のキリスト教主義学校等によって、社会的に影響力のある社会階層の者が多くプロテスタントになったのを見ることで（ 井上ほか 2018 p.207） ^17^、カトリックの方も社会の上層部出身にターゲットをシフトさせ、お嬢様学校に白百合や雙葉などが変貌したと、記している。その際考えられたのは、共感者を増やすことが、布教を巧みにする手段となるということであったと想定されている。しかしこの選択肢 7 と Q12G のクロス集計は有意差が出なかった。キリスト教主義学校通学経験者等の当事者たちから、特に「キリスト教への共感者（シンパサイザー）を増やすこと」は支持されているわけではない。

### 3.2 キリスト教主義学校の生徒・学生のイメージ並びに皇室女子のキリスト教主義学校通学の是非、及び愛と奉仕の精神について

次にキリスト教主義学校の生徒・学生のイメージを聞いた Q29 をみてみよう。この問いも MA で「イメージしたもの」3 つと SA で「最もイメージしたもの」1 つを挙げてもらった。「該当なし/答えたくない」を含め 29 個の選択肢を用意したが、その結果は 表3-2-1 のようになる。

**表3-2-1.** Q29「キリスト教主義学校生徒・学生のイメージ」MA・SA.

	合 計	外 国 語 が 得 意 で あ る	話 し 上 手 で あ る	教 養 が 豊 か で あ る	他 人 を 思 い や る 心 が 豊 か で あ る	品 が よ い	品 が 悪 い	社 会 的 活 動 へ の 関 心 が 高 い	国 際 的 な 問 題 へ の 関 心 が 高 い	高 潔 で あ る	卑 劣 で あ る
1. イメージしたもの (3つまで)	700	154	60	141	81	114	8	67	96	18	2
	100.0%	22.0%	8.6%	20.1%	11.6%	16.3%	1.1%	9.6%	13.7%	2.6%	0.3%
2. 最もイメージしたもの (1つだけ)	700	96	18	62	29	54	3	20	34	6	1
	100.0%	13.7%	2.6%	8.9%	4.1%	7.7%	0.4%	2.9%	4.9%	0.9%	0.1%

	裕 福 で あ る	清 貧 で あ る	容 姿 端 麗 で あ る ( み め う る わ し い )	容 姿 端 麗 で は な い ( み め う る わ し く な い )	排 他 的 で あ る	社 交 的 で あ る	お 高 く と ま っ て い る	頭 で っ か ち で あ る	実 行 力 が あ る	親 し み や す い	得 体 が 知 れ な い
1. イメージしたもの (3つまで)	103	5	14	6	12	25	32	6	5	9	30
	14.7%	0.7%	2.0%	0.9%	1.7%	3.6%	4.6%	0.9%	0.7%	1.3%	4.3%
2. 最もイメージしたもの (1つだけ)	51	3	2	0	2	5	12	2	1	4	16
	7.3%	0.4%	0.3%	0.0%	0.3%	0.7%	1.7%	0.3%	0.1%	0.6%	2.3%

	生 活 習 慣 が 規 則 正 し い	論 理 の 整 合 性 を 尊 ぶ	教 え を 忠 実 に 信 じ る	伝 統 志 向 で あ る	先 進 的 で あ る	善 良 で あ る	お 節 介 や き で あ る	該 当 な し \ 答 え た く な い
1. イメージしたもの (3つまで)	25	17	57	33	4	15	4	223
	3.6%	2.4%	8.1%	4.7%	0.6%	2.1%	0.6%	31.9%
2. 最もイメージしたもの(1つだけ)	8	3	31	10	0	2	2	223
	1.1%	0.4%	4.4%	1.4%	0.0%	0.3%	0.3%	31.9%

Q29 で「該当しない/答えたくない」と Q12G をクロス集計すると、 表3-2-2 のようになる（カイ二乗<0.01）。当事者の方が「該当しない/答えたくない」を選ぶ人が多いことは事前に予想された通りだが、実際の通学経験者よりも、緩やかな当事者の方にこの選択肢を選ばない傾向にあることは少し意外であった。その理由を想像するに、一部の通学経験者には、世間的なイメージ、色眼鏡で自分らを眺めること への反撥があるのかもしれないが、そのあたりは今後インタビュー調査を重ねることで明らかにしたい 表3-2-2。

**表3-2-2.** キリスト教主義学校への関与 × Q29 選択肢「該当なし/答えたくない」.

			選択肢 29 (該当なし/答えたくない) を選択していない	選択肢 29 (該当なし/答えたくない) を選択した	
全体		700	477	223	
		100.0	68.1	31.9	
Q12G. あなたのキリスト教主義学校への関与についてお聞きします。当てはまるものを選んでください。	通ったことがある	108	87	21	n
		100.0	80.6	19.4	%
	通ったことはないが、関わったことがある・関りのある人がいる	168	145	23	n
		100.0	86.3	13.7	%
	上記のいずれにも該当しない	424	245	179	n
		100.0	57.8	42.2	%

この Q29 で「該当しない/答えたくない」を除いた選択肢の度数の多い方から並べると 図3-2-2（下部）のようになる。

**図3-2-1.**
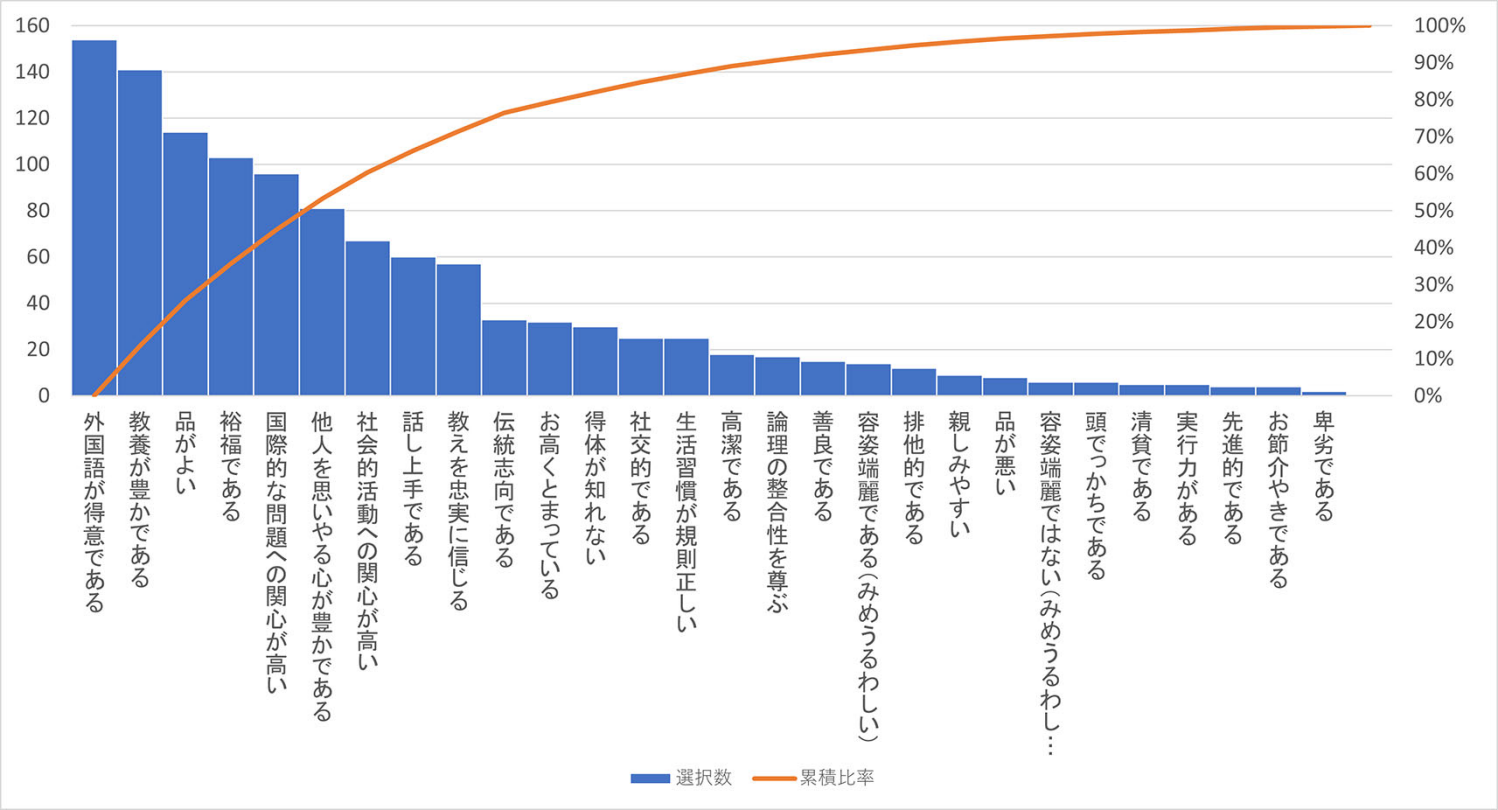
Q29「キリスト教主義学校生徒・学生のイメージ」の選択肢のMAで選ばれた数(度数順).

最も多く回答を集めたのはこの「該当なし/答えたくない」の 31.9% である（MA、SAとも）が、それを除くと MA ベースで実質もっとも多く回答されたのは選択肢 1 の「外国語が得意である」の 22.0% である。キリスト教主義学校はその草創期から外国語教育に力点を置き、宗教教育との間で学内での緊張関係の可能性も胚胎してきた。なお 佐藤 (2006) は 1980 年代に社会全体で立身出世物語が光を失うのに応じてその欲望の対象であったファム・ファタルもその輝きを失ったと指摘し、代わりにキリスト教主義学校がその存在意義を主張する役割を担い始めたのが、外国語教育であると指摘する（ 佐藤 2006 pp.187-189）が、外国語教育への期待は、古くからあったし、今回の調査結果でもまたその期待が裏づけられうる。

次にいま Q29 の選択肢 1 の説明途上ではあるが、クロス集計の都合上、女性皇族のキリスト教主義学校への関与の是非をみた Q11 の単純集計結果を先にみておく。「女性皇族にキリスト教主義学校出身者が複数人いることについて、あなたはどのように評価しますか」という設問に 5 つの選択肢を用意した。選択肢 1「望ましいことだ」6.3%、選択肢 2「どちらかといえば望ましいことだ」15.1%、選択肢 3「どちらともいえない」70.7%、選択肢 4「どちらかといえば望ましくないことだ」4.4%、選択肢 5「望ましくないことだ」3.4%となっている（ 表3-2-3）。

**表3-2-3.** Q11 女性皇族のキリスト教主義学校への関与(単純集計).

選択肢	回答数	割合
望ましいことだ	44	6.3%
どちらかといえば望ましいことだ	106	15.1%
どちらともいえない	495	70.7%
どちらかといえば望ましくないことだ	31	4.4%
望ましくないことだ	24	3.4%
合計	700	100.0%

判断保留を意味する選択肢 3 が非常に多く7 割を超え、次に多いのは選択肢 2 の「どちらかといえば望ましいことだ」である。「どちらともいえない」の 70.7% の人びとがこれを選んだ理由は、自由記入欄を設けていないので不詳だが、望ましい部分と望ましくない部分の双方あるという意味での判断保留の場合、皇族とはいえあくまでも個人の選択の問題ゆえ評価・判断の対象でないという趣旨の場合、さらに「よく分からない」に相当する場合、いずれのケースもあると想定される。ただし望ましい部分と望ましくない部分の双方あるという意味で判断保留する者は一定数いると考えられる。神道と結びついているはずの皇室の一員がキリスト教主義学校に通うことに反撥する層がいるであろうし、他方、キリスト教主義学校で西洋文化の根底にあるキリスト教を学び、国際的に通用する教養と外国語力も身につけることで展開されるであろう皇室外交に期待する意識も強いであろうし、その双方の拮抗する側面が「どちらともいえない」の 70.7% という結果になっているとも考えられる。

なお、この Q11 が本研究において重要である理由は、1.2.1 並びに 1.2.3 で 井上ほか (2018) の文脈に添って説明はした。ただしそこでは愛と奉仕の精神を教育目標にしているがゆえに、皇室女子も、ということを記したにすぎない。　　

そこでここでは 佐藤 (2006) の議論を少しみておく。佐藤は「美智子皇后、雅子皇太子妃が、カトリック 系ミッション・スクールの出身であることは周知のとおりだ。では、ミッション・スクールには、本当に 「身分の高い」方々が在籍していたのだろうか」（ 佐藤 2006 p.67）と問いかけ、第三高等学校と同志社の生徒の出身を華族、士族、平民に分けた明治 33 年のデータと、竹内洋の明治期の高等中学校のデータを比較する。そうすると竹内の示した全国平均の平民率よりも三高の平民率が高く、さらにその三高の平民率より同志社の平民率が高いという。そして当然のことながら華族は学習院に集中しているという。したがって事実としては、やんごとなき方々とキリスト教主義学校生徒とは数的には相関しない。しかしイ メージとしては相関する。その二つを結びつけるのはリスペクタビリティ、ハビトゥスであると佐藤はいう。「近代に入って誕生した日本の華族は、率先してリスペクタブルな者となった。彼らの多くが「洋行し」欧米的教養を身につけたことは当然といえる。逆に華族でなくともその「ハビトゥス」を獲得することで、「リスペクタブル」な者となることは可能だった。ミッション・スクールは、その「プラティーク」を提供する場だったのである」（ 佐藤 2006 p.73)。

また佐藤は美智子上皇妃と雅子皇后を比較して、美智子妃の結婚時は大ブームになったのに雅子妃のとき比較的盛り上がりに欠けたのは、先述のように立身出世物語が衰退したのにあわせてファム・ファタルの輝きが失われたのと同時に、雅子妃が田園調布雙葉（小学校は編入学、小学校中学校卒業され高校生のときボストン移住）のあと、ハーバード、東大（学士入学）、外務省、オックスフォードと、立身出世の男性倫理を体現しているからであろうと述べている（ 佐藤 2006 pp.188-189）。そのことも、この「どちらともいえない」 の多さに表われているのかも知れない。

この 「どちらともいえない」 の問題はそれを問う選択肢を用意する枝問を設けるであろう、今後の調査に詳細は委ねるとして、上述のように 佐藤 (2006 p.67, pp.182-185) でも 井上ほか (2018) でも女性皇族のキリスト教主義学校への通学が、キリスト教主義学校のイメージ形成に強く介在している点を指摘しているので、この Q11 を Q12 につぐ本研究の分析の軸になる変数と考え、次にこれを基にした組変数について記しておく。

この Q11 の選択肢 1，2 で 1 グループとし、選択肢 3 はそのままで、それに選択肢 4 と 5 で 1 グループとする、3 つのカテゴリーに纏めた Q11G を作ることにする。

さて、ここで Q11 による分析の軸になる変数 Q11G の説明を終えたので、ようやく、また本項のテーマである Q29 の方に戻る。この Q29 の MA での選択肢のうち多く選ばれたものから順に、設問にその選択肢を配した趣旨を述べ、必要に応じてキリスト教主義学校当事者指数を示す Q12G 及び、女性皇族のキリスト教主義学校の関与の是非を示す Q11G、それぞれとのクロス集計結果をみていく。

Q29 の単純集計はキリスト教主義学校のイメージをキリスト教主義学校当事者・非当事者あわせた本調査の回答者全体がどのように考えているかを示すものであるのに対して、Q12 のキリスト教主義学校関与度指数とクロス集計することで、特に当事者がそれをどのように捉えているかが分かる。当事者以外によって抱かれたイメージはイマジネール（想像上の>イメージ）という言葉に通じる、「想像されたもの」という意味でのイメージであるのに対して、キリスト教主義学校の通学者・通学経験者のいだくイメージは直接経験の「記憶」「記憶に残った像」という意味でのイメージであるといえる。他方、通学者・通学経験者以外の緩やかな当事者は、「期待」、「印象」レベルのイメージであるので、「イメージ」という言葉の広がりが、クロス集計の結果に反映されるといえる。

そこで Q29 の各選択肢の検討の方、まずは先ほどみた選択肢 1 の方に戻るが、いまみた女性皇族のキリスト教学校への関与の是非についてグループ化した Q11G を独立変数として、この選択肢 1「外国語が得意である」を選んだ人と選ばなかった人を 1，0 にして従属変数としてクロス集計した結果は、以下のようになる（ 表3-2-4）。

**表3-2-4.** Q11G「女性皇族の関与」× Q29 選択肢「外国語が得意」.

		合計	外国語が得意であるを選択していない	外国語が得意であるを選択した
全体		700	546	154
		100.0%	78.0%	22.0%
Q11G. 女性皇族にキリスト主義学校出身者が複数人いることについて、あなたはどのように評価しますか。	望ましい・どちらかといえば望ましい	150	102	48
		100.0%	68.0%	32.0%
	どちらともいえない	495	399	96
		100.0%	80.6%	19.4%
	どちらかといえば望ましくない・望ましくない	55	45	10
		100.0%	81.8%	18.2%

女性皇族のキリスト教主義学校への関与を Q11G で「望ましい」と考える者は、Q29 の選択肢 1 の「外国語が得意である」を選ぶ者が 32.0%、「どちらともいえない」と考える人で「外国語が得意である」を選ぶ者は 19.4%、「望ましくない」を選ぶ人では「外国語が得意である」を選ぶ者は 18.2% であった（カイ二乗<0.01）。つまり女性皇族のキリスト教主義学校への関与を「望ましい」と考える者ほど、キリスト教主義学校の生徒・学生のイメージについて「外国語が得意である」を選ぶ傾向にある。美智子上皇妃や雅子皇后がキリスト教主義学校出身で、また雅子皇后はハーバード大と外務省出身でもあり、国民からの皇室外交への期待も高いことと、この結果は関連して理解できよう。

次にこの選択肢 1 と Q12G をクロス集計すると、以下の 表3-2-5 のようになる。カイ二乗<0.01 であるが、実際のキリスト教主義学校の通学者よりも、緩やかな当事者の方がこの選択肢を多く選ぶ傾向にある。これは外国語が得意であるということが、イメージ先行になっている部分があることを示しているといえよう。Q12 そのものとクロス集計した結果は 表3-2-6（カイ二乗<0.05）のようになっている。特にこの緩やかな当事者のなかのどのカテゴリーがこれを選ぶということはなく、どれも通学経験者よりは多く選んでいる。これはイメージ先行ということもあるが、実際語学に堪能な人は多くても自分はそうではないという場合もあり、そういう場合、実際に通っていても、この選択肢に〇をつけにくいという事情もあると考えられる。

**表3-2-5.** Q12G「キリスト教主義学校関与度」×Q29 選択肢「外国語が得意」.

		合計	外国語が得意であるを選択していない	外国語が得意であるを選択した
全体		700	546	154
		100.0%	78.0%	22.0%
Q12G. あなたのキリスト教主義学校への関与についてお聞きします。当てはまるものを選んでください。	通ったことがある	108	82	26
		100.0%	75.9%	24.1%
	通ったことはないが、関わったことがある・関りのある人がいる	168	116	52
		100.0%	69.0%	31.0%
	上記のいずれにも該当しない	424	348	76
		100.0%	82.1%	17.9%

**表3-2-6.** Q12 キリスト教主義学校への関与(グルーピング前) × Q29 選択肢「外国語が得意」.

		合計	外国語が得意であるを選択していない	外国語が得意であるを選択した
全体		700	546	154
		100.0%	78.0%	22.0%
Q12. あなたのキリスト教主義学校への関与についてお聞きします。当てはまるものを選んでください。	通ったことがある	108	82	26
		100.0%	75.9%	24.1%
	通ったことはないが、受験したことはある	55	39	16
		100.0%	70.9%	29.1%
	受験したことはないが、受験したい学校がある	42	29	13
		100.0%	69.0%	31.0%
	受験候補ではないが、行ってみたい・行ったことがある	23	15	8
		100.0%	65.2%	34.8%
	通ったことはないが、家族に通ったものがいる	48	33	15
		100.0%	68.8%	31.3%
	上記のいずれにも該当しない	424	348	76
		100.0%	82.1%	17.9%

Q29 の MA で次に多かったのは選択肢 3 の「教養が豊かである」であり、MA ベースで 20.1% である。キリスト教主義学校はヨーロッパ文化の根底にあるキリスト教を教育の基盤理念とした学校であるだけに、教養への期待が高いことは納得がゆく。ヨーロッパの名門大学の多くが、修道院の大聖堂の付属施設を源流として発展し、神学部、法学部等の専門課程進学の前提としてリベラルアーツ、自由七学芸を学ぶことを義務づけてきた歴史を踏まえると、これは順当である。

この選択肢 3 を選んだ人と選ばなかった人を 1，0 にして従属変数とし、Q12G のキリスト教主義学校関与度を独立変数としてクロス集計すると 表3-2-7 のようになる。キリスト教主義学校に通学経験のある者は「教養が豊かである」を選ぶ者が 31.5%、通学経験はないが、キリスト教主義学校に当人ないしは家族がかかわりある者で「教養が豊かである」を選ぶ者は 23.2%、かかわりのない人では「教養が豊かである」を選ぶ者は 16.0% であった（カイ二乗<0.01）。キリスト教主義学校にかかわりの高い者ほど、キリスト教主義学校出身者を「教養が豊かである」と自認している、といえよう。ここで「自認」と書いたが、周囲の友人 に対する認識も含めた広い意味での自己認識、「われわれ」認識といえよう。 佐藤 (2006 pp.147–149) はキリスト教主義学校のスクールカラーを端的に示すものとして「リベラル・アーツ」があるとし、リベラル・アーツを標榜し良妻賢母教育を批判した新渡戸稲造の講演を紹介するが ^18^、そういうリベラル・アーツの伝統が現在も引き継がれているといえるであろう。

**表3-2-7.** Q12G「キリスト教主義学校関与度」× Q29 選択肢「教養が豊かである」.

		合計	教養が豊かであるを選択していない	教養が豊かであるを選択した
全体		700	559	141
		100.0%	79.9%	20.1%
Q12G. あなたのキリスト教主義学校への関与についてお聞きします。当てはまるものを選んでください。	通ったことがある	108	74	34
		100.0%	68.5%	31.5%
	通ったことはないが、関わったことがある・関りのある人がいる	168	129	39
		100.0%	76.8%	23.2%
	上記のいずれにも該当しない	424	356	68
		100.0%	84.0%	16.0%

また女性皇族のキリスト教学校への関与の是非について、Q11G を独立変数として、Q29 の選択肢3を選んだ人と選ばなかった人を 1，0 にして従属変数としてクロス集計した結果は、 表3-2-8のようになる。

**表3-2-8.** Q11G 女性皇族の関与 × Q29 選択肢「教養が豊かである」.

		合計	教養が豊かであるを選択していない	教養が豊かであるを選択した
全体		700	559	141
		100.0%	79.9%	20.1%
Q11G. 女性皇族にキリスト主義学校出身者が複数人いることについて、あなたはどのように評価しますか。	望ましい・どちらかといえば望ましい	150	103	47
		100.0%	68.7%	31.3%
	どちらともいえない	495	411	84
		100.0%	83.0%	17.0%
	どちらかといえば望ましくない・望ましくない	55	45	10
		100.0%	81.8%	18.2%

女性皇族のキリスト教主義学校への関与を「望ましい」と考える者は、「教養が豊かである」を選ぶ者が31.3%、「どちらともいえない」と考える人で「教養が豊かである」を選ぶ者は 17.0%、「望ましくない」を選ぶ人では「教養が豊かである」を選ぶ者は 18.2% であった（カイ二乗<0.01）。選択肢 1「外国語が得意である」同様、選択肢 3 についても、キリスト教主義学校出身の女性皇族への期待する人が、外国語や教養などの皇室外交と結びつく能力がキリスト教主義学校の生徒・学生にはあると捉えているといえよう。

Q29 の MA ベースでつぎに多い（実質 3 番）のは選択肢 5 の「品がよい」で 16.3% である。先述の 井上ほか (2018) においても記されていたように、キリスト教主義学校が情操教育をセールスポイントとしているので、この問いの答えが比較的多いのはその点で納得がゆくが、他方、後述する選択肢 4 の「他人を思いやる心が豊かである」の方こそが、 井上ほか (2018) でいわれた先述の奉仕の精神に通じ、キリスト教主義学校の情操教育の成果として第一に妥当すべきであるが、そちらは 11.6% で 29 選択肢中 7 番目（実質 6 番目）と、高い順位ではない。この選択肢 4 の「他人を思いやる心が豊かである」という選択肢とここの 「品がよい」はともに情操教育の成果ではあり、相互に密接に関連はするが、日本語の語感として「品が良い」はより外側に滲み出るもの、「他人を思いやる心が豊かである」はより内面的なものといえる。本調査では、キリスト教主義学校在校生卒業生の階層再生産戦略 ^19^について知ろうとしていて（その面のみに関していうと本研究の調査結果はあまり成功していない可能性があり、そこは別稿で論じるが）、階層再生産戦略に結びつくのは「品の良さ」の方であるともいえる。この選択肢 5 と Q11G をクロス集計したが有意差はなかった（ 表3-2-9）。

**表3-2-9.** Q11G 女性皇族の関与 × Q29 選択肢「品が良い」.

		合計	品が良いを選択していない	品が良いを選択した
全体		700	586	114
		100.0%	83.7%	16.3%
Q11G. 女性皇族にキリスト主義学校出身者が複数人いることについて、あなたはどのように評価しますか。	望ましい・どちらかといえば望ましい	150	119	31
		100.0%	79.3%	20.7%
	どちらともいえない	495	422	73
		100.0%	85.3%	14.7%
	どちらかといえば望ましくない・望ましくない	55	45	10
		100.0%	81.8%	18.2%

一方 Q12G とクロス集計するとカイ二乗<0.01 で有意差があった（ 表3-2-10）。キリスト教主義学校関与度の高い者ほど、「品が良い」を選択する傾向にある。

**表3-2-10.** Q12G キリスト教主義学校関与度 × Q29 選択肢「品が良い」.

		合計	品が良いを選択していない	品が良いを選択した
全体		700	586	114
		100.0%	83.7%	16.3%
Q12G. あなたのキリスト教主義学校への関与についてお聞きします。当てはまるものを選んでください。	通ったことがある	108	82	26
		100.0%	75.9%	24.1%
	通ったことはないが、関わったことがある・関りのある人がいる	168	134	34
		100.0%	79.8%	20.2%
	上記のいずれにも該当しない	424	370	54
		100.0%	87.3%	12.7%

Q29 の MA ベースでつぎに多い（実質 4 番）のは選択肢 11「裕福である」で 14.7% である。1.2.3 で先述のように、 井上ほか (2018) の先行研究ではキリスト教主義大学の女子がキリスト、きれい、金持ちの 3K であるといわれている点を強調するが、この本の第1章で論証もなくいわれるこの話のうち、きれいはひとまず措いて金持ちについては世間一般のイメージにおいても妥当することが、ここからうかがわれる。これについては 井上ほか (2018) 同様、 佐藤 (2006) も「京童が口にする三 K「かわいい、金持ち、キリスト教」あるいは「きれい、かしこい、金持ち」の、いずれのヴァージョンにも登場する「金持ち」という要素だ」（ 佐藤 2006 p.74）と記す。これについても佐藤は授業料でキリスト教主義学校が他より高いことはなく、最初期に進出した私立学校の多くがキリスト教主義学校であったことに金持ちイメージの理由があり、さらには「 「羨望」の対象であった欧米文化あるいは都市文化というミッション・スクールの「徴」が、直接経済スケールに反映するものとして語られてきたという側面も無視できない」（ 佐藤 2006 p.80）と分析している。

なおこれと Q11 の女性皇族のキリスト教主義学校の是非の問い及び Q12 のキリスト教主義学校関与度とクロス集計したが、いずれも有意差はなかった。

我々のインタビュー調査で小中高とプロテスタントの女子校（小学校の際は若干男子生徒もいた）に通い公立大学に通っている（調査日当時）B さんは、次のように語っている（調査日 2019 年 1 月 22 日）。なお、B さんの調査に先立ち筑波大学図書館情報メディア系倫理審査委員会より「科研費挑戦的萌芽研究「キリスト教主義学校から見る寛容と洋化―ステークホルダーらの期待と文化資本」（2018~2019年度（代表後藤嘉宏））初年次ウェブモニター調査のためのプレ調査」（通知番号第 30-84 号）の承認を得ている（以下、B さんの発言の引用の際は、この倫理審査についての記述を重複を避けるため控える）。

後藤:キリスト教主義学校に行っていることで外の人の目が、例えばお母さまが職場の同僚に「うちの娘○○（学校名）で」って言ったときに「なかなかハイカラね」とか、そういう目で見られたという感じはどうですか?

B さん:例えば、ここの大学入ったときに、自分の学校を説明する上で、女子高で私立でキリスト教というと、かなりお嬢様みたいなイメージを持たれたりするんですけど。でも私自身全くそんな意識はないし、何ならキリスト教、みんなが学んでいないことを学んでるという特別感を私的には感じたりしていたので。

後藤:お嬢様という感じは公立大入ったときの周囲の目で……

B さん:結構あったと思います。

要は現実よりもイメージ先行ということになる。

Q29 の MA ベースでつぎに多い（実質 5 番）選択肢は 8 の「国際的な問題への関心が高い」で 13.7% である。これは「該当なし・答えたくない」を除き実質 1 位の、先にみた「外国語が得意である」とも関連する選択肢である。キリスト教主義学校はキリスト教という西洋文化の根底を理解させることで、外国語と国際感覚を学ぶ場を提供しているという趣旨でこの設問を設けた。ただしこれを Q12G とクロス集計したが、有意差はなかった。キリスト教主義学校当事者において「国際的な問題への関心が高い」という自己認識は非当事者よりも多くも少なくもない。さらに Q11G ともクロス集計したが、これも有意差はなかった。女性皇族のキリスト教主義学校通学歴を是とする人は皇室外交への期待も大きいと予想し、それに関連する選択肢 1 の「外国語が得意である」については有意差がありそのことは裏づけられたが、それとともにその選択肢1と強く関連する「国際的な問題への関心が高い」の方については有意差がなかった。こちらは予想外の結果である。ただし皇室外交は憲法学的見地から政治的発言が制約されていて、そのことに鑑みると、皇室外交に求められるのは問題解決能力よりはコミュニケーション力である。その意味では、「国際的な問題への関心が高い」については有意差がなく「外国語が得意である」については有意差があることは制度上の面からすると一応納得がゆく。

1.2.2 で先述のように 井上ほか (2018) で、女子アナにキリスト教主義学校出身率が高いのは、愛と奉仕の精神を育むとそれらの学校のウェブページに記載されていることに照応する、と書かれている。しかしウェブで謳われていることと実際は必ずしも一致しないし、教育目標通りの効果もあるとは限らないが、 井上ほか (2018) は調べていない。したがって Q29 の選択肢 4 として「他人を思いやる心が豊かである」を入れた。

4 段落前に先述したようにこの選択肢 4 は MA ベースでは全体で 11.6% で 29 選択肢中実質 6 番目で、高い順位ともいえない。しかしキリスト教主義学校関与度指数を示す Q12G とクロス集計するとカイ二乗<0.01で有意差が出た（ 表3-2-11）。

**表3-2-11.** Q12G キリスト教主義学校関与度 × Q29 選択肢「他人を思いやる心」.

		合計	他人を思いやる心が豊かであるを選択していない	他人を思いやる心が豊かであるを選択した
全体		700	619	81
		100.0%	88.4%	11.6%
Q12G. あなたのキリスト教主義学校への関与についてお聞きします。当てはまるものを選んでください。	通ったことがある	108	92	16
		100.0%	85.2%	14.8%
	通ったことはないが、関わったことがある・関りのある人がいる	168	139	29
		100.0%	82.7%	17.3%
	上記のいずれにも該当しない	424	388	36
		100.0%	91.5%	8.5%

しかしこれには我々の事前の予想とは違う部分があった。キリスト教主義学校に全く関与しない者がこの答えを選ばない傾向にあることは予想の通りで、そのカテゴリーでこれを選んだ者は 8.5% であった。他方、キリスト教主義学校に実際に通った・通っている者でこの選択肢を選んだのは 14.8% であり、その他の関与者の17.3%よりもやや低い数値であった。

そこで Q12 をグループ化せずに集計したところ 表3-2-12 のようになり、各セルの実数は少ないので有意差は出にくいものの、カイ二乗<0.05で有意差を確認できた。これを見ると、キリスト教主義学校 に 「通ったことはないが、受験したい学校がある」者のみが、「他人を思いやる心が豊かである」を選択していることが分かる。これは「他人を思いやる心」を涵養させるだろうという受験前の期待が、キリスト教主義学校の生徒イメージとして数字に現れている面があると考察・想定される。他方、実際に通学してみると、キリスト教主義学校に入学する課程の、前の課程の学校のクラスメイトとさほど変わらなかったとか、あるいは自分自身をふり返ってみて他人を思いやっていないと考えると自省するとか等の諸事情が想定され、それは家族に通学者がいる者についても同様と推察される。

**表3-2-12.** Q12 キリスト教主義学校関与度(グルーピング前) × Q29 選択肢「他人を思いやる心」.

		合計	他人を思いやる心が豊かであるを選択していない	他人を思いやる心が豊かであるを選択した
全体		700	619	81
		100.0%	88.4%	11.6%
Q12. あなたのキリスト教主義学校への関与についてお聞きします。当てはまるものを選んでください。	通ったことがある	108	92	16
		100.0%	85.2%	14.8%
	通ったことはないが、受験したことはある	55	47	8
		100.0%	85.5%	14.5%
	受験したことはないが、受験したい学校がある	42	31	11
		100.0%	73.8%	26.2%
	受験候補ではないが、行ってみたい・行ったことがある	23	20	3
		100.0%	87.0%	13.0%
	通ったことはないが、家族に通ったものがいる	48	41	7
		100.0%	85.4%	14.6%
	上記のいずれにも該当しない	424	388	36
		100.0%	91.5%	8.5%

このあたりについてはキリスト教主義学校の学生や卒業生に今後質的調査をすることで確認をしていきたい。

なお、この選択肢 4 と Q11G とクロス集計するとカイ二乗<0.01 で有意差が出た（ 表3-2-13）。

**表3-2-13.** Q11G 皇族女子の関与 × Q29 選択肢「他人を思いやる心」.

		合計	他人を思いやる心が豊かであるを選択していない	他人を思いやる心が豊かであるを選択した
全体		700	619	81
		100.0%	88.4%	11.6%
Q11G. 女性皇族にキリスト主義学校出身者が複数人いることについて、あなたはどのように評価しますか。	望ましい・どちらかといえば望ましい	150	115	35
		100.0%	76.7%	23.3%
	どちらともいえない	495	456	39
		100.0%	92.1%	7.9%
	どちらかといえば望ましくない・望ましくない	55	48	7
		100.0%	87.3%	12.7%

皇室女子とキリスト教主義学校とを結びつける者ほど、キリスト教主義学校での思いやり教育に期待していることが分かる。これは本稿 1.2.1 で述べた、先行研究の④「皇族女子のキリスト教主義学校入学が多いのも、愛と奉仕の精神を育むとの期待があるからである」を、一般の人びとの意識の側から裏づける結果であるといえる。

なお、ここまでのクロス集計結果は通学経験の有無よりは思いやりがイメージ先行であるというもので、それとはある意味、矛盾もしうるが、幼稚園から大学院修士課程まで一貫して同じ系列のキリスト教主義学校に通っていた A さんを我々はインタビュー調査したが、そこでは情操教育とキャリア形成について直接結びつけるような、次のような発言が得られている（調査日 2019 年 5 月 31 日）。

「情操教育というところで、まず、○○学院の校訓の「人になれ、奉仕せよ」がひとつ、大きなものであって。小学校のときにも教訓というか、礼拝のときに毎朝暗唱するものがありまして、「自分に厳しく人に優しく」というのと「強く鍛える体と心」とか。あと「艱難に耐え、常に祈りなさい」っていう、そういうものを礼拝の前にみんなで言うところがありまして。ちょっとその「人になれ、奉仕せよ」も先生方が口々におっしゃるんですけど、私一回、結局どういう意味なんですかね、って小学校の時に訊いたら、先生たちも自分なりの考えはあるけど、正解はないんだということで、人生の中で見つけていってごらん、ということで、それを頭の中に置きながら普段生活しているところがあるんですけども。

それで、キャリアとかを考えた時に、私の大学の学部選びでも大きく関与しているところが、実はありまして、高校生の時に火事現場に遭遇したことがありまして、家におばあさんがいたんですけど、そこで入って助けたことがありまして、消防署で表彰をもらったときに、私の中での行動原理が、ついつい手を出してしまうみたいなところがありまして、それもあって、何かしらの人の助けとか役に立ちたいなというところから福祉の道を選びたいということで、大学選びで福祉を専攻したというところが私の中で大きなターニングポイントで、自分の中のキャリア形成じゃないですけれども、大きく土台としてあるものなんじゃないかと。道徳とか情操教育みたいなところを考えると、**の中では「人になれ、奉仕せよ」とかキリスト教の校訓みたいなところはみんなの中に、何年間もいる中で根底に根付いてくるものなんじゃないかと」 （**は録音音声不明瞭な箇所）。

この発言の「キリスト教の校訓みたいなところはみんなの中に、何年間もいる中で根底に根付いてくるものなんじゃないかと」いうことからも、附属・系列校を通じて長くその学院・学園に在籍することで、思いやりの心、奉仕の精神は培われるものと考えられる。他方、ほとんどすべての法人で定員的には大学のみが突出して多いので、附属・系列校からいる人に比して相対的に大学の四年間は短期間ゆえ、大学生ではこれらが定着しない人の方が比率的には多いことも、この発言は暗に示しうる。

また小学校から高校まで同じ法人のプロテスタントの学校に通い、大学で公立大学の看護学科に進学した B さんに「聖書教育を受けてボランティアへの意識が高まったなどの、なんらかの影響はありましたか?」と聞いたところ、「小学校のときから、たとえばなしでキリストがゆるすお話を教えられてきたので、人に優しくあろうという感じの観念は小学校からみんなに対して伝えられてたのかなあと思います」と答えている（調査日 2019 年 1 月 22 日）。

Q29 の選択肢で MA ベースでつぎの 7 番目だったのは、選択肢 7 の「社会的活動への関心」で 9.6% である。これも選択肢 8 の「国際的な問題への関心が高い」同様、Q12G 並びに Q11G とクロス集計したが、いずれも有意差はなかった。宗派問わず宗教人の多くがボランティア活動に熱心で、特にキリスト教はボランティア活動に積極的である印象が強いので、この選択肢自体がそこそこ選ばれたことは納得がゆくが、その反面、キリスト教主義学校当事者たちから特にこの選択肢が多く選ばれている訳ではないことからすると、このことはイメージ先行である可能性があるし、また「社会的活動」というワーディングが政治経済活動全般に及ぶ可能性があり、社会奉仕と必ずしも回答者の認識で直結しなかった可能性も、否めない。

Q29 の選択肢で MA ベースでつぎの8番目だったのは、選択肢 2 の「話し上手である」で 8.6% である。コミュニケーション力の高さという点で、選択肢 3 の「教養が豊かである」とも結びつく内容といえる。

この選択肢と Q11G をクロス集計すると 表3-2-14 のようになる。

**表3-2-14.** Q11G 皇族女子の関与 × Q29 選択肢「話し上手である」.

		合計	話し上手であるを選択していない	話し上手であるを選択した
全体		700	640	60
		100.0%	91.4%	8.6%
Q11G. 女性皇族にキリスト主義学校出身者が複数人いることについて、あなたはどのように評価しますか。	望ましい・どちらかといえば望ましい	150	122	28
		100.0%	81.3%	18.7%
	どちらともいえない	495	473	22
		100.0%	95.6%	4.4%
	どちらかといえば望ましくない・望ましくない	55	45	10
		100.0%	81.8%	18.2%

カイ二乗<0.01 ではあるが、女性皇族のキリスト教主義学校出身者がいることを「望ましい」と思う人たちと「望ましくない」と思う人たちにこの「話し上手である」という選択肢を選ぶ傾向にあり、「どちらともいえない」という多数派の人たちはこの選択肢を選ばない傾向にある。「望ましい」と思う人たちがこの選択肢を多く選ぶことは、コミュニケーション力という点からの皇室外交への期待という面からも容易に想像できることであり、ある意味予想どおりであったが、「望ましくない」と思う人にもほぼ同程度これを選ぶ者がいることは意外であったが、この理由については今後つめていきたい。

次にこれと Q12G をクロス集計した結果は 表3-2-15 の通りだが、これもカイ二乗<0.01 である。

**表3-2-15.** Q12G キリスト教主義学校関与度 × Q29 選択肢「話し上手である」.

		合計	話し上手であるを選択していない	話し上手であるを選択した
全体		700	640	60
		100.0%	91.4%	8.6%
Q12G. あなたのキリスト教主義学校への関与についてお聞きします。当てはまるものを選んでください。	通ったことがある	108	99	9
		100.0%	91.7%	8.3%
	通ったことはないが、関わったことがある・関りのある人がいる	168	140	28
		100.0%	83.3%	16.7%
	上記のいずれにも該当しない	424	401	23
		100.0%	94.6%	5.4%

当事者（通ったことがある者）よりは緩やかな当事者の方がこの「話し上手である」を選ぶ傾向があり、当事者は非当事者よりは多いが、さほどそれとの差は大きくはない。

よって度数が少ないので検定はしていないが、Q12 をグルーピングしない形でのクロス集計も示しておく（ 表3-2-16）。

**表3-2-16.** Q12 キリスト教主義学校関与度(グルーピング前) × Q29 選択肢「話し上手である」.

		合計	話し上手であるを選択していない	話し上手であるを選択した
全体		700	640	60
		100.0%	91.4%	8.6%
Q12. あなたのキリスト教主義学校への関与についてお聞きします。当てはまるものを選んでください。	通ったことがある	108	99	9
		100.0%	91.7%	8.3%
	通ったことはないが、受験したことはある	55	43	12
		100.0%	78.2%	21.8%
	受験したことはないが、受験したい学校がある	42	35	7
		100.0%	83.3%	16.7%
	受験候補ではないが、行ってみたい・行ったことがある	23	17	6
		100.0%	73.9%	26.1%
	通ったことはないが、家族に通ったものがいる	48	45	3
		100.0%	93.8%	6.3%
	上記のいずれにも該当しない	424	401	23
		100.0%	94.6%	5.4%

「通ったことがある」者以上に「上記いずれにも該当しない」者に近いのは、「通ったことはないが、家族に通ったものがいる」者である。要は当人や家族が通った者よりは、そうではなく受験しただけや受験したいだけ行ってみたいだけの者の方が、話し上手であるというイメージをもっている。現実よりもイメージ先行ということであろう。 井上ほか (2018) の、女子アナにキリスト教主義学校出身が多いという記述があることは、1.2.2 で既出であるが、そういうイメージが共有されていれば「話し上手である」になるが、アナウンサーになれるのはどこの大学であろうとほんの一握りの学生であって、実際に通った当事者にとっては現実とは多少違うと感じられても不思議ではない。つまりアナウンサーになった人の集合で考えると、 井上ほか (2018) でいうように、女子アナについてはキリスト教主義学校出身が多いとして、そのイメージは一般の人びとから共有されていることが、この結果からも分かる。他方、キリスト教主義学校の生徒・学生からすると、そのなかでアナウンサーになれるのはほんの一部で、周囲ではそのような人は見たことないという可能性も高い。

Q29 の選択肢で MA ベースでつぎの 9 番目だったのは、選択肢 24 の「教えを忠実に信じる」で 8.1% である。ただしこの Q29 の選択肢 24 については「教えを忠実に信じる」ということで、信仰心が篤いとも受け取れられるし、批判力の欠如や盲信、ある意味洗脳されているというようなニュアンスでも受け取られ、ややワーディングが曖昧であった。そのことが 18 選択肢中、9 番目という多いとも少ないともいえない順位に表われているのかもしれない。これは Q12G とクロス集計してみたが、有意差はなかった。

つぎに Q29 の選択肢で MA ベースでの単純集計でも回答者の少なかったものについて論じていく。

1.2.3 で先述のように、 佐藤 (2006 p.74) や 井上ほか (2018) はキリスト教、きれい、金持ちという 3K のレッテルがキリスト教主義学校女子に張られているという。そのうちキリスト教は、キリスト教主義学校という言葉の反復になるので措くとする。金持ちについてはすでに選択肢 11 の「裕福である」のところで述べた通りである。他方、きれいについては選択肢 13 で「容姿端麗である（みめうるわしい） 」を用意した。しかしこれは MA で 2.0%、SA で 0.3% しか選ぶ者がいなかった。MA ベースで 18 番目という低い順位である。またキリスト教主義学校当事者指数をグループ化した Q12G とも、女性皇族のキリスト教主義学校通学の是非を示す Q11G ともクロス集計してみたが、いずれも有意差はなかった。「容姿端麗である」という日本語がやや難しいので「みめうるわしい」という補足説明を括弧書きでつけたが、いずれも難しいということもあったかもしれない。また先述のように Q29 はキリスト教主義学校の男子も女子も含めたイメージを聞いているということの影響も大きいと思われる。しかし 井上ほか (2018) にしても 佐藤 (2006) にしてもキリスト教主義学校に男子学生が相当数いることは重々承知の上で、女性バイアスをあえて自分らの研究にかけてキリスト教主義学校を眺めているのであるから、そもそも当人の意識にバイアスのある回答者にとっては、男女合わせた聞き方を今回のようにあえてしてみても著しく結果が変わる訳ではないことも想定される。この数値は以上のワーディングの問題や女子に限定しない聞き方という問題を措いても、極めて低い数値であるといえる。したがって 佐藤 (2006)、 井上ほか (2018) にある女性バイアス、キリスト教主義学校女子が「きれい」だという固定観念が、少なくとも本研究の調査対象とする若い世代では全くないことが分かった ^20^といえよう。もっとも今回調査対象者を東京・神奈川に絞ったが、 井上ほか (2018) も 佐藤 (2006) も京都の事例を基にこの 3K について取り上げているので、関西の府県に調査対象者を求めたら、同じ世代であっても結果は大いに変わった可能性のあることは否めない。

先ほど言及した、 佐藤 (2006) のいう、立身出世のモチベーションがなくなったため、ファム・ファタルが欲望の対象でなくなったとの発言も、女性バイアスがほぼ過去のものになったことを示している。佐藤の著書は 2006 年ではあるが、佐藤の著書の述べた対象そのものは概ね 1980 年代の事象であり、そこから30 年以上経ていて、ファム・ファタルに通じる女性の容姿についての意識はそのとき以上に弱まっており、なおかつ今回の調査票のこの設問が男女問わないイメージを聞いたものであるので、なおのこと容姿についての選択肢が選ばれなかったと考えられる。

他に Q29 の選択肢で MA ベースでも回答者の少なかったという点で特記すべきものとしては、選択肢 23 の「論理の整合性を尊ぶ」で、16 番目で MA ベースで 2.4% しか選ばれていない。これは丸山真男の『日本の思想』でキリスト教とマルクス主義が首尾一貫性を重んじる思想であるがゆえに、無限抱擁的に内外の思想宗教を受け入れてきた日本の固有の文化となじまずに、日本ではこれらキリスト教徒やマルクス主義者が知識人層以外に増えなかったという指摘を意識して入れた選択肢である。

なお、この選択肢を選んだ者と Q12G をクロス集計したが有意差はなかった。実際この回答が少なかったのは、キリスト教の一神教としての首尾一貫性という丸山その他から従来いわれてきた通説を人びとが意識しない結果なのか、あるいはクリスマスやキリスト教式結婚式と類似した習俗のみの次元でキリスト教主義学校の生徒・学生を捉えた結果なのか、この問いだけからは分からない。そもそもこの選択肢を含め多くの選択肢は、この調査における Q29 と類似した別の設問（次段落以降に記すように、Q26 は日本のキリスト教徒について同じ選択肢で聞き、Q27 は日本のキリスト教会の聖職者について同じ選択肢で聞いている）の選択肢と合わせて用意されている。キリスト教主義学校の生徒・学生とキリスト教徒とキリスト教聖職者、これら三者それぞれは関連するが別のものとして考える必要があるからである。

そこでいまふれた Q26 であるが、これは「あなたは日本のキリスト教徒についてどのようなイメージを持っていますか。当てはまるものを上位 3 つまでの間で選び、中でも最もイメージしたものを 1 つ選んでください」という設問で、Q29 と同じ選択肢を配している。

しかしここでも選択肢 23 の「論理の整合性を尊ぶ」は MA ベースで 1.4% しか選ばれていない。生徒・学生に対するイメージの Q29 以上に低い数値である。

また Q27 は「あなたは日本のキリスト教会の聖職者についてどのようなイメージを持っていますか。当てはまるものを上位3つまでの間で選び、中でも最もイメージしたものを 1 つ選んでください」という設問であるが、ここでも Q29 と同一の選択肢を配している。この設問においても選択肢 23 の「論理の整合性を尊ぶ」は MA ベースで 1.9% しか選ばれていない。

キリスト教主義学校の生徒についてのイメージのみであれば、クリスマスやキリスト教式結婚式と同一の習俗、表層の次元で、キリスト教主義学校が捉えられうるから低い数値であるという説明も可能であろうが（リスペクタビリティの説明やトリプルな信仰についての箇所で先に申し上げたように、表層的であることが、精神性や宗教性に必ずしも通じない訳でもないが）、クリスチャンや聖職者のイメージについてもほぼ同様の数値しか得られていないことからすると、キリスト教というもの自体が、現代の若年層からは、そういう丸山のいうような、論理の首尾一貫性とは切り離されて捉えられている可能性が示唆される。

3.3 キリスト教式結婚式の是非及びクリスマスの過ごし方と、キリスト教主義学校関与度指数

井上ほか (2018) はクリスマスやキリスト教式結婚式の隆盛と、キリスト教主義学校の人気とを相同性のあるものとして関連づけるし、濱田の歴史研究でもキリスト教式結婚式とキリスト教主義学校の関連性は示されている。そこでまず本研究でキリスト教式結婚式について尋ねた設問についてみてみる。

Q22. で「キリスト教徒ではない日本人のカップルが、教会で結婚式を挙げることについて、あなたはどのように思いますか」と SA で聞いて、 表3-3-1 のような結果を得ている。

**表3-3-1.** Q22 キリスト教式結婚式の是非.

選択肢	回答数	割合
好ましい	252	36.0%
どちらともいえない	411	58.7%
好ましくない	37	5.3%
合計	700	100.0%

「どちらともいえない」が 58.7% で最も多いが、「好ましい」が 36.0% で「好ましくない」の 5.3% よりかなり多い。全体として判断に迷う人が多いが、それにもかかわらず、肯定的な判断の方に傾いているといえる。なお、『 「現代日本における宗教教育の実証的研究」(1998~1999) 報告書』では、1997 年度の調査から 1999 年度まで「次の事柄について、あなたの同意できる意見にすべて〇をしてください」という問いの 3. として「クリスチャンでない人が、キリスト教会で結婚式をあげるのはおかしい」という項目を設けているが、この結果について同報告書では日韓比較の箇所で一部ふれられるのみである。なおこの「現代日本における宗教教育の実証的研究」のこの設問では 1. そう思う　2. どちらかといえばそう思う　3. どちらかといえばそう思わない　4. そう思わない　の4択であるが、ここの報告での記述は、「そう思う」「そう思わない」の数値のみ示して以下の文章のように日韓比較をしている。「 「そう思う」(+ +) は、日本は 8.2%、韓国は 16.6% で、やはりクリスチャン人口の多い韓国の方が習俗としてのキリスト教式結婚式に批判的なようだが、「そうは思わない」(- -)を見てみると、日本は 48.6% 、韓国は 45.2% で、それほど大きな差はなく、韓国においても、結婚式がイベント化している様子がうかがえよう」（ 井上ほか 2000 p.125）。

本研究の Q22 を Q12G とクロス集計すると 表3-3-2 のようになる。

**表3-3-2.** Q12G キリスト教主義学校関与度 × Q22 キリスト教式結婚式の是非.

		合計	好ましい	どちらとも言えない	好ましくない
全体		700	252	411	37
		100.0	36.0%	58.7%	5.3%
Q12G. あなたのキリスト教主義学校への関与についてお聞きします。当てはまるものを選んでください。	通ったことがある	108	56	49	3
		100.0%	51.9%	45.4%	2.8%
	通ったことはないが、関わったことがある・関りのある人がいる	168	76	83	9
		100.0%	45.2%	49.4%	5.4%
	上記のいずれにも該当しない	424	120	279	25
		100.0%	28.3%	65.8%	5.9%

キリスト教主義学校の当事者ほど、キリスト教式結婚式に肯定的であることが、この表から分かる（カイ二乗<0.01）。

このことからもキリスト教主義学校とキリスト教式結婚式との相同性をいう 井上ほか (2018) の指摘の妥当性が裏づけられる。

またこの結果は先述の 濱田 (2001)とも照合する。なお『 「現代日本における宗教教育の実証的研究」(1998~1999) 報告書』ではこの設問について高校や大学の宗派別の分析を載せていないので、単純には比較できないが、先に引いたように「 「そう思う」(++) は、日本は 8.2%、韓国は 16.6% で、やはりクリスチャン人口の多い韓国の方が習俗としてのキリスト教式結婚式に批判的なようだ」とキリスト教徒がキリスト教式結婚式に批判的であるとの見立てを示しているが、今回の調査結果はむしろキリスト教主義学校とキリスト教式結婚式との親和性が示唆されている。

つぎにクリスマスの過ごし方についてであるが、Q4 で「あなたが生まれ育ったご家族（原家族）あるいはそれに相当する環境では、クリスマスに何をしていましたか。以下の中から、1度でも行ったものを全て選んでください」という設問をした。単純集計の結果は 表3-3-3 の通りである。なお今回の調査のクリスマスについての設問は比較的一般の質問文であったので、必ずしもクロス集計に向かないと考えられるし、キリスト教主義学校当事者指数と相関しそうな選択肢は軒並み少なくしか選ばれなかったが、度数が少ないながらもある程度有意差があるものもあったので、本稿では一応それらのクロス集計結果を示す。

**表3-3-3.** Q4「原家族におけるクリスマスの過ごし方」.

番号	選択肢	回答数	割合
1	ケーキを食べる	606	86.6%
2	プレゼントをあげたり貰ったりする(サンタ・クロースからのプレゼントも含める)	507	72.4%
3	チキン(鶏肉)を食べる	435	62.1%
4	ターキー(七面鳥)を食べる	93	13.3%
5	聖書をみんなで読む	17	2.4%
6	クリスマスツリーを飾る	422	60.3%
7	お祈りをする	22	3.1%
8	みんなでメリークリスマスと言い合う	269	38.4%
9	聖歌や賛美歌を歌う(いわゆるクリスマスソングの1つとして歌うものは除く)	22	3.1%
10	クリスマスソングを歌う	159	22.7%
11	家族の誰かがサンタ・クロースの格好をする	51	7.3%
12	教会に行く	21	3.0%
13	家族以外の者を家に招き、家族と一緒にお祝いをする、あるいは知人の家に家族ぐるみで招かれる	38	5.4%
14	家族では、クリスマスのお祝いは一切しなかった	24	3.4%
15	答えたくない	19	2.7%
	N=	700	100.0%

選択肢 5「クリスマスに聖書をみんなで読んでいましたか」については「読んだ人」「読まなかった人」で 1、0 で集計すると 表3-3-4のようになる。緩やかな当事者に「聖書をみんなで読む」人が多いが、そもそも実数が少ないが、カイ二乗<0.05 で有意差はあった。

**表3-3-4.** Q12G キリスト教主義学校関与度 × Q4 選択肢「クリスマスに聖書を読む」.

		合計	聖書をみんなで読むを選択していない	聖書をみんなで読むを選択した
全体		700	683	17
		100.0%	97.6%	2.4%
Q12G. あなたのキリスト教主義学校への関与についてお聞きします。当てはまるものを選んでください。	通ったことがある	108	104	4
		100.0%	96.3%	3.7%
	通ったことはないが、関わったことがある・関りのある人がいる	168	160	8
		100.0%	95.2%	4.8%
	上記のいずれにも該当しない	424	419	5
		100.0%	98.8%	1.2%

選択肢 7「クリスマスにお祈りをしましたか」について同様の作業をした（ 表3-3-5）。これも実数が少ないもののキリスト教主義学校当事者指数が高いほど、クリスマスにお祈りをしている。これもカイ二乗<0.05 で有意差はある。

**表3-3-5.** Q12G キリスト教主義学校関与度 × Q4 選択肢「クリスマスにお祈りをする」.

		合計	お祈りをするを選択していない	お祈りをするを選択した
全体		700	678	22
		100.0%	96.9%	3.1%
Q12G. あなたのキリスト教主義学校への関与についてお聞きします。当てはまるものを選んでください。	通ったことがある	108	101	7
		100.0%	93.5%	6.5%
	通ったことはないが、関わったことがある・関りのある人がいる	168	161	7
		100.0%	95.8%	4.2%
	上記のいずれにも該当しない	424	416	8
		100.0%	98.1%	1.9%

選択肢 9「クリスマスに聖歌や賛美歌を歌いますか」についても 表3-3-6 のように同様である。これはカイ二乗<0.01 で有意差がある。

**表3-3-6.** Q12G キリスト教主義学校関与度 × Q4 選択肢「クリスマスに聖歌等を歌う」.

		合計	聖歌や賛美歌を歌うを選択していない	聖歌や賛美歌を歌うを選択した
全体		700	678	22
		100.0%	96.9%	3.1%
Q12G. あなたのキリスト教主義学校への関与についてお聞きします。当てはまるものを選んでください。	通ったことがある	108	101	7
		100.0%	93.5%	6.5%
	通ったことはないが、関わったことがある・関りのある人がいる	168	159	9
		100.0%	94.6%	5.4%
	上記のいずれにも該当しない	424	418	6
		100.0%	98.6%	1.4%

我々の質的調査において幼稚園から大学院修士課程まで一貫して同じ系列のキリスト教主義学校に在籍していたAさんは（インタビュー時、修士 2 年）、仏教徒の家庭に生まれ、キリスト教に入信はしていないが、賛美歌のメロディや歌詞からキリスト教への憧憬を強めたと語っている。「キリスト教になじみがあったのかなというところでは。それで他大学行って、中高で聖書・讃美歌やって「もういいや」ってなって他大学、全然宗教関係ない大学行った人たちも、やっぱり会うと、ちょっと礼拝が恋しくなるみたいなところは聞いていて。そういうアイデンティティじゃないですけど、帰属意識みたいなのはあるかな、と。未だに大学別のところ行っちゃった人、高校の**とか、会うと聖句でこれ覚えてるとか言ってみんなで暗唱したりして。・・・親友の人たちは特に、好きな讃美歌は何番かとかで盛り上がってる感じで」（**は音声不鮮明で録音の聴き取り不能箇所。多分「同窓会」。・・・は中略箇所） （調査日 2019 年 5 月 31 日）。一部の在籍生はクリスマスに限定されない日常生活の中に「聖歌や賛美歌を歌う」行動は定着していることが、この結果に示されているのかも知れない。

選択肢 12 の「クリスマスに教会に行きますか」については今まで見てきた選択肢の中でも特にキリスト教主義学校通学者に著しいという形になっている（ 表3-3-7）。この p 値は 0.00031 なので、ほぼ確実にいえることであるといえる。ただ Q4 の質問文は「あなたが生まれ育ったご家族（原家族）あるいはそれに相当する環境では・・・」となってはいるものの、「以下の中から、1 度でも行ったものを全て選んでください」となっていることに引きつられて、キリスト教主義学校に通学していた時代に、学校行事として行ったものにも〇をつけた可能性のあることは否定できない。またクリスマスに限らず教会に行くことを在学生に強く推奨する場合もあることは、我々もインタビューを通じて、聞いてもいる。

**表3-3-7.** Q12G キリスト教主義学校関与度 × Q4 選択肢「クリスマスに教会に行く」.

		合計	教会に行くを選択していない	教会に行くを選択した
全体		700	679	21
		100.0%	97.0%	3.0%
Q12G. あなたのキリスト教主義学校への関与についてお聞きします。当てはまるものを選んでください。	通ったことがある	108	99	9
		100.0%	91.7%	8.3%
	通ったことはないが、関わったことがある・関りのある人がいる	168	161	7
		100.0%	95.8%	4.2%
	上記のいずれにも該当しない	424	419	5
		100.0%	98.8%	1.2%

有意差はありつつ、選んだ人の実数が少ないということ、また質問文をやや勘違いして答えた者もいる可能性のあることを割り引いて考える必要があるが、クリスマスについても、キリスト教主義学校当事者指数の高い者の方が、信仰に結びつく行動をしているということは確認できる。

以上から、 井上ほか (2018) のいう、トリプルな信仰の現われとしてキリスト教式結婚式の人気とクリスマスの国民行事化それぞれが、キリスト教主義学校の隆盛に相関するという言説が、本稿の調査からもいえることが分かった。

さらに本研究では「Q3. あなたは宗教に由来する年中行事に、何歳頃まで原家族単位で行っていましたか。当てはまるものを 1 つずつ選んでください。ここでは数年に 1 回程度の頻度でも、行っていたと考えてください」という設問を設け、お盆の先祖のお迎え、除夜の鐘付き、初詣について聞いている。

このそれぞれについて Q12G とクロス集計する（ 表3-3-8、 表3-3-9、 表3-3-10）。

**表3-3-8.** Q12G キリスト教主義学校関与度 × Q3①原家族とのお盆の行事.

① お盆にご先祖様や亡くなった家族を家にお迎えする準備作業					
		合計	今でも原家族と行っている(過去は行っていなかったが、今現在は行っている場合も含む)	今は原家族と行っていないが、過去に行っていた	原家族と行った記憶はない
全体		700	291	72	337
		100.0%	41.6%	10.3%	48.1%
Q12G. あなたのキリスト教主義学校への関与についてお聞きします。当てはまるものを選んでください。	通ったことがある	108	52	8	48
		100.0%	48.1%	7.4%	44.4%
	通ったことはないが、関わったことがある・関りのある人がいる	168	78	28	62
		100.0%	46.4%	16.7%	36.9%
	上記のいずれにも該当しない	424	161	36	227
		100.0%	38.0%	8.5%	53.5%

**表3-3-9.** Q12G キリスト教主義学校関与度 × Q3①原家族との除夜の鐘つき.

② 除夜の鐘つき					
		合計	今でも原家族と行っている(過去は行っていなかったが、今現在は行っている場合も含む)	今は原家族と行っていないが、過去に行っていた	原家族と行った記憶はない
全体		700	94	38	568
		100.0	13.4	5.4%	81.1%
Q12G. あなたのキリスト教主義学校への関与についてお聞きします。当てはまるものを選んでください。	通ったことがある	108	23	7	78
		100.0%	21.3%	6.5%	72.2%
	通ったことはないが、関わったことがある・関りのある人がいる	168	32	14	122
		100.0%	19.0%	8.3%	72.6%
	上記のいずれにも該当しない	424	39	17	368
		100.0%	9.2%	4.0%	86.8%

**表3-3-10.** Q12G キリスト教主義学校関与度 × Q3③原家族との初詣.

③ 神社やお寺への初詣(はつもうで)					
		合計	今でも原家族と行っている(過去は行っていなかったが、今現在は行っている場合も含む)	今は原家族と行っていないが、過去に行っていた	原家族と行った記憶はない
全体		700	416	79	205
		100.0%	59.4%	11.3%	29.3%
Q12G. あなたのキリスト教主義学校への関与についてお聞きします。当てはまるものを選んでください。	通ったことがある	108	73	13	22
		100.0%	67.6%	12.0%	20.4%
	通ったことはないが、関わったことがある・関りのある人がいる	168	114	16	38
		100.0%	67.9%	9.5%	22.6%
	上記のいずれにも該当しない	424	229	50	145
		100.0%	54.0%	11.8%	34.2%

これは（ 表3-3-8）カイ二乗<0.01 で有意差がある。キリスト教主義学校に通ったことのある者ほどお盆の迎え火を「今でも原家族と行っている」人が多く、キリスト教主義学校と関わりがない者は「原家族と行った記憶はない」者が多い。これも（ 表3-3-9）カイ二乗<0.01 で有意差がある。実数は多くはないが、キリスト教主義学校の当事者性が高い者ほど、原家族と除夜の鐘の鐘付きに今でも行っている。

この初詣についても（ 表3-3-10）カイ二乗<0.01 で有意差がある。「キリスト教主義学校に通ったことのある」者と「キリスト教主義学校に通ったことはないが、関わったことがある・関りのある人がいる」者の間には差があまりないが、それら何らかの意味でのキリスト教主義学校当事者と、キリスト教主義学校への関わりのない者との間では、差がある。

なお、『 「現代日本における宗教教育の実証的研究」(1998~1999) 報告書』では、「あなたは今年の初詣はどうしましたか」という問いに「家族と行った」「家族とは別に行った」「行った家族もいるが自分は行かなかった」「家族の誰も行かなかった」「その他」の選択肢を用意してある。宗教系大学生のみについてであるが、大学の宗教別のクロス集計をしている（ 井上ほか 2000 p.44）。宗教系大学の合計だと「家族と行った」24.5%、「家族と別に行った」22.4%、「行った家族もいるが自分は行かなかった」17.6%、「家族の誰も行かなかった」23.1% である。カトリック系大学生は「家族と行った」38.3%、「家族とは別に行った」21.7%、「行った家族もいるが自分は行かなかった」13.5%、「家族の誰も行かなかった」21.7% で、宗教系大学の合計の選択肢の 1，2 の合計が 46.9% であるのに対して、カトリック系大学では 60.0% である。プロテスタント系大学ではそれほど宗教系大学の合計と差はなかった。因みに神道系大学では「家族と行った」23.0%、「家族とは別に行った」25.4%、「行った家族もいるが自分は行かなかった」23.3%、「家族の誰も行かなかった」22.3%である。選択肢 1，2 の合計が 48.4% で宗教系大学の合計より少し多いだけで、カトリック系は神道よりも初詣に積極的という結果が出ている。同書の分析では「カトリック系が約 6 割、天理大と仏教系他学科（注記；この調査報告書では仏教系大学について一般学科と仏教学科を分けた集計を行っている）と神道系とプロテスタント系が約 5 割であるのに対して、仏教学科 2 割 5 分、創価大 1 割強とあとの 2 者が比較的割合が少ないことが理解できる」（ 井上ほか 2000 p.44）と記される。当然、これはキリスト教主義学校のみに焦点を当てた研究ではないので、この分析は妥当ではあるが、カトリック系が神道系を超えているというある意味非常に意外な結果については特にこだわっている印象はない。しかし我々の視点からこの 20 年前の研究を見返すと、「トリプルな信仰」に通じる寛容性の涵養をカトリック系の大学はこの時期既に行っていた可能性があるとも評せて、我々の調査結果に通じる結果がすでに出ていたともいえる。

以上の 3 つの我々の調査による表から、キリスト教主義学校当事者の方が（通学経験者と緩やかな当事者との差はケースバイケースではあるが）、幼少期に原家族とお盆や除夜の鐘や初詣など、キリスト教「以外の」宗教行事に積極的に触れてきていることが分かる。つまり、この結果は排他的なキリスト教像とは逆とも評せるし、またキリスト教主義学校への期待も、キリスト教という特定宗教への期待というよりは宗教教育による情操の涵養への期待があることが想定できる。

例えば幼稚園から大学院修士課程まで一貫して同じプロテスタントの学校法人の学校に通っていた大学院生（インタビュー当時）A さんは次のように語っている（調査日 2019 年 5 月 31 日）。

片山:ご家庭でクリスマスには歌を歌うとか、キリスト教徒としてじゃなくて構わないんですけど、なにか行事としてありましたか?

Aさん:クリスマスは大体ケーキを買ってきて、料理も豪華にしたりしていて、やっぱり家族そろってるときはみんなで食べたりしてました。

後藤:家族そろってクリスマスがあったのはいつ頃までですか?低学年のころ?段々ばらばらになってしまうというか、忙しくなってとか、友達と過ごすとかあったと思うんですけど。

Aさん:高校頭くらいまではみんなで祝っていたかな、という感じで。

後藤:それはお兄さんも大学頭くらいまで?

Aさん:そうですね、大学入った頃くらいで。兄もそのときには結構忙しくなってたみたいで、あんまりもう一緒に祝うってことはなかったんですけど、大体クリスマスじゃないときの日曜日に、振替で何かうまいものでも食べようか、みたいな感じで格好つけてクリスマスっぽいことをしたことはありました。

（中略）

Aさん:うちの親父の、○○県の実家がかなり浄土真宗の信仰が強くて、家族全員がお経を唱えられるので、私もそっちの方に行くとみんなと正座しながらお経を唱えて、教本もあって、数珠をつけてお経を唱えるというのが慣例なので。

片山:初詣も?

Aさん:初詣も行きます。

後藤:お経を読めるほどに熱心な信者ということ?

Aさん:そうですね。祖父がそういう浄土真宗のお寺の方で、住職ではないんですけど、一時期見習いみたいなことをやっていたころから、かなり熱心にやっていたみたいで。うちの父親も高校はそういう宗教の学校ということで、仏教というか、そっちの方で学んでたということで。

後藤:仏教?真宗でない仏教系の学校?

Aさん:はい、学校で、高校は宗教を学んでたそうです。

後藤:それは何宗の学校?

Aさん:それが浄土真宗……ちょっとすみません、詳しい学校までは聞いてなかったんですけど、だからお経とかは学校とかで普段やっていて、私たちが讃美歌歌っているのと同じくらいに、父親は経を唱えてたんだなって。

これまでも本稿で折々そのインタビュー記録を参照してきたように、A さんは入信してはいないが非常にキリスト教にシンパシーを抱いている。母親もお兄さんもAさんと同じ学校法人の学校への在籍経験もある。それゆえ、なんとなくというよりは積極的にこの学校法人を選んでいるといえる。そういうキリスト教主義学校のシンパサイザーである A さんの育った家庭は、キリスト教も仏教も神道もいずれにおいても宗教行事には積極的であり、その点で、 井上ほか (2018) のトリプルな信仰を裏づける結果となっている。

また A さんは、このような父親がAさんのキリスト教への親和的な態度を肯定的に見守っていたともいう。

後藤:お父さまは仏教徒としてのキリスト教への抵抗よりは、自分は浄土真宗の信仰心が篤かったから、同じようにキリスト教でも信仰心が篤い方がいいと考えていたんですか?

Aさん:どうなんですかね……完全にキリスト教徒になれとか、そういう感じではもちろんないんですけど。

後藤:信仰心を養うことは悪いことではないと?

Aさん:そうですね、そうとは思ってると思います。なので教会とかも行けるなら行った方がいいんじゃないの、とかも言っていましたし、そういうところでせっかく関わるなら関わりを持ち続けたほうがいいと、そういうのは言ってくれるので、宗教を心の支えにじゃないですけど、自分の生きる道のひとつの支えにしていいんじゃないかということは言われたことがありますね。

後藤:お父さまと、そういう人生とか将来について、お忙しそうだけど話す機会が時々あるんですか?

Aさん:そうですね、普段は忙しいんですけど、休みの日にはそういう話をしました。

つぎに入信とご利益の関係を問うた Q49 と Q50 を見てみる。この二つは別のことを聞いたつもりであったが、調査終了より後の時点で気づいたが、実質ほぼ同じ内容となる。

Q49 は「以下の宗教において、入信していなくても教えを守ることによって、救済やご利益（ごりやく）が得られると思いますか。当てはまるものをそれぞれ選んでください」という設問で神道と世界三大普遍宗教合わせて 4 宗教について同じ尺度で聞いている（ 表3-3-11）。「そう思う」の率が、神道、仏教、キリスト教、イスラム教の順に減ってはいるが、「そう思う」と「ややそう思う」を足しあげた数値でいうと、神道 41.1%、仏教 43.6%、キリスト教 32.6%、イスラム教 19.0%で、イスラム教に較べればキリスト教が目立って、仏教、神道より少ない訳ではない。

**表3-3-11.** Q49 単純集計 (4宗教各々)。入信しないが教えを守ることによる救済の有無の推測.

	合計	そう思う	ややそう思う	あまりそう思わない	そう思わない	よく分からない
1. 神道(神社神道)	700	110	178	115	110	187
	100.0%	15.7%	25.4%	16.4%	15.7%	26.7%
2. 仏教	700	91	214	119	99	177
	100.0%	13.0%	30.6%	17.0%	14.1%	25.3%
3. キリスト教	700	67	161	160	127	185
	100.0%	9.6%	23.0%	22.9%	18.1%	26.4%
4. イスラム教	700	40	93	158	187	222
	100.0%	5.7%	13.3%	22.6%	26.7%	31.7%

このキリスト教について Q12G を独立変数としてクロス集計すると、 表3-3-12 のようになる。キリスト教主義学校への通学経験者は「そう思う」が相対的に多く、緩やかな当事者は「ややそう思う」が多く、非当事者は「よく分からない」が多い。このようにキリスト教主義学校当事者指数が高いほど、キリスト教において、「入信していなくても教えを守ることによって、救済やご利益（ごりやく）が得られると思う」という人が多いということが分かる（カイ二乗<0.01）。またそれと同時にキリスト教主義学校への通学経験者は「よく分からない」が最も少ない。また大きな差ではないが「そう思わない」も最も多い。

**表3-3-12.** Q12G キリスト教主義学校関与度 × Q49 のキリスト教における入信しない者の救済の有無の推測.

			そ う 思 う	や や そ う 思 う	あ ま り そ う 思 わ な い	そ う 思 わ な い	よ く 分 か ら な い
全体		700	67	161	160	127	185
		100.0%	9.6%	23.0%	22.9%	18.1%	26.4%
Q12G.あなたのキリスト教主義学校への関与についてお聞きします。当てはまるものを選んでください。	通ったことがある	108	20	32	23	21	12
		100.0%	18.5%	29.6%	21.3%	19.4%	11.1%
	通ったことはないが、関わったことがある・関りのある人がいる	168	12	55	47	26	28
		100.0%	7.1%	32.7%	28.0%	15.5%	16.7%
	上記のいずれにも該当しない	424	35	74	90	80	145
		100.0%	8.3%	17.5%	21.2%	18.9%	34.2%

Q50 は Q49 と実質近い内容で、「以下の宗教において、入信していなければ教えを守っていたとしても、救済やご利益（ごりやく）を得ることはできないと思いますか。当てはまるものをそれぞれ選んでください」が質問文で、挙げた宗教および選択肢のカテゴリーは Q49 と同一である（ 表3-3-13）。

**表3-3-13.** Q50 単純集計(4宗教各々)。入信しないと教えを守っても救済されないかどうかの推測.

	合計	そう思う	ややそう思う	あまりそう思わない	そう思わない	よく分からない
1. 神道(神社神道)	700	51	71	168	167	243
	100.0%	7.3%	10.1%	24.0%	23.9%	34.7%
2. 仏教	700	49	59	186	174	232
	100.0%	7.0%	8.4%	26.6%	24.9%	33.1%
3. キリスト教	700	60	108	160	137	235
	100.0%	8.6%	15.4%	22.9%	19.6%	33.6%
4. イスラム教	700	101	103	131	114	251
	100.0%	14.4%	14.7%	18.7%	16.3%	35.9%

これも Q12G とキリスト教をクロス集計すると以下（ 表3-3-14）の通りである。これもカイ二乗<0.01 である。

**表3-3-14.** Q12G キリスト教主義学校関与度 × Q50 のキリスト教における入信しない者の救済の有無の推測.

			そ う 思 う	や や そ う 思 う	あ ま り そ う 思 わ な い	そ う 思 わ な い	よ く 分 か ら な い
全体		700	60	108	160	137	235
		100.0%	8.6%	15.4%	22.9%	19.6%	33.6%
Q12G. あなたのキリスト教主義学校への関与についてお聞きします。当てはまるものを選んでください。	通ったことがある	108	14	14	30	27	23
		100.0%	13.0%	13.0%	27.8%	25.0%	21.3%
	通ったことはないが、関わったことがある・関りのある人がいる	168	13	39	45	33	38
		100.0%	7.7%	23.2%	26.8%	19.6%	22.6%
	上記のいずれにも該当しない	424	33	55	85	77	174
		100.0%	7.8%	13.0%	20.0%	18.2%	41.0%

「そう思わない」「あまりそう思わない」はいずれもそれぞれ、当事者（通ったことがある者）、緩やかな当事者、当事者以外の順に多い。ただし「そう思う」も当事者が多く、緩やかな当事者、当事者はほぼ同じであった。「ややそう思う」は緩やかな当事者が多く、当事者、当事者以外は少なかった。他方「よく分からない」は非当事者が多く、当事者、緩やかな当事者は同程度に少ない。

要するに、当事者や緩やかな当事者はこの見解に賛同しない者が非当事者よりも多いが、賛同する者も非当事者より多い。要は判断保留せずに、キリスト教主義学校に関与している者は、賛同しない側に偏っているものの両極に割れている。非当事者はキリスト教が首尾一貫性を重視し、洗礼というプロセスが重要であるという一般的知見、常識すらなく、そのため「よく分からない」が多いと推察される。他方何らかの意味での当事者はそういう一般的知見があるので「よく分からない」が減る分、そういう一般的知見に引きつられた「そう思う」が多いし、その一般的知見をあえて否定した、使命としてのミッションないしは徳育としてのキリスト教的な見解はさらに多いという結果になっていると考えられる。

次に Q50 から Q49 にまた戻るが、キリスト教式結婚式の是非を問うた Q22 と Q49-3 のクロス集計は 表3-3-15のようになり、カイ二乗<0.01 で有意差がある。キリスト教式結婚式を「好ましいと思う」人の方が、入信せずともご利益があるという見解に「そう思う」を選ぶ人が多く、キリスト教式結婚式が「好ましくない」と思う人の方が「そう思わない」を選ぶ人が多い。これは信者でない者がキリスト教式結婚式を行う場合に、事前講義があったり、本稿でも 濱田 (2001) に即して説明してきた先述のようにバチカンの側でさえ日本のカトリックのキリスト教式結婚式に「霊的指導」という位置づけはしていることにも照応しよう。もっともキリスト教式結婚式について「どちらともいえない」という人は「好ましくないと思う人」よりも、Q49-3 で「そう思う」を選ぶ人が少ない （37.8%に対する26.0%）ことは、意外であったし、この理由はよく分からない。一方 Q22 と Q50-3 もクロス集計してみたが、こちらは有意差が出なかった。

**表3-3-15.** Q22 キリスト教式結婚式の是非 × Q49 のキリスト教における入信しない者の救済の有無の推測.

		合計	そう思う・ややそう思う	あまりそう思わない・そう思わない	よくわからない
全体		700	228	287	185
		100.0%	32.6%	41.0%	26.4%
Q22. キリスト教徒ではない日本人のカップルが、教会で結婚式を挙げることについて、あなたはどのように思いますか。	好ましい	252	107	94	51
		100.0%	42.5%	37.3%	20.2%
	どちらともいえない	411	107	184	120
		100.0%	26.0%	44.8%	29.2%
	好ましくない	37	14	9	14
		100.0%	37.8%	24.3%	37.8%

つぎに Q49 をグルーピングしたものと、「あなたは仮に人生をやり直せて、入学に必要な条件（学力、経済力、交通の便等）がそろっていれば、キリスト教主義の中学校あるいは高校に入りたいですか」という設問の Q35 の回答をグルーピングしたものをクロス集計すると 表3-3-16 のようになり、カイ二乗<0.01 であった。

**表3-3-16.** Q49 のキリスト教における入信しない者の救済の有無の推測 × Q35 仮に人生をやり直せるとしてキリスト教主義学校の中高に入りたいか.

			入りたい・どちらかといえば入りたい	どちらかといえば入りたくない・入りたくない	中学校・高校の選択の際に宗教は考慮に入れない	よくわからない・答えたくない
全体		700	74	251	180	195
		100.0%	10.6%	35.9%	25.7%	27.9%
Q49. 以下の宗教において、入信していなくても教えを守ることで救済やご利益が得られると思いますか。当てはまるものをそれぞれ選んでください。(3.キリスト教)	そう思う・ややそう思う	228	52	65	61	50
		100.0%	22.8%	28.5%	26.8%	21.9%
	あまりそう思わない・そう思わない	287	17	132	77	61
		100.0%	5.9%	46.0%	26.8%	21.3%
	よくわからない	185	5	54	42	84
		100.0%	2.7%	29.2%	22.7%	45.4%

キリスト教について「入信していなくても教えを守ることによって、救済やご利益（ごりやく）が得られると思いますか」という問いに「そう思う・ややそう思う」と答えた者の方が、キリスト教主義学校に「入りたい・どちらかといえば入りたい」という者が多いことが分かる。

以上の面から 井上 ほか (2018) らのいう「トリプルな信仰」は、我々の調査結果からも裏付けられるといえる。

## 4. 調査結果のまとめとその限りでの結論

今回は紙幅の関係もあり我々のウェブモニター調査の結果のうちの主にキリスト教主義学校設置目的を推測させた Q34 と、キリスト教主義学校の生徒イメージを聞いた Q29 の各選択肢に焦点を当てて、分析、論述した。分析の軸は主にキリスト教主義学校当事者指数を示す Q12G 及びキリスト教主義学校への女性皇族の通学への是非を聞いた Q11G を用いた。

キリスト教主義学校設置目的の Q34 の分析結果としては、キリスト教主義学校の設置目的として信者の獲得を挙げる者が、キリスト教主義学校に通った者等、キリスト教主義学校への関与度の高い者の方が、有意差はないが、少ない傾向にある（p値=0.13）ことが示唆された。他方、「信者にならなくても、キリスト教の教えを守ることによって、救われる可能性のある人を増やすこと」を選ぶ者が、キリスト教主義学校に通ったことのある者の方に多い（カイ二乗<0.01）ことも分かった。また「信者の子弟の教育要求への対応」、「教会以外の、キリスト教に触れる敷居の低い舞台としての役割を果たすこと」を選ぶ者も、キリスト教主義学校に通った者等、キリスト教主義学校への関与度の高い者の方に多いことが分かった。

このような結果からキリスト教主義学校の教育目的としては布教という狭い意味のミッションよりは、神に与えられた使命としてのミッションを果たすという広い意味で当事者たちから理解されていることが分かった。またこの「信者にならなくても、キリスト教の教えを守ることによって、救われる可能性のある人を増やすこと」を選ぶ者が、キリスト教主義学校に通ったことのある者の方に多いということは、洗礼と非洗礼で一線を画し、首尾一貫性を重んじるという、従来いだかれてきた日本のキリスト教像とは異なった見方を、キリスト教主義学校当事者たちがとっているということにもなる。これは先行研究の 井上ほか (2018) のいう「トリプルな信仰」としてのキリスト教という考え方に照応する結果であるともいえる。もちろん教育基本法の縛りの影響も勘案する必要はあるが。

他方、キリスト教主義学校の生徒イメージを聞いた Q29 の分析結果としては、 井上ほか (2018) のキリスト教主義学校の女子は「きれい、金持ち、キリスト」の 3K であるという点については、金持ちである点については「裕福である」という選択肢を設けたところ、それは、「該当なし /答えたくない」を含めた 19 個の選択肢のうち「該当なし /答えたくない」を除き 4 番目に選ばれ、ある程度妥当した。しかしきれいという点については「容姿端麗である（みめうるわしい） 」という選択肢を用意したが、これは 19 個の選択肢のうち「該当なし /答えたくない」を除き 18 番目という低い順位で、なおかつこれは Q12G のキリスト教主義学校関与度指数とも相関しなかった。要するにこのきれいであるという点はまったく妥当しないことが分かった。このような結果となったのは今回 Q29 で女子に限らず、キリスト教主義学校の生徒・学生全般のイメージを聞いていることもさることながら、先行研究の 佐藤 (2006) や 井上ほか (2018) の言及対象とした時代よりも現在、キリスト教主義学校の女性バイアスが弱まっている可能性も大きいと考えられる。

また 井上ほか (2018) ではキリスト教主義学校では愛と奉仕の精神を教育目標に掲げるところが多いとのことであったので、「他人を思いやる心が豊かである」を Q29 の選択肢に入れた。これは 19 個の選択肢のうち「該当なし /答えたくない」を除き 6 番目であまり多く選ばれているともいえないが、Q12G とクロス集計すると、キリスト教主義学校当事者の方が有意にこれを選ぶ者が多かった。しかし実際に通ったことのある者よりも当事者性の低い、緩やかな当事者の方がこれを選ぶ者が多かった。その点で、これは「他人を思いやる心」を涵養させうるだろうという家族等周囲の期待がその数字に表れていて、実際の通学経験者はそれほどでもないと思っていると考えられる。

加えてこの Q29 で「該当なし/答えたくない」を除き最も選ばれたのは「外国語が得意である」である。女性皇族のキリスト教主義学校関与の是非を問う Q11G とクロス集計すると、キリスト教主義学校への関与を是とする者ほど、「外国語が得意である」を選んでいることが分かった。またこの Q29 でその次に選ばれたのは、「教養が豊かである」で、これも女性皇族のキリスト教主義学校関与の是非を問う Q11G とクロス集計すると女性皇族のキリスト教主義学校への関与を「望ましい」と考える者は、「教養が豊かである」を選ぶ人が「どちらともいえない」と考える人や「望ましくない」を選ぶ人より、有意に多かった（カイ二乗<0.01）。またこれを Q12G とクロス集計すると、キリスト教主義学校にかかわりの高い者ほど、キリスト教主義学校出身者を「教養が豊かである」と自認している（カイ二乗<0.01）ことが分かった。

これらの結果から、女性皇族のキリスト教主義学校関与を是と捉える層からの皇室外交への期待がうかがわれる。また「外国語が得意である」と「教養が豊かである」が、Q29 で「該当なし /答えたくない」を除き一番目、二番目に選ばれ、「他人を思いやる心が豊かである」が六番目であることを考察すると、キリスト教主義学校の意義を、宗教教育をはじめとする情操の涵養のレベルで捉えるよりも、外国語や教養などのより実利レベルで捉える人が多いということになる。もちろん、情操教育も広い意味で、教養に結びつくし、女性においては結婚戦略とあわせ、社会階層・地位上昇の契機になる可能性も、調査票の設計時には我々は考え、その限りでは実利に結びつくが、その前提となる女性バイアスそのものが弱くなっていることは、 佐藤 (2006) に即して本稿でも先述した通りである。

## 5. 考察

### 5.1 クリスマスとキリスト教結婚式とキリスト教主義学校の隆盛の相同性の指摘の是非

1. で 井上ほか (2018) の、日本人のトリプルな信仰志向にキリスト教も組み込まれるという指摘に対して、キリスト教は洗礼というプロセスを要するし、丸山らのいう首尾一貫性も求めるので、ダブルな信仰とトリプルな信仰とには断絶、距離があるという反論もある。いいかえると現実にはトリプルな信仰になり得てはいない可能性を吟味する必要があるのではということを述べた。一方で井上らはそのようなトリプルな信仰の根拠としてクリスマスとキリスト教結婚式とキリスト教主義学校の隆盛を挙げていた。

この三つが挙げられていることについてそのウェブモニター調査上の妥当性は、3.3 で本稿の調査からもいえるということを示したが、ここでは文献的にそれを吟味する。

クリスマスに関しては日本の場合、いっさい宗教色はなく、生活上の習俗と化しているといわれる。また、キリスト教式結婚式にしても先述の 濱田 (2001) でも指摘されているように、一部のキリスト教教会の側としては布教の一環として、人びとがキリスト教に触れる機会を提供しキリスト教のシンパを増やすという目的意識が、多かれ少なかれあるにせよ、利用者の方は習俗としてのクリスマスに毛が生えたようなものというレベルの認識である可能性は否めない。この習俗なのか宗教なのかは、本研究のテーマでもあり 井上ほか (2018) のテーマでもあるが、習俗の延長上にある実利という部分も重要である。宗教教育と実益に直結する英語教育とは緊張を孕んできたし、今ではなおのこと、就職率の良さや進学先の良さ等の進路で選ばれている面が強くなっていることは間違いない。実際英文読解における聖書・キリスト教知識の必要性を主張する 石黒 (2004) も、その主張の根拠は語学スキルと異文化理解・コミュニケーションという実用性を基盤としている。

キリスト教主義学校もクリスマスとキリスト教式結婚式と併記して捉える限り、これらは総じてキリスト教という部分は習俗として及びその延長上にある実利のみのレベルで受け取られ、習俗としてのキリスト教のイメージ的な格好良さや進路といった実利面で選好されていると一応纏められるであろう。

### 5.2 習俗と信仰の連続性の有無

しかしキリスト教と鋭い緊張関係をもつもののキリスト教の源流であるユダヤ教が、習俗、生活習慣上の規律を重んじる宗教でもあったとされる点も考慮に入れてこの問題を再吟味すべきだろう。ユダヤ教では割礼その他独自の生活習慣を大切にし、それにより国土を失った散り散りの民族の統合を図っていた。そのような習俗の遵守を宗教上の最大の課題と考える律法主義者（ファリサイ人）たちに対して内面の信仰の重要性を唱えたのがイエス・キリストであり、イエスのそのような側面を強調したのがパウロで、そこから普遍宗教の側面を前面にだしていったという見方もある（ 波多野 1979） ^21^。その点でキリスト教はユダヤ教の律法主義者的な側面のアンチテーゼとして出てきた部分はあり、それはキリストが磔刑に処せられる理由の一つともされている。しかし、ユダヤ教の聖典旧約聖書もキリスト教の聖典でもあり、習俗のみということは批判されるにせよ、1.2.2 でリスペクタビリティについての 佐藤 (2006) の言及への 井上 ほか (2018) のミス・リーディングに関連づけて先述したように、習俗などの形から入って内面の信仰に至るという筋道は一つの道、信仰の形として想定されてしかるべきということにはなる。パウロ自身が元々ファリサイ派の律法主義者であった。青野太潮『パウロ』で「パウロがユダヤ教の立法遵守に熱心なファリサイ人であったことは間違いない」( 青野 2016 p.7)という。

しかし、イエスを信じるに至った回心後のパウロは違う。「ヘレニスト」といわれる「律法からの自由」を唱えた一派がいて、「パウロは、まさに「ミイラ取りがミイラになる」ようにして、そのヘレニストたちの考え方へと回心した」( 青野 2016 p.15) といっている。また同書 ( 青野 2016 p.70) は「コリント人への第一の手紙」を論じる中で、「パウロの捉え方は、神は律法を遵守するわざや行いがまったくない不信心な者をそのままで義とする(正しいとする)方であり、そのような神を信じ、そのことを明らかにしたイエス・キリストを信じる信仰によってのみ、人は救われるのだという、いわゆる「信仰義認論」に深く通じるものであった」」と指摘する。この「信仰義認論」がこの青野の著書のキーワードの一つで、101 頁で「「信仰義認論」とは、通常、人は律法の行ないによってではなく、イエスをキリストと信じることによってのみ、神の前で義(正しい)とされると捉える理解である」( 青野 2016 p.101) という。その「信仰義認論」と対を成すのが「行為義認論」である。「パウロは、「キリストは律法の終わりとなられた」(ローマ人への手紙 10 章 4 節)との理解を基に、神が義とするのは、律法を正しく守って贖罪の儀式をなす「行為」によってではなく、ただ「信仰」によってなのだと考えている。神学研究の用語でいえば、行為義認論ではなく、信仰義認論を展開しているのである」( 青野 2016 p.133)。

したがって波多野や青野のパウロ解釈に従えば、律法という形よりは信仰という内面の方が重要ということになる。青野も同書の随所で指摘するように、パウロ理解も人によって贖罪論にも重きを置くなど、違いはあるという。しかし贖罪論に偏したパウロ理解は間違いだという。かれの贖罪論への肯定的言及はあくまでも、仮にそうだとしても、という譲歩的表現のレトリックに過ぎない。「第3章でも述べたように、パウロは贖罪論を内容とする極めてユダヤ的な伝承を引用しつつも、そこに信仰義認論に基づく彼自身の修正の言葉を挿入している(ローマ人への手紙 3 章 24‐25 節)。その伝承の部分だけを取り出したて、あたかもパウロが積極的に「イエスの贖罪」について論じているかのように解釈する人も多いが、そのような誤解をしてはならない」( 青野 2016 p.192)。

本稿の共著者たちは宗教学や神学の専門家ではないし、共著者一人一人の信仰は確認していないが、筆頭著者はクリスチャンではない。よって、青野のパウロ解釈の是非については不詳である。ただここの部分については青野と波多野はほぼ同一で、かおかつ筆頭著者のもともとの研究対象は中井正一で、中井は三木清のパスカル解釈に大きな影響を受け、なおかつ三木は波多野から強く影響されている。よって、ここは青野の理解を是としたい。

そうであるとすると、行為よりも信仰のほうが大事だということになり、本節 5.2 の冒頭とは、一応、逆方向の結論になる。ただ、数パラグラフ前の引用を繰り返すが、「パウロの捉え方は、神は律法を遵守するわざや行いがまったくない不信心な者をそのままで義とする（正しいとする）方であ」( 青野 2016 p.70) るとすると、洗礼を受ける儀式や学習も「行為」の最たるものの一つではないだろうかとも思う。よって本節 5.2 の冒頭とは、一応、逆方向の結論になるが、それでも場合によってトリプルな信仰を補強する材料にもなると思われる。

### 5.3 首尾一貫性と洗礼と「トリプルな信仰」の吟味

キリスト教側からすると、首尾一貫性のあるキリスト教を理解し、一定の講義を受講し洗礼というプロセスを経ないとキリスト教徒になったとはいえず、そのような正真正銘のキリスト教徒は日本の総人口の0.8%しかいない。そのことからキリスト教は日本において根づいていないと結論づけられることになる。しかしキリスト教にふれる、クリスチャン以外の一般国民の側からすると、洗礼のようなイニシエーションの儀式がなくても仏教や神道ならば信徒であると名乗れて、キリスト教には洗礼というプロセスがあるので正規のキリスト教徒でないにせよ、 井上ほか (2018) が示唆するように、神道の信徒や仏教徒と近い水準でなら自分はキリスト教徒であるという意識をもつという可能性があるようにも思われる。それが先にみた、Q34 で「信者にならなくても、キリスト教の教えを守ることによって、救われる可能性のある人を増やすこと」を選ぶ者が、キリスト教主義学校に通ったことのある者の方に多いことに表われている。

また Q29 のキリスト教主義学校の生徒・学生のイメージ、及び Q26 のキリスト教徒、Q27 のキリスト教の聖職者、いずれのイメージにおいても、「論理の整合性を尊ぶ」を選ぶ者が極端に少なかったことも、このことを補強する材料といえるであろう。というのも、論理の整合性を尊ぶことは首尾一貫性を求めることであり、「はじめにロゴスがあった」というヨハネ福音書冒頭のように唯一の聖典という基準があるので、洗礼後と洗礼前、自分たちと他者たちをその基準への意識によって明確に峻別することにもなる。そうすると、仮にその選択肢が多く選ばれていた場合、洗礼の有無という重要な基準をスルーする、トリプルな信仰という発想は、ロジカルにも否定されることになるであろう。一方、今回の調査結果はその逆になったため、そのことがトリプルな信仰という 井上ほか (2018) の着想への補強材料になる。

なお洗礼が、トリプルな信仰の考え方を妨げる障壁のように考えられる一方で、 佐藤 (2006) は 1926 年~1930 年のキリスト教主義学校卒業生の洗礼率が平均 50% 前後で推移している（ 佐藤 2006 p.17）ことについて、学生時代に受洗したことがあるというもののいまでは仏教の法事を普通に執り行う佐藤自身の親類の老婦人の例を出しながら（ 佐藤 2006 p.15）、とりあえずは受洗してみたけれど、という人も少なからずそのなかに混ざっていたのではないのかと推測する ^22^。したがってその意味で、このような人びとは、そもそも昔であっても、洗礼をしつつトリプルな信仰に近いスタンスをもっていた可能性もあるといえよう。そうはいっても日本において洗礼というプロセスの敷居は高い。家族と違う宗教を場合によって選び取る、また洗礼前に講義を受ける等々、諸々の手続きも要する。先祖の墓の維持・管理という問題まで生じうる。他方、欧米であればキリスト教徒になったからといって家族と違う宗教というケースは日本よりは少ないし ^23^、それ以上に幼児洗礼のある宗派の場合、キリスト教徒であることは自明でそのことをさほど自覚せずに済む。その点でトリプルな信仰のひとつとしてのキリスト教というものは、洗礼という重大プロセスを無視しているとはいえるが、欧米のクリスチャンの場合、そもそも洗礼というプロセスを当人は意識せずに済むケースも少なくないという点にも留意が必要であろう。

先に 2.1 でみたように、キリスト教主義学校の設置目的の推測の設問である Q34. において選択肢 8 の「キリスト教の信者を増やすこと」を選んだ者は全体で 13.0% であったのに対して、選択肢 9「信者にならなくても、キリスト教の教えを守ることによって、救われる可能性のある人を増やすこと」は全体で13.3% となっており、ごく僅かな差で有意な差とはいえないが、「キリスト教の信者を増やすこと」より多かった。しかも Q12G とそれぞれの選択肢を選んだ人、選ばなかった人とでクロス集計すると、これも先にみたように、選択肢 8 の「キリスト教の信者を増やすこと」を選ぶ人はキリスト教主義学校の通学者・通学経験者の方に有意差はない（なお 2.1 で先述のように検出力不足故、有意差が出なかったと我々は考察した）が少なく、逆に、選択肢 9 の「信者にならなくても、キリスト教の教えを守ることによって、救われる可能性のある人を増やすこと」はキリスト教主義学校の通学者・通学経験の方が有意に多かった（カイ二乗<0.01）。キリスト教主義学校教師であるかどうかも本来聞くべきであるが、残念ながら今回それらに聞いた結果ではないものの、少なくとも通学経験者の回答の比率から推すと、キリスト教主義学校の教育において洗礼を受ける人という意味での狭義の信者を増やす意図よりは、キリスト教の精神を理解し、キリスト教的な徳目を守る人を増やすという意図が、強くなっていると考えられる。同志社は 「徳育としてのキリスト教」を標榜しているが、他学も含めてそれに近い傾向があるといえよう。

なお津城寛文はユニテリアンその他の「自由キリスト教」が 1880 年代後半に日本に入ってきて、それと東大宗教学講座の創設者姉崎正治の比較宗教学や万国宗教大会 (1893) の影響もあって、一部のキリスト教において、キリスト教の相対化、キリスト教の日本化の動きが生じたという（ 井上ほか 1997 p.124）。その流れの中に玉川学園の創始者小原国芳、桜美林の創始者清水安三も位置づけられるという。「小原国芳や清水安三らも、神道・仏教・儒教および習俗・習慣と断絶しない、日本化したキリスト教をとなえ、正統的なキリスト教教義にもとづく宗教教育ではない、超宗派的な、あるいは宗教色のうすれた人間 教育（小原のいう「全人教育」など）をこころみた。ここには「一部のキリスト教」の自由キリスト教的な、あるいは日本化したキリスト教という姿勢が共有されているようにおもわれる」（ 井上ほか 1997 p.124~125）。ここで「一部のキリスト教」と記されているが、次に引く文でも津城は「一部の」という言葉を二度使う。「右にみたように、キリスト教は明治初年の直輸入なものから、明治なかば以降、その一部が「自由キリスト教」化あるいは日本化したが、それに応じて、キリスト教系の宗教教育の一部も、比較宗教的な教育へ変化している」（ 井上ほか 1997 p.125）。

戦後間もなくの頃、カトリックの立場からは遠藤周作などが、またプロテスタントの立場からは ICU 教授の武田清子などが日本におけるキリスト教の受容と土着化の問題を考え抜いた。土着化、日本への定着の問題と日本のキリスト教主義学校の隆盛とを関連づけた研究は 佐藤 (2006)、 井上 ほか (2018)、 クリーグ(2017) 以外管見の限りないが、このような日本化したキリスト教という問題を、キリスト教主義学校はみずからの課題として考え、キリスト教主義学校の「ミッション」を洗礼する人を増やすという狭い意味での布教よりは、キリスト教的な徳目を守る人を増やすという意味、あるいは自らに与えられた「使命」を自覚する人を増やすという意味に変えていることが想定される。津城は慎重に前パラグラフでの引用で「一部のキリスト教」「キリスト教系の宗教教育の一部」と、控えめに主張するが、一部というよりは全般的な傾向として超宗派的な宗教教育がなされている可能性が窺われる ^24^。

そのことは今回のウェブモニター調査においても、Q34 において直接布教を目的とするという答えよりも、入信せずともキリスト教の教えを守ることで救われる人を増やすことという回答が多かったし、後者はキリスト教主義学校当事者ほどその傾向が強かったことからも、一応裏づけられるといえる。

### 5.4 布教・ミッションの目的と、顕在機能、潜在機能

しかし布教・ミッションの目的は一つではなく複合している。したがってこういう複合している目的の問題を扱う際は、ロバート・キング・マートンの、順機能・逆機能、顕在機能・潜在機能の議論が有用である（ マートン 1949=1961 pp.45-77）と思われる。

ミッションの初発の動機あるいは顕在機能としては救われる人を増やすという愛他精神が強くあると考えられる。

他方、ミッションの副産物は当然あり得て、それらは初発の段階では潜在的機能といえる。およそほとんどの宗教において善行をすることは、みずからの救いへの一里程である。キリスト教に即していうと、仮に入信せずんば救済されずとみなされるとすれば、他者を入信させることはキリスト教の立場からすると他者の救済に貢献する善行の最たるものであろう。よってみずからの来世での救済をより確かなものにする可能性があると考えられる。

さらにミッションの機能としては教団の経営基盤の確保の面もあり、これも当初は潜在機能であったと考えられる。というのも基本的にキリスト教も仏教もその初期において指導者層は托鉢のような形で布教しており、施しによって生活の糧を得ていたと考えられる。しかし聖職者が贅沢をしようが質素に生きようが、祈りと布教と学問と写本・写経のみに勤しんで、最低限の農作業はするもののほとんど生産活動をしない聖職者層が拡大すればするほど、またたとえ中世であっても完全な自給自足経済ではなく貨幣での取引が少しずつ浸透したであろうから、そうなるにつれ、聖職者層を養う財政基盤の確保は必須となる。

また日本でもヨーロッパでも教会や寺院が学問の中心であった歴史があり、庶民の子どもが知識を身につける数少ない術の一つが、宗教施設に入門することであった。また日本でもヨーロッパでも支配階級の嫡子以外の男子の子どもはお家騒動をさけるため修道院や寺院に入れさせられるケースも少なくなかった。したがって被支配階級の子弟の階層上昇と支配階級の嫡子以外の男子の階層再生産の舞台として宗教施設が機能した可能性はある。その場合のミッションを考えると潜在機能か顕在機能かは措いて、本音の部分では熱心な信者の数を増やし、教会や寺院の経営基盤を確かなものにするということが充分に想定されうる。なぜならそれに貢献した者は教団内での地位が向上する可能性が増えるからである。

しかし親が階層再生産のために入信させようとしても当人は信仰心から親の望まない、托鉢修道会への入会を求める等、聖職者になる当人の初発の意識としては純粋に神のことを思う意思の貫徹が大きなミッション（使命）であったケースもあると考えられる。

### 5.5 以上を踏まえた、キリスト教主義学校関係者のミッション、他者救済の形の推測

キリスト教主義学校設立当初の目的は、その意味で、救われる人を増やすというということが顕在的機能として高かったと考えられ、その点で、狭義の布教とミッションとが結びついていたと考えられる。しかし無限抱擁的な雑食文化の日本において、教義として首尾一貫性のある、あるいは首尾一貫した節操を信徒に要求するキリスト教は、根付きにくいと実感させられる体験を重ねるにつれ、あるいは丸山真男その他のそういった論述を耳にするにつれ、意識的にか無意識的にかは措いて、キリスト教主義学校関係者の側も自己利益ではなく他者の救済を考える場合には、入信までは求めず、キリスト教的徳目を守らせることで他者の救済を考えるようになったと可能性も予想される。また前節で見た津城のいうような「自由キリスト教」や「比較宗教学」の影響も、そのように変わる要因として大きかった可能性もある。日本以外のケースとしても、清王朝時代の中国で起きた典礼問題は土着化(祖先や孔子ら中国史上の偉人への崇拝も許容する)による布教を戦術的に行ったマテオ・リッチらイエズス会と、キリスト教の原理的な布教方法にこだわった他会派との対立が背景にあったといわれる。当時は「自由キリスト教」「比較宗教学」などのリベラルな思想が乏しかったこともあり、当時のローマ教皇クレメンス11世はイエズス会の方式を批判し、土着化を許さないという方針で統一させた結果、中国でのカトリックの定着は妨げられることとなった。当時のローマ教皇の決定が覆るのは20世紀になってからであるが、その意味では、19世紀後半以後の日本でのキリスト教学校の隆盛とそこでのミッション（布教・使命）は中国での失敗を繰り返さなかったからという推測は、端から見る限り成り立ちうる。「学校経営として成功するためのキリスト教教育、宗教教育がいかなるものであるかは、あきらかではないにせよ、少なくとも小原国芳、清水安三と反対のことをそろえれば、経営として成功しないおそれがつよい、とはいえるかもしれない」（ 井上ほか 1997 p.140）。さらに、そのことと並行して、信者以外の人びとから認識される救いの形も、日本的なものになった（土着化した）の推測として「キリスト教の信者を増やすこと」よりも、若干ではあるが、「信者にならなくても、キリスト教の教えを守ることによって、救われる可能性のある人を増やすこと」が多いことにも表れていよう。しかも再三先述したように前者の回答はキリスト教主義学校の通学者・通学経験者に有意差はないが相対的に少なく（これについては 2.1 で先述のように、我々は有意差はないがその理由は検出力不足によるものと判断した）、他方、後者の回答はキリスト教主義学校の通学者・通学経験の方が有意差のある形で多い（カイ二乗<0.01）という結果にもなっている。　　

このことはキリスト教主義学校の存在が、正規の洗礼に比して緩い、事実上の入信に近いものを設け、ミッションのハードルを下げている可能性を示す。あるいはミッションを布教ではなく、生徒自身の人生における使命の自覚と捉えさせている可能性を示す。とはいえここで「緩い、事実上の入信に近いもの」というのが、キリスト教主義学校への在籍・卒業とか、聖書学とかキリスト教概論等の単位取得とか量に明確に指標化できる基準を想定してのものというわけではない。量的な基準によるものというよりは内面の感化、自覚といった質的な変化であろう。もちろん今回の調査で分かるのは教会、教団や学校自身の意図ではなく、あくまでも一般の人びとからみる教団・学校の意図の推測に過ぎないが、意図はともあれ実際の行動において、仮に人びとによるこの推測と逆のことを実際にキリスト教主義学校がこれまでしてきたとすれば、キリスト教主義学校通学者・通学経験者とそれ以外との回答の分布は、今回の調査結果と逆になっているはずである。少なくともミッションという意味では直接の布教よりは、布教の地均しレベルでよしと考えていると推察され、先に 佐藤 (2006) に依拠して申し上げた、ミッションを「使命」の意味に解するものもそこに含まれるといえるであろう。しかも佐藤は生徒自身が神から彼らに課せられた「使命」を自覚するという意味で「ミッション」に言及し、しかもそういう場合もあるというレベルでの言及であったが、キリスト教主義学校の側からすれば、そのような生徒自身の「使命」を自覚させることこそが学校の「使命」、あるいは教員のミッションと考えている可能性もあるといえるであろう。

実際、 佐藤 (2006 p.12) では、長野清泉女学院のウェブサイトでは「神様は、私たち一人ひとりに、何らかの使命をお与えになっています。その使命に気づき、その使命をはたすためによりよく生きる人を育てる学校が“ミッション・スクール”なのです」と書かれていると記される。なお、現在この学校のウェブを確認したが、すでにこの発言はないし、「使命」の言葉も見当たらない。一方、 同校ウェブの「よくある質問」コーナーで「Q 宗教教育はどの程度行うのですか?」という質問に対して、「A 週 1 時間「宗教」という授業があり、キリスト教精神に基づいた“生き方”を学ぶことを主眼としています。決して信仰を強要するのではなく、思いやりの心を大切にする校内のあたたかな雰囲気は、宗教教育によって育まれてきます」と答えていて（最終閲覧日 2022 年 10 月 9 日）、情操教育の側面を強調している。

他方、ミッション・スクールの「ミッション」とは結びつけた説明はしないが、「使命」については日本における代表的なカトリック女子中学校・高等学校のウェブにおいて強調されている。例えば白百合学園中学校・高等学校では「宗教教育」というタブの 「宗教の授業」という項目において「中学校では、学園の歴史や聖書を学びながら、神に愛されている自分に気づき、思いやりの心、感謝と奉仕の心などキリストの教えに基づく価値観を身につけます。高校では、聖書を深く味わい、社会における自分の役割や使命について考えます」（最終閲覧日 2022 年 10 月 9 日）と記され、高校の課程で「使命」を学ぶとされる。あるいは学校法人雙葉学園のウェブでは 「教育理念」のページで、「すべての人を愛されたイエス・キリストのように、自分を含めた一人ひとりを大切にし、その人にしか果たせない使命のあることに気づき、その人ならではの人生を歩めるようにする」（最終閲覧日 2022 年 10 月 9 日）と書かれ、やはりここでも「使命」に言及される。

このような「使命」としての「ミッション」を自覚した人は「信者にならなくても、キリスト教の教えを守ることによって、救われる可能性のある人」という Q29 の選択肢9にも通じうる。

なお、プロテスタントの女子中高ではミッションに繋がる「使命」への言及は見た限りではない。例えばフェリス女学院中学校・高等学校では 「キリスト教活動」という項目があり、「奉仕活動」や「宗教講演会」「パイプオルガンの練習」等、具体的な活動が示されているが、「使命」の文言はない（最終閲覧日 2022 年 10 月 9 日）。立教女学院中学校・高等学校でも 「立教女学院のキリスト教教育」の項目では「礼拝」「ボランティア活動」「土曜集会プログラム」「キャンプ集会」などここの具体的な活動の小項目に分かれるが、「使命」への言及はない（最終閲覧日 2022 年 10 月 9 日）。ただ 「よくある質問」の項目で「今までキリスト教と無縁だったのですが大丈夫ですか?」という問いに対して、「ご心配ありません。もともと私たちの学校はキリスト教とは無縁だった人のために始められました。学校生活の中で、少しずつキリスト教のことが身につくようになります」（最終閲覧日 2022 年 10 月 9 日）と記され、基本的に「布教」の地ならしに近いニュアンスの回答となっている。また先ほどの「立教女学院のキリスト教教育」の項目の「礼拝」において、「日曜礼拝のご案内」という小項目もあり、「聖マーガレット礼拝堂では、在校生・卒業生・学校関係者の方を対象に、月に一回、日曜礼拝を開催しています」と記されている。したがってこの学院では「使命」という意味でのミッションの側面は少ないが、「布教」の意味でのミッションの準備的な行動はなされているともいえる。

なお中高ではなく系列の小学校の方であるが六十年史編集委員会編『六〇年の歩み』(1997、立教女学院小学校）によると、1947 年「日曜日には小学校の礼拝堂で矢崎（哲学・神学を大学で専攻した教諭）による子供のための礼拝が捧げられた。自由参加であったが、学齢に達しない在校生の妹や近隣の児童も参加して、毎主日百人近い出席があり、多い日には二百人を超えた」（ 六十年史編集委員会編 1997 p.184〔最初の( )は後藤ほか本稿執筆者による補記〕）とある。さらに 1949 年には任意参加ではなくなった。「一九四九（昭二四）年四月から学院は主日礼拝に出席することをキリスト教教育の基本方針として位置づけ、日曜日を自分の所属教会に出席するものの他は全員が学校での礼拝に出席する開放日とし・・・この制度は一九八二（昭和五七）年三月まで続いた」（ 六十年史編集委員会編 1997 pp.184-185）。

なお同書では 1989 年の『立教女学院報』41 号を転載している。そこでチャプレンの杉山が「ご承知のように一九八二年度より小中高各校における、それまでの登校日としての日曜礼拝が廃止され、児童・生徒・勤務員はそれぞれの地域の教会の日曜礼拝に参加するようにすすめられ、日曜日は学校の休日となりました」（ 六十年史編集委員会編 1997 p.498）と語る。そのうえで廃止により参加者が減ったことを次のように表する。「現在、日曜日がキリスト教にとって生命線といえるほど大切な日であることを認識している生徒、また御父母がどれだけいるでしょうか。いや教職員の中にもどれだけいるでしょうか。私たちの学校がキリスト教の学校であるということ、 **ミッション（使命） **をもった学校であるということを考えたとき、私たちはなんとしても日曜日には教会生活を送るよう啓発していく責任があるように思うのです」 （ 六十年史編集委員会編 1997 pp.499-500 〔太字は後藤ほか本稿執筆者〕）。このように『六〇年の歩み』 （1997 年、立教女学院小学校）では使命としてのミッションという言葉が転載再録の形ではあるが、記されている。

以上を踏まえると、 井上ほか (2018) の「トリプルな信仰」という見方も、日本式キリスト教というキリスト教の土着化の過程で、全否定はできないと考えられる。先述の話の繰り返しになるが、 濱田 (2001) のキリスト教式結婚式の研究によると、日本における非クリスチャンを対象にした教会外でのキリスト教式結婚式の本格化はプロテスタントの一部の牧師たちによって始められたものもあるという。キリスト教を具体的に知ってもらう機会を得ることで、キリスト教入信への地均しをしようとする意図のあるものであるという。意図としてはこれはあくまでも地均しではあるが、これも広い意味でのミッション、伝道であり、日本における土着化の一つとしても考えられる。

ここまで「トリプルな信仰」という見方にあらわれる、キリスト教の土着化の一つとして、「使命」としてのミッションの話に言及した。つぎに無教会主義が「トリプルな信仰」のいう見方を支える、日本人全般のキリスト教へのイメージ醸成に役立った点を論じたい。

「信者にならなくても、キリスト教の教えを守ることによって、救われる可能性のある人を増やすこと」という着想は、日本で内村鑑三の無教会主義がよく知られていて、内村が 1951 年に日本郵便の切手になるほどに著名であることとも関連している可能性がある。武田清子著『未来をきり拓く大学―国際基督教大学五十年の理念と軌跡―』ではクリスチャン条項をすべての教員に適用する ICU において、ICU 教会に無教会派の教員を加える際に問題になったことがある点が、記されていた。「洗礼を受けず、信仰告白もせず、無教会に「会員」として入会していない無教会信者と称する人たちを「キリスト教」と認めるということは、「キリスト者」の定義をあいまいにするのではないかという意見が教授会で述べられたことがあった」（ 武田 2000 p.111）。もっともこれはメンバーシップをアフィリエートといいかえることにより解決が図られたと記されている。実際「無教会を標榜する内村は、キリスト者になるためには「水のバプテスマ」すなわち洗礼を受ける必要はないと考えた」（ 若松 2018 p.180）。そうであれば信者であるなしは当人の自覚にかかわるようになり、公的に「信者になる」というプロセスは不要となる。武田は別の著書 『背教者の系譜』で「内村鑑三を創始者とする無教会主義は、・・・日本型セクト運動ともいえると思うのであるが、日本のキリスト教会において長く異端視されてきた」（ 武田 1973 p.112）という。そのうえで 「しかし、無教会グループは、今日、日本のキリスト教界の「正統」と一応考えられるキリスト教諸教派、諸グループの中に位置を占めつつある」とも述べる。また内村は再臨運動をする中で、キリスト再臨の日には福音を信じない者にも「贖いの恩寵が光のごとく、万人にあまねくそそがれる」（ 若松 2018 p.180）と考えていた。あるいは堀孝彦は次のように内村の再臨運動を記す。「再臨を、ただ「教会問題」とみなしキリスト信者にのみかかわるものと見るのは大きな誤りだと言って、それが「宇宙問題、世界問題」であるから、万人に訴えて注意を喚起すべきだとしている。その根底には・・・万人救済論がある」（ 堀ほか 1996 pp.140-141 〔・・・は中略〕）。そのような内村の考え方がキリスト教の一般的姿として、日本のクリスチャンの内外から受け入れられ、それにあわせて 武田 (1973) のいうように、プロテスタントの聖職者・研究者界隈でも当初は異端でありつつ次第に受け入れられてきたとするならば、「信者にならなくても、キリスト教の教えを守ることによって、救われる可能性のある人を増やすこと」という回答は選ばれやすいといえる。

また東西宗教交流学会長の田中裕は西田幾多郎のクザーヌスへの着目に関連させて次のように語る。「カトリック教会の司祭（枢機卿）でもあったクザーヌスは、キリスト教以外の異教を排除せず、様々に異なる礼拝のなかに一なる宗教があるという立場を取った。・・・西田もまた、キリスト教を日本の国体に反するものとして排除しようとした幕末から明治にかけての多くの宗教者とは違って、キリスト教を異教として排除することをしなかった。福音書やパウロ書簡の宗教的経験の深さを洞察し、自らの禅の立場において、それを摂取し発展させた。西田とキリスト教を結ぶ媒介となったものは、ドグマに固執する神学ではなく、ドグマを越えるキリスト教の霊性的伝統であった」（ 田中 2020 pp.277-278）。実際、西田は東大で、同志社中退でジョン・ホプキンズ大学にて博士号をとりキリスト教に造詣の深い、倫理の教授元良勇次郎からの影響でウィリアム・ジェームスに強い関心を抱いた（ 苧阪 1998 p.349）という説もある ^25^。ジェームズは『プラグマティズム』を発表する 1907 年より前の、1902 年の著書である『宗教的経験の諸相』で既に霊的な体験の有用性を語っていた。ジェームズは『宗教的経験の諸相』で語ったものを方法論的に体系化し、『プラグマティズム』へと洗練させた。ジェームズのプラグマティズムを突き詰めれば、入信などの儀礼的側面は純粋に形式的なものでしかなくなり、ドグマ自体も有用性というテストに合格する限りにおいてしか認められないと解釈できる可能性を開く。なお森本あんりは『異端の時代』においてジェームズに言及する( 森本2018 p.192~197)。「（前略）・・・ジェイムズの言う「宗教」とは、あくまでも原初に感じられた個人の直接的な情熱のことであって、その後に形成される集団や制度のことではない。組織化された宗教は、「仏教徒であれ、キリスト教徒であれ、マホメット教徒であれ、それぞれの国の因習的儀式」に従って模倣された二番煎じの「退屈な習慣」にすぎないのである」( 森本 2018 p.193)。このような霊的な体験を純粋経験とするジェームズの考えを仏教の「無」と同一のものとした西田は『善の研究』で、主客未分の体験をより自分らしく生きて世界を良くする創造的思考の基盤として解釈した。

このように「ドグマに固執する神学ではなく、ドグマを越えるキリスト教の霊性的伝統」を、日本を代表する哲学者、西田がみいだしていたという点も、日本を代表するキリスト教徒の内村の無教会派と同様、多くの人びとに「入信」という壁を経ない、緩やかに「教えを守ることによって、救われる可能性のある人を増やす」という意識をもたせる背景として、想定できるといえるであろう。

また本研究では基本的に『日本の思想』での丸山真男の論旨に依拠しつつ、首尾一貫性を重んじるキリスト教という丸山の考え方とトリプルな信仰との矛盾の可能性の有無を述べてきたが、首尾一貫性を重んじるキリスト教という考え方は「執拗低音」を唱える後年の丸山の趣旨とは多少のずれも生じ得る。『日本文化のかくれた形』では神道等の、日本の古層を中心の不在とは表現しない。消去法によって外来文化の浸透部分を消していくと古層が現れるという。「そうすると、何もなくなるかというとなくならない。サムシングが残るのです。そのサムシングというものがつまり、原型―その断片をあらわしております。原型はそれ自身としては決して教義になりません。教義として体系化しようとすると外来世界観の助けをかりねばならない」（ 加藤ほか 2004 pp.142-143）。このように古層、原型があるから表層は変わりやすいが根本は何も変わらないのが日本文化の特質であると述べ、その特質はキリスト教の受容にも見られるとする。「絶えず新しいメッセージを求めるということと、新しい刺激を求めながら、あるいはそれ故にか根本的に驚くほど変わらないということ・・・この両面がやはり思想史的な問題としても重要なものになってくるのではないか。たとえばこのことをキリシタンの渡来とその「絶滅」の運命について早くからジ ョージ・サンソンという有名なイギリスの日本史家が指摘しています。「いかなる国民も新しい教えをこれほど喜んで受容する用意のある国民はなかった」。新しい教えというのは一五世紀に来たキリシタンのことです。「けれども他方、これほど伝統を頑強に固持した国民はなかった」 」（ 加藤ほか 2004 pp.121-122)。

このサムソンの発言から丸山は中国韓国のキリスト教と比較して、日本のキリスト教が絶滅するときは綺麗に消えてしまう点を指摘する。「驚くほど変わらないということ」及び「これほど伝統を頑強に固持した」という点からすると、キリスト教も日本思想の「古層」と結合する可能性もあったということを、丸山自身が示唆しているともいえ、そう考えると田中が描く上述の西田のキリスト教観、「ドグマに固執する神学ではなく、ドグマを越えるキリスト教の霊性的伝統」のようなもの、あるいは日本で土着化したキリスト教というものへの接点も丸山は想定していたとも考えられなくもない。キリスト教=首尾一貫した座標軸を提示する宗教、という丸山の『日本の思想』等での整理も、あくまで日本の固有信仰との対比の上でのヨーロッパのキリスト教への理念型的な整理であったとすれば、丸山のキリスト教理解の矛盾も解消することは可能である。丸山はウェーバーの理念型を方法論として採用することを主張していたことも、この解釈の整合性を裏付け得る。

実際この講演記録の著書の「あとがきにかえて」のつもりで当初著したものの丸山の薦めで岩波現代文庫版で一つの章にしたという IV 章において、ICU でのこの連続講演会の企画者で、また丸山同様、思想の科学研究会の7名のオリジナル・メンバーの一人として丸山との交流歴の非常に長い武田清子は、「外来宗教（思想）である“キリスト教の土着化”などということは、ありえないことだという断定にぶつかる時、私は戸惑いを覚える。日本人の内発的思考様式、価値意識には、超越的なもの、普遍的なるものを志向する要素が希薄だということは首肯できることであろう。しかし、それが、全く内在していないと果して断定できるだろうか?」（ 加藤ほか 2004 pp.121-122）とも述べている。

武田の同僚でもあった森本あんりは “Two Types of Religion: In Search of a Better Foundation for Interreligious Dialogue in the Asian Context,” ( International Christian University, Humanities: Christianity and Culture 33 (2002), pp.19-26) において、次のように、2つのタイプの宗教を区別する（以下2段落、同論文による）。「あなたが誰であるか」という宗教と、「あなたが何を信じているか」という宗教である。前者は、あなたが誰であるか、誰の家に生まれたか、どこに住んでいるかに基づいている。生まれながらにしてその宗教に属している。したがって、入信や改宗のプロセスは存在しない。この「あなたは誰であるか」という宗教は他の宗教に対して非常に寛容である。彼らの宗教性は、信条ではなく、適切な儀式を行うことに主眼を置いている。したがって、神道の信者でありながら、同時にキリスト教徒でもイスラム教徒でも何でもなれる、と彼らは考えている。このタイプの宗教は誰でも入会したいと思えば、その人は名誉ある客人として扱われるが、その宗教的地位は生涯「アウトカースト」のままである。

それに対して森本のいうには「あなたが何を信じているか」というタイプの宗教は、一見不寛容であるが、その信仰を持つことに同意する限り、すべての人に開かれていて、包括的である。望むなら、出生に関係なく正会員になることが可能である。キリストには、もはやユダヤ人もギリシャ人も、奴隷も自由人のも、男も女もない。寛容、開放性、包括性というのは、このように厄介な言葉で、コインの裏表であるという。

以上二段落、森本の “Two Types of Religion” から本稿に関わる要点を抜粋したが、これは「トリプルな信仰」に対して、「生涯「アウトカースト」」という条件を付ける限り肯定をすることになるし、「出生に関係なく正会員になることが可能」という条件を適用すれば、逆に明確に否定しているといえよう。そのような寛容の二重性から来る矛盾ゆえ、5段落前に我々が示唆・指摘した、丸山の『日本の思想』と『日本文化のかくれた形』での、微妙なズレも生じるのかもしれない。

例えば森本の『アジア神学講義』(森本 2002 p.211) では、「二重信仰」の問題が取り上げられる。「従来のキリスト教的な理解では、信仰は唯一的であって、一つの宗教をもつということは他の一切を棄てるということである」。しかし「近年第二版が発行された『世界キリスト教年鑑』には、「キリストを信ずるが洗礼を受けていない信徒」ないし「キリストを信ずる非キリスト教徒」というカテゴリーが設けられている」。この年鑑の編者によると「「ヒンドゥー教、イスラム教、仏教などの信徒が、自分の第一の信仰としてイエス・キリストを信じていながら、キリスト教会には属さずに自分の文化的な伝統に留まることを選び、その宗教の内部でキリストを証しつづける」場合である」という。

さらに森本はアジアで深刻なのは、「「宗教」概念の根本的な理解の差に由来する二重性である」として、次のように述べる。「多くのアジア人にとり、宗教は意識的な決断の事柄である以前に、儀礼や習慣などの生活様式に密着した事柄である。これらの「道」を「宗教」 として捉えようとする前提そのものが、実はかなり一面的なものである公算が大きい。つまり、アジア的な「二重信仰」の場合には、キリスト教と他宗教が同一人格において 「並在」するといっても、それはキリスト教的な宗教理解から見た場合であって、他「宗教」の側からすれば、リンゴとミカンのようなカテゴリー内部の衝突ではなく、リンゴとハチミツのように別種の組み合わせとして矛盾なく受け止められていることがある。そもそも人がある宗教を「選ぶ」という理解は、 複数の宗派や教派が手近に混在し、あたかも市場で買い物をするようにその中から自分に適したものを選び取るという市場経済型自由教会型の社会構造を前提している。アジアの大多数の人々は、ある「宗教」の中へと生まれ落ちるのであって、選択や決断を基盤としたこのような「宗教」観を共有してはいない」と述べる。要は、宗教 A と宗教 B は論理学的に排他的であるという従来の発想は、アジアでは必ずしも自明ではないということになる。もっとも、ヨーロッパ自身の自己解釈、あるいは丸山が整理したヨーロッパの特徴とされる「首尾一貫したドグマとしてのキリスト教」でさえ、キリスト教受容後のヨーロッパでも、ギリシャやローマ、あるいはゲルマンや北欧の神話が変わらずに親しまれてきたことを勘案すれば、実際のところは二重信仰性の実態があり続けてきたものと解釈する可能性さえあるといえる。

以上の議論を我々の文脈に即していえば、「選択や決断を基盤としたこのような「宗教」観を共有してはいない」ということは、「二重信仰」さらに「トリプルな信仰」も否定しきれないということになる。さらに『アジア神学講義』の 211 ページでは「仮にもある文化に福音が伝えられ受容され意義を保ち続けているならば、そこには必ず何らかの変容が認められるはずである」と述べる。もちろん「まったく宿主文化に埋没してその存在すら気づかれないようでは、そもそも文脈化の意義が失われてしまう」が、「「二重信仰」の文化を背景として考えるならば、それも優れて文脈化の一形態であるかもしれない」といい、山本七平の「日本教キリスト派」に言及し、それを「人間論型」の文脈化理解からすると、むしろ賞賛されるべき文脈化の成功例ということになるであろう」と評する。『アジア神学講義』のこの「二重信仰」から「日本教」のくだりのみを取り上げれば、この本も「トリプルな信仰」を否定していないとも読みうる。このことは同書の前の方で武田清子や森岡清美らの社会学者が、原型なり祖型がどこかにあるという前提があると述べる点を批判的に言及することにも通じる。『アジア神学講義』で紹介されたアジアの神学者たちの議論をニーチェによるパウロ以降のキリスト教批判と同じ論理構造を持つことを指摘する森本も、同様に、土着化される以前の「純粋なキリスト教」というものはどこにも存在しないと語る( 森本2004 p.7)。イエス当人も当時のご当地的なユダヤ教理解をしていたものであり( 田川 2004)、宗教がどれほど高度に論理的に鍛えられて普遍化を行っていっても、土着的な要素が皆無であることは不可能である。

また『反知性主義』(2015)で森本はウイルスの宿主との相互作用に喩えて次のようにいう。「それと同じように、宗教も伝播の過程で、その土地の文化に大きな影響をもたらしつつ、同時にみずからを変化させていく。宗教学的に言うと、これは「土着化」や「文脈化」と呼ばれるプロセスである。それが成功すればするほど、宗教はその土地に独自のものとなり、変容を遂げてゆくわけである」( 森本 2015 p.25)。しかし、神学は、文脈化を越える努力を求められる。『アジア神学講義』に戻るが、「神学はそれゆえ、自己の文脈を越えたより広い文脈に無関心であることはできない」( 森本 2004 p.9)。「本来的な意味でのテキストは、時にコンテキストを超越する「権威」をもち、おのずと受け手のコンテキストをある「審判」のもとに置くはずである」( 森本 2004 p.10)。そしてこの森本の書の全体は文脈化をこえる志向をより強く持ち続けつつ、文脈しようとしたアジアの神学者へも敬意も示すという矛盾した姿を、あえて示しているように思われる。否、むしろ、普遍化と文脈化という真逆のベクトルを両方とも必要な相補的なものと見なしたうえで、両者のサイクルを上手く回すことを主張していると解釈する方が整合性がある。森本は『アジア神学講義』で触れたアジア人の神学者たちの問題意識や意義は否定していないが、普遍化の不足と思える個所には随所で批判を加えており、一方でその目的は彼らアジア人神学者たちの問題意識がこのままでは埋もれてしまうという危機感に根差しているからである。

ただ本稿の最大の意図は、あくまでも普通の15~29歳のインフォーマントを、キリスト教主義学校に通った(通う)者、そうではないが何らかの当事者、当事者以外の三カテゴリーの人たちが、キリスト教主義学校の設立意図を推測させて、その結果の三カテゴリー間のズレを考察することで、キリスト教主義学校が日本で人気がある理由を探ることにある。要するにあくまでもターゲットはミッションの受け手である日本社会における一般人の意識であって、今少し述べさせてもらったような、送り手である聖職者や、聖職者の日々の研鑽の成果である神学や、日本国内でのキリスト教社会の実態は、直接の研究目的ではない。

もちろん我々の研究グループの今後の研究テーマではあるが、筆頭著者は近々定年退職するので、筆頭著者自身とは関係は薄くなる。

### 5.6 キリスト教主義学校という制度の自己目的化―本論攷の限界と今後の課題を見据えて

もう一つ考えるべきは、キリスト教主義学校という制度の自己目的化、いいかえるとキリスト教主義学校という制度が物象化し、当初の設立目的から自立して動いていくことである。結局これもマートンの機能の類型論で理解できる。

先ほどはマートンの着想を援用して、信者を増やすという狭義の布教・ミッションについて顕在機能と潜在機能とを分けて論じたが、そもそも2.1でみたキリスト教主義学校の設立目的においても、機能の類型論を併せみる必要性があろう。布教のみならず「使命」の自覚も含めた広い意味でのミッションあるいは徳育としてのキリスト教を広めることが、顕在機能としてある。しかしひとたび学校が設立されれば、その学校の維持・発展という組織論的な発想が第一の目的に転じ、それが理事会、教職員、同窓生等、関係者たちから意識される。それらが潜在機能となり、場合によって新たな顕在機能となり、制度の自己目的化が起こる。学校の維持・発展には入学希望者という潜在的な生徒・学生の評価が重要で、これらは競争率や入試の偏差値に典型的に示されるが、それら潜在的な生徒・学生の評価を高める最も大きな要因は、卒業生の進路の良さであろう。高等学校まででいえば進学先の良し悪しであろうし、大学・大学院であれば就職先の良し悪しである。情操教育など数字に直接現れない教育の良し悪しも生徒・学生の評価を高める大きな要因ではあるが、進路の良さに較べれば副次的なものに留まる可能性も否定はできない。特に男女共同参画社会の定着によって、主に女性においてだが情操教育によって良い結婚相手をみつけ、階層の上昇や再生産を図るという戦略の有効性が低下した。そのような現代において、ますます進学・就職状況が、受験対象校として選ばれる際の重要な決め手となっていく。旧来の例としては、例えば廣瀬つぎ子編 『雙葉小学校創立三十年記念誌　小百合』(1941) では、卒業生の動向を記すコーナー（「一回生消息」等）が設けられていたが、そこでは結婚相手の職業も記述されることがほぼ常であったし、子どもの在学学校も記されていた。「一回生の消息」の二番目に記された人は以下のようになっている。「○○様（△△）女子学習院御卒業後・・・御主人さまには南洋長官、拓務次官と官界に御活躍其の御内助ぶりも伺われ誠に御立派な奥様でいらつしやいます。御令息様は慶應に、御嬢様は母校女学校二年に御在学でございます」 （ 廣瀬 1941 p.32〔○○は人名、△△は県名。元々は匿名ではない〕）。今は男女平等のみならず個人情報保護の観点からも、このような記述はあり得ない。そうするとキリスト教主義学校の「キリスト教」の部分の比重は女性については従来男性より強く意識されたのに、男性並みにその意識は弱まらざるを得ないし、男性についても今まで以上に弱まるであろう。

井上順孝は、「戦前に宗教立の学校が設立された場合、その教育に、宗教的理念を生かそうとする傾向はかなり強かった」（ 井上ほか 1997 p.19）と記す一方で、「しかし、戦後は、そうした創立時の宗教的情熱は一般に薄れる傾向に」あるという。また「戦後は、一九五五年頃までは、宗教教育に熱心な場合も少なくなかったが、やがてそうでもなくなった」（ 井上ほか 1997 p.19）とも記す。確かにキリスト教主義学校の連盟のウェブサイトにおいて、クリスチャンの教員の比率が経年的に漸次減少している点を議論する情報が掲載されている。進学実績を向上させ、偏差値や競争率をアップさせるには、教員のクリスチャン条項を外すことはいうまでもなく、場合によってはキリスト教主義に共鳴する人材を教員に迎えることさえも断念し、受験指導や進路指導に長けた人材、そうでなくても授業の巧みな人材を雇用した方が有利になる。しかも競争率が上がれば、定員も増やしやすくなるし、大学院博士課程・大学から中高の一貫（系列）校の場合には、アカデミック・ポストの恒常的不足のなか、大学院生の進路の面からも機会があれば定員は増やせという圧力は常時あると考えられる。定員が増えていけばそれに合わせて教員増となり、アカデミック・ポスト不足を多少なりとも緩和する。漸次定員が増えていくなかで、設立当初のキリスト教主義に強く共鳴した教員の比率が下がるのは当然である。

先に言及した 佐藤 (2006 p.17) の挙げた 1926~1930 年の例は、卒業生の 50% 程度が入信しているが、往時は定員も今よりは遥かに少なく、教員のクリスチャン率も現在より高かったことが推察される。この辺りは今後史料や文献を通じて明らかにしていきたい。例えば 2018 年 10 月にカトリック社会問題研究所が主催した、少子化・公立志向・教育改革、非信徒教員の増加の状況を踏まえて行われたセミナーで、日本カトリック学校連盟事務局長（当時）の品田典子は、信徒教員の減少については、特に信徒教員が非信徒教員から IS（イスラム国）ならぬ CS (Christian State) 呼ばわりされることが多い一方で、カトリックに理解を示す非信徒教員も多くいる現状から、非信徒教員との連携体制の構築が肝要という（「 少子化、信徒教員の減少、迫る教育改革　カトリック学校の現状と将来 (2018 年 11 月 29 日 23 時 05 分） 」最終閲覧日 2022 年 10 月 9 日）。

またこれはプロテスタントの方であるが、我々のインタビューに答えてくれた、小中高とキリスト教主義の学校に通った学生Bさんは、次のように答えている（調査日 2019 年 1 月 22 日）。

「後藤:それから、先生の中でクリスチャンは、小学校と中高で違うかもしれないんですけど、自分がクリスチャンですと言うかも色々だと思うんですけど、雰囲気としては小学校の時はどのくらいクリスチャンの先生がいましたか?

B さん:小学校のときは、結構いたと思います。礼拝のときにお話しする先生が小学校の先生とかもいらっしゃって、その人たちは大体クリスチャンだったと思うので。小学校はたくさんいました。でも、中高だと人数も増えるので、クリスチャンじゃない先生も普通にいらっしゃいました。

後藤:小学校はクリスチャンが多いということなんだけど、礼拝のときに話をするのは、宗教の先生とかじゃなくて、普通の先生がお話をするということ?

B さん:はい。

後藤:大体の先生がそこでお話をしてた。そうすると、小学時代のほとんどの先生がクリスチャンかどうかさておき、キリスト教について教えられるほどに、キリスト教の素養がほとんどの先生にあったということ?

B さん:はい、小学校の時はそうでした。

後藤:中高はそうでない人もそこそこ、結構いただろうなって感じで。じゃあ例えば親しい先生、歴史部の顧問の先生は違いましたか?

B さん:違いましたね。

後藤:クラス担任、学年によって違うと思うんですけど、大体クリスチャンじゃなかった?

B さん:大体違いましたね。中高はそんなにいなかったと思います。・・・礼拝も外部の方を呼んだりすることも多かったので。

後藤:えっ、そうなんですか?

B さん:はい、他の礼拝、教会の方が来てくださって、みたいな話が多かったので。

後藤:なるほど。そうすると聖書を教える先生以外は、あまりキリスト教徒かどうかは別として、キリスト教に詳しくはないと。キリスト教徒でも詳しくない人もいるけどね。

B さん:あまり、はい」。

要するに、小学校の教師はクリスチャン率が高かったと推測されるし、たとえそうではなくてもキリスト教についての知識が多かった ^26^ が、同じ系列であるのに中学になると、そういう教師が減ったという。進学実績の重要性の低い小学校では情操教育の比重が大きいので、キリスト教に親和的な教員を配し、一方、受験等の進路が評価に影響する中高ではそういう教員が減っているという実態が窺われる。つまりキリスト教主義学校の維持・発展が自己目的化するという組織論的な側面を考察すると、キリスト教主義学校の設置目的の推測として「キリスト教の信者を増やすこと」よりも若干ではあるが、「信者にならなくても、キリスト教の教えを守ることによって、救われる可能性のある人を増やすこと」が多い点を、先ほどはミッション・布教の土着化（いいかえると「徳育としてのキリスト教」、使命としてもミッションといった、改宗を特に迫らないキリスト教の定着も広義の布教と考えること）のレベルで捉えたが、そのことの妥当性は、競争率の高さの維持等、より実務的な要因も含めて吟味の余地があることになる。信徒を得る、シンパを確保する等の、直接的なミッションではない、与えられた使命の自覚、徳育としてのキリスト教を含めた広い意味でのミッションでさえ、副次的になる可能性も大いにあるからである。日本における徳育は前近代から仏教や儒教に対して期待されてきたものであり、戦前はそれらの伝統が教育勅語を中心とする修身による修養主義として定着してきた。戦後になると先の大戦の反省から、道徳教育に対する風当たりが強くなった。反面、高度経済成長や近代化のさらなる進展によるアノミー化していく社会の変動を背景に、徳育に対する需要自体は戦前と変わらずに存在していたものとするのであれば、キリスト教学校はそうした父兄の需要に応えたものとしてマーケティング的にも合理的である。そして戦前のクリスチャンの代表的人物である新渡戸稲造は『武士道』の執筆者であり、儒教道徳とキリスト教の近さをそこでは謳っていた。新渡戸の描く武士道は戦国時代までの武士ではなく、平和な時代となり朱子学を学び官僚化した武士をイメージしたものであり、他の身分に対して徳を示して統治することを理想とするモデルをキリスト教と結び付けている。新渡戸は内村鑑三とともに多くの信者を獲得し、南原繁や矢内原忠雄、大塚久雄ら戦後の東大を支える知識人を送り出した。丸山もその南原の弟子で、南原自身は宗教的に新渡戸、内村の弟子でもあるが、丸山もまた武士のエートスから近代化の要素たる「自然から作為への流れ」を読み取る際に荻生徂徠に着目し、儒学にキリスト教社会同様の「近代化していく社会」「社会契約され構築された制度という普遍性」を見出したことで、儒学とキリスト教の共通性を主張したといえる。

マートンの順機能、逆機能の用語は用いないが、本節で述べたような状況を井上順孝も捉えている。井上は当該著書出版の時期の 1997 年「現在」の宗教系の学校の宗教教育の現状を次のように記していた。「さて、宗教系の学校が宗教教育をどのように位置づけているかという点からすると、現在は大きく二つのタイプを見いだすことができる。一つは、宗教教育の実践を大きな目標のひとつとして教育がなされる場合であり、もう一つは単に教育事業に進出するというだけで、宗教教育の実を上げることはほとんど目標としていないものである。極端に言えば、後者においては、教育が一つの事業として成り立てばいいのであり、学業やスポーツ、その他の活動において、優秀な学生を集め、その学校の知名度が上がることが、むしろ大きな目的である。前者のタイプの宗教教育は、戦前に多かったが、戦後はしだいに後者のタイプが主流になっている」（ 井上ほか 1997 p.20）。そして「現在」宗教系の学校の率の高いのが高等教育であるとも付言する。「一九九三年における、全国の小学校、中学校、高校、短大、大学の総数（分校、夜間部を含む）、うち私立の数、それに一九九三年度における宗教教団関連の学校数を検討してみると、教育機関に占める私学の比率は、とくに高等教育において顕著である。その中で宗教系の学校の占める比率はきわめて高い」（ 井上ほか 1997 p.20~21）。この井上編集の著書から 25 年経ている現在、ますます「後者のタイプ」、すなわち「優秀な学生を集め、その学校の知名度が上がることが、むしろ大きな目的」である宗教系学校の増えていることは想像に難くない。

なお、コロナ禍の影響もあって、我々はキリスト教主義学校の幹部や聖書・キリスト教学の教員にまだインタビューできていない。またカトリックとプロテスタントの学校の差はウェブモニターの中で多数派ではないキリスト教主義学校通学経験者をさらに分割することになるので、まだ捉えられていない。さらに先述の「日本社会の「トリプル信者」を生み出す要素はいろいろあるだろうが、重要なのは中学校・高等学校の教育ではないかと思う」（ 井上ほか 2018 p.139）という先行研究での発言に本研究もまずは依拠したので、大学のみキリスト教主義学校に在籍した者と下から上がってきた者との違いも充分に調査できてはいない。これらの側面からの吟味は今後の課題としたい。

## 6. 本稿全体の結論

本稿は科研費挑戦的萌芽研究「キリスト教主義学校から見る日本人の寛容と洋化-ステークホルダーらの期待と文化資本」 （2018~2023年度） （課題番号:18K18590）のウェブモニター調査の一部（なおかつ最重要と我々が思う部分）の変数のクロス集計結果の分析報告であり、それの結果報告の具体的に肉付けに必要な限りでのインタビューでの発言やウェブ情報、文献資料からの引用を加えたものである。

この研究全体の研究目的は 1.2「本研究の目的」に記したように、日本のクリスチャンの数は日本の人口比で 0.8% しかいないにもかかわらず、キリスト教主義学校が日本の私立学校のなかで一定程度以上の定員比率を誇り、また人気を集める学校も多いのはなぜかを究明することである。

この全体の研究目的に即しつつウェブモニター調査の最終的な究明目的に移行するための作業仮説として、1.2 では、「キリスト教主義学校は何を目的として設立されている」と考えられているかというイメージの解明が、《 「日本の人口比で 0.8% 前後しかいない」という事実が、「キリスト教主義学校が日本の私立学校のなかで一定程度以上の定員比率を誇り、また人気を集める学校も多い」という状況と矛盾する状況》の解明に役立つ作業仮説であると考え、これを作業仮説1とした。

またもう一つの、この全体の研究目的に即しつつウェブモニター調査の作業仮説に移行するための作業仮説としては、これも 1.2 で既述のことではあるが、この全体の研究目的の《 「日本の人口比で 0.8% 前後しかいない」という事実は、「キリスト教主義学校が日本の私立学校のなかで一定程度以上の定員比率を誇り、また人気を集める学校も多い」という状況と矛盾する状況》である点に着目している。この人気の理由の解明には、キリスト教主義学校の生徒・学生イメージを、人びとがどのように捉えているかを見ることである。よってこれもこの全体の研究目的に適う作業仮説であると考えられる。これを作業仮説2とした。

ここで挙げた作業仮説1に対応させて、我々のウェブモニター調査では、キリスト教主義学校設立目的の推測を問う設問Q34を設けた。分析の際は、この設問の各選択肢ごとにその選択肢を MA ベースにおいて選んだ人を 1、選ばなかった人を 0 とする離散変数を作った。このそれぞれの選択肢による離散変数を従属変数として、キリスト教主義学校当事者性に関わる Q12 の組変数 Q12G を主な独立変数として、クロス集計を行った。この結果は 3.1 で概ね記した。

4.「調査結果のまとめと結論」で記したことをこの段落ではほぼ繰り返すが、Q34 の分析結果として、キリスト教主義学校に通った者等、キリスト教主義学校への関与度の高い者の方が、関与度の低い者に較べて、キリスト教主義学校の設置目的として信者の獲得を挙げる者の比率は、有意差がない（p値=0.13）ものの、少ない傾向にある。なお、有意差がなかった理由を本稿 2.1 では検出力不足（データ数の不足）によるものと考察した。

他方、「信者にならなくても、キリスト教の教えを守ることによって、救われる可能性のある人を増やすこと」を選ぶ者が、キリスト教主義学校に通ったことのある者の方に多い（カイ二乗<0.01）ことも分かった。また「信者の子弟の教育要求への対応」、「教会以外の、キリスト教に触れる敷居の低い舞台としての役割を果たすこと」を選ぶ者も、キリスト教主義学校に通った者等、キリスト教主義学校への関与度の高い者の方に多いことが分かった。なお、この「 「信者にならなくても、キリスト教の教えを守ることによって、救われる可能性のある人を増やすこと」を選ぶ者が、キリスト教主義学校に通ったことのある者の方に多い（カイ二乗<0.01）こと」については、後述する作業仮説 3 のトリプルな信仰にも関連する。

作業仮説 1 についてここで小括するが、作業仮説1は「キリスト教主義学校は何を目的として設立されている」と想定されるかということの解明が、《 「日本の人口比で 0.8% 前後しかいない」という事実が、「キリスト教主義学校が日本の私立学校のなかで一定程度以上の定員比率を誇り、また人気を集める学校も多い」という状況と矛盾する状況》の解明に役立つ作業仮説であるというものであった。これとこの前段落の結果と照らし合わせると、（少なくても狭義の）布教という目的を日本のキリスト教主義学校は放棄しているとまではいえないまでも、ほとんど強く有していないように受け取られるから、「日本の人口比で0.8%前後しかいない」という事実と、「一定程度以上の定員比率を誇り、また人気を集める学校も多い」という事実とが矛盾しないことになっているといえる。

つぎにここで挙げた作業仮説2に対応させて、我々のウェブモニター調査では、キリスト教主義学校の生徒・学生イメージについて Q29 で聞いた。分析方法は、Q34 と基本的に同様で、この設問の各選択肢ごとにその選択肢を MA ベースにおいて選んだ人を 1、選ばなかった人を 0 とする離散変数を作り、このそれぞれの選択肢による離散変数を従属変数として、キリスト教主義学校当事者性に関わる Q12 の組変数Q12G を主な独立変数として、クロス集計を行うことにした。この結果は 3.2 で記した。

これも作業仮説 1 の場合と同様、4.「調査結果のまとめと結論」で記したことと、この段落とこの段落の次の段落はほぼ重複するが、 井上ほか (2018) はキリスト教主義学校の女子は「きれい、金持ち、キリスト」の 3K であるということを強調していて、 佐藤 (2006) でも同様の記載はあったので、その点をまず吟味した。金持ちであるイメージについてはある程度妥当した。しかしきれいという点はまったく妥当しないことが分かった。3Kのうちキリストは同義反復的であるので措くとすると、1 勝 1 敗である。また 井上ほか (2018) ではキリスト教主義学校では愛と奉仕の精神を教育目標に掲げるところが多いと、実際の教育効果の検証なしに、ウェブページの分析をして述べていた。したがって「他人を思いやる心が豊かである」を Q29 の選択肢に入れた。これは Q12G とクロス集計すると、キリスト教主義学校当事者の方が有意にこれを選ぶ者が多かった。しかし実際に通ったことのある者よりも当事者性の低い、緩やかな当事者の方がこれを選ぶ者が多かった。その点で、これは「他人を思いやる心」を涵養させうるだろうという家族等周囲の期待がその数字に表れていると考えられる。またこの Q29 で「該当なし /答えたくない」を除き最も選ばれたのは「外国語が得意である」である。女性皇族のキリスト教主義学校関与の是非を問う Q11G とクロス集計すると、キリスト教主義学校への関与を是とする者ほど、「外国語が得意である」を選んでいることが分かった。また Q12G とクロス集計すると、キリスト教主義学校関与度と関連はあったが、通学経験者よりは緩やかな当事者の方に「外国語が得意である」を選ぶ人が多かった。また「話し上手である」を選んだ人選ばなかった人をクロス集計すると、やはり通学経験者よりは緩やかな当事者の方にこの選択肢を選ぶ人が多かった。

またこの Q29 でその次に選ばれたのは、「教養が豊かである」で、これも女性皇族のキリスト教主義学校関与の是非を問う Q11G とクロス集計すると女性皇族のキリスト教主義学校への関与を「望ましい」と考える者は、「教養が豊かである」を選ぶ人が多かった。またこれを Q12G とクロス集計すると、キリスト教主義学校にかかわりの高い者ほど、キリスト教主義学校出身者を「教養が豊かである」と自認していることも分かった。

これらの結果から、女性皇族のキリスト教主義学校関与を是と捉える層からの皇室外交への期待がうかがわれる。また「外国語が得意である」と「教養が豊かである」が、Q29 で「該当なし /答えたくない」を除き一番目、二番目に選ばれ、「他人を思いやる心が豊かである」が六番目であることを考察すると、キリスト教主義学校の意義を、宗教教育をはじめとする情操の涵養のレベルで捉えるよりも、外国語や教養などのより実利レベルで捉える人の方が多いということになる。

そこで作業仮説2に関する小括であるが、作業仮説 2 は「キリスト教主義学校の生徒・学生のイメージを、キリスト教主義学校の当事者を含めた世間の人びとはどのように考えているか」ということの解明が、《 「日本の人口比で 0.8% 前後しかいない」が、「キリスト教主義学校が日本の私立学校のなかで一定程度以上の定員比率を誇り、また人気を集める学校も多い」という状況》の解明に役立つというものであった。これとこの前々段落と前段落の結果と照らし合わせると、少なくとも東京・神奈川在住の若年層においてキリスト教主義学校が、 井上ほか (2018) のいうような3Kというブランドイメージが、少なくも「きれい」ということについては定着しているわけではない可能性が分かった（有意差なしは、有意差ありとは違って、統計学的にも証明が難しいといわれるが、「きれい」については実数そのものさえも少ない）。しかし「外国語が得意である」と「教養が豊かである」が皇族女子のキリスト教主義学校への関与を是とする人から選ばれていることから、やんごとなきところに親和性があるという意味でのブランドイメージはあるといえよう。また「外国語が得意である」「他人を思いやる心が豊かである」「話し上手である」という選択肢は、キリスト教主義学校関与度と有意な関連はあったものの、通学経験者よりは緩やかな当事者の方にこれを選ぶ者が多かった。このことはキリスト教主義学校の生徒・学生イメージのうちの肯定的なもののいくつかは、通学経験者よりは緩やかな当事者の方に支持されているイメージであるといえるであろう。したがって作業仮説に合わせて考えると、キリスト教主義学校の肯定的イメージが《 「日本の人口比で 0.8% 前後しかいない」が、「キリスト教主義学校が日本の私立学校のなかで一定程度以上の定員比率を誇り、また人気を集める学校も多い」という状況》をもたらしているといえるが、それら肯定的イメージのいくつかは、キリスト教主義学校の通学経験者よりは緩やかな当事者から支持されているということが分かった。つまり現実よりはイ メージ先行の部分もあるということである。

また「外国語が得意である」と「教養が豊かである」が、Q29 で「該当なし /答えたくない」を除き一番目、二番目に選ばれ、「他人を思いやる心が豊かである」が六番目であることを考察すると、キリスト教主義学校の意義を、宗教教育をはじめとする情操の涵養のレベルで捉えるよりも、外国語や教養などのより実利レベルで捉える人が多いということになる。その点でも作業仮説1での分析結果で申し上げた、布教という目的を日本のキリスト教主義学校はほとんど強く有していないと周囲から受け取られるから、「日本の人口比で 0.8% 前後しかいない」という事実と、「一定程度以上の定員比率を誇り、また人気を集める学校も多い」という事実とが矛盾しないということを、補強的に裏付ける結果であるともいえる。

ただ Q34 について先にふれたように、「信者にならなくても、キリスト教の教えを守ることによって、救われる可能性のある人を増やすこと」を選ぶ者が、キリスト教主義学校に通ったことのある者の方に多い。この問題はトリプルな信仰に関する作業仮説 3 に通じる。

1.4 で論じたように、作業仮説 1 や作業仮説2での議論は、「 「日本のクリスチャンの数は日本の人口比で0.8% 前後しかいないにもかかわらず、キリスト教主義学校が日本の私立学校のなかで一定程度以上の定員比率を誇り、また人気を集める学校も多いのはなぜかを究明すること」という命題の後半部分、「キリスト教主義学校が日本の私立学校のなかで一定程度以上の定員比率を誇り、また人気を集める学校も多いのはなぜかを究明すること」に焦点を当てていた。他方、もしもトリプルな信仰が成立するとすれば、「日本のクリスチャンの数は日本の人口比で 0.8% 前後しかいないにもかかわらず」という部分を吟味することになる。よってトリプルな信仰についての意識がキリスト教主義学校通学経験者の方が、それ以外よりも多いかどうかを、作業仮説3とし、主にこれは本稿 3.3 で論じた。

キリスト教主義学校の通学経験者はそうでない者より、原家族と共にキリスト教以外の仏教や神道の宗教的行事に参加してきた。この結果は 井上ほか (2000) でカトリック系の高校生が、神道系高校の生徒以上に、初詣をするという過去の知見も我々と同様の方向性を示しているといえる。したがってキリスト教主義学校の通学経験者は、キリスト教にまつわる行事・習俗であるか否かを問わず宗教あるいは宗教にまつわる習俗を尊ぶ環境に育ってきた者が多いといえる。そのことはトリプルな信仰の土壌があるということをある意味で裏づける。「ある意味」と留保をつけたのは、習俗が形式のみであるという批判もありうるからである。ただし 井上ほか (2018) の 佐藤 (2006) への言及部分に関連づけて 1.3.2 にて申し上げたように、習俗、形式が内面化すると信仰として精神化しうる。したがってトリプルな信仰に結びつきうる土壌がある。確かにキリスト教主義学校の教育効果としてトリプルな信仰があるかどうかはここでは分からないが、トリプルな信仰への期待、いいかえると宗教教育を通じての情操の涵養への期待のなかで、キリスト教主義学校の通学経験を有する回答者自身が、両親等の保証人から、キリスト教主義学校に送り込まれた可能性を示す結果といえる。つまり同志社でいわれたとCさんがいう、「徳育としてのキリスト教」という発言とも整合する。また作業仮説1の箇所で申し上げた、Q34 の「信者にならなくても、キリスト教の教えを守ることによって、救われる可能性のある人を増やすこと」を選ぶ者が、キリスト教主義学校に通ったことのある者の方に多いということは、単に情操教育の対象として以上に、トリプルな信仰に通じる場として実際にキリスト教主義学校が機能していると推測されていることを裏づける。

さらにそのことを裏づけるものが Q49 の結果である、Q49 は「以下の宗教において、入信していなくても教えを守ることによって、救済やご利益（ごりやく）が得られると思いますか。当てはまるものをそれぞれ選んでください」という設問で神道と世界三大普遍宗教合わせて 4 宗教について同じ尺度で聞いている。キリスト教主義学校通学経験者の方がキリスト教について「そう思う」人が多い。　

以上の結果から作業仮説3は裏付けられた。

井上ほか (2018) における郭の議論からすると、既成仏教や神社神道においては、入信というプロセスを経ないでも、それらの宗教の信仰者といえる。それと同様、キリスト教も、一般の日本人の意識からすると、もしも洗礼というプロセスが不要であれば、仏教や神道と同様、三つ目の信仰の対象としうるということになる。要するに、その場合には「0.8%」というのは見かけ上の数値に過ぎないということになる。洗礼というプロセスは経ないもののキリスト教主義学校通学経験者の方がトリプルな信仰に親和的なことが分かった。

これによって科研費研究全体の研究目的「 「日本のクリスチャンの数は日本の人口比で 0.8% 前後しかいないにもかかわらず、キリスト教主義学校が日本の私立学校のなかで一定程度以上の定員比率を誇り、また人気を集める学校も多いのはなぜかを究明すること」という命題のうち「日本のクリスチャンの数は日本の人口比で 0.8% 前後しかいない」部分が、じつは見かけ上低い数値であるにすぎない可能性が示唆される。もちろん信仰を聞く質問において、今回の回答者の 2.7% が既成キリスト教 (Q1-9)と答えていて全国平均の 0.8% の 3 倍以上あるとはいえ、クリスチャン自身がこのように考えているという保証はない。しかし「見かけ上低い数値であるにすぎない」と一般の人から想定され、特にキリスト教主義学校通学経験者からそのように思われているということは、いえる。

さらにこのことと関連してキリスト教主義学校のウェブ調査等の検討から、キリスト教主義学校の教育目的を布教という狭い意味のミッションよりは、神に与えられた使命としてのミッションを果たすという広い意味で、当事者たちから理解されていることが分かった。またこの「信者にならなくても、キリスト教の教えを守ることによって、救われる可能性のある人を増やすこと」を選ぶ者が、キリスト教主義学校に通ったことのある者の方に多いということは、洗礼と非洗礼で一線を画し、首尾一貫性を重んじるという、従来いだかれてきた日本のキリスト教像とは異なった見方を、キリスト教主義学校当事者たちがとっている可能性もあるということにもなる。これは先行研究の 井上ほか (2018) のいう「トリプルな信仰」としてのキリスト教という考え方に照応する結果であるともいえる。もちろん教育基本法の縛りの影響も勘案する必要があり、今回そこにあまり言及出来てはいない。今後、キリスト教主義学校の経営陣へのインタビューをする中で、その面も含めて、より明確にしていきたい。

前章でも触れた ICU 元副学長で東京女子大学学長を現在務める森本あんりは、『不寛容論―アメリカが生んだ「共存」の哲学』でロジャー・ウィリアムズ (1603~1683) の寛容論を高く評価し、彼について多くの章を割いて分析・紹介する。ケンブリッジ大学を出た後に裕福な家庭のお抱え牧師だったウィリアムズは、先住民の権利等、当時としては過激な主張をしてマサチューセッツ植民地から追放され、今のロードアイランド州に赴き、そこの先住民を対等な立場の人間として扱い、『アメリカ語理解の鍵』という本も著した。ここでいう「アメリカ語」とは先住民の言語の意味である（ 森本 2020 p.129）。そこで扱われている例文は先住民の生活ぶりを内側から観察して得た内容になっている。このように非常に先住民からの信頼の厚かったウィリアムズではあったが、形だけの宣教を彼は拒否する。「自分がその気になれば、何千という先住民を、いや彼らの国全体を、キリスト教へと回心させることだってできる、と彼は豪語している。先住民の間で彼が得ていた信頼の厚さを考えれば、それも「から威張り」とは言えないだろう。けれども彼は、洗礼という「儀式」で外面的なキリスト教徒を作ることには反対なのである。むしろそれは、反キリストの業である。外から仕向けられた回心は、真の信仰を伴わない。ウィリアムズにとって、伝道とは「回心者の獲得」ではない。先住民であろうとイギリス人であろうと、ただ神の恵みによって生まれ変わることを求めるだけである。信仰は内心の自由からのみ生まれるからである」（ 森本 2020 p.134~135）。ウィリアムズは牧師として受けた教育と論争経験から、古代から中世に至るまでの正統と異端、寛容と不寛容に関する論点について精通していた。ウィリアムズはこれらの知見を用いつつ、内心の自由なくして真の信仰なしという徹底的な不寛容ぶりを梃子にして先住民への寛容を実現できた。ウィリアムズの生き様は、どこまでも寛容であるべきと考えても不寛容の要素を全て切り捨てることはできない事例であるとも取れるが、不寛容性を活用することでみずからの寛容を磨き上げることができる、という解釈することもできる。

このようなウィリアムズの宣教と洗礼についての議論と、Q34 の「信者にならなくても、キリスト教の教えを守ることによって、救われる可能性のある人を増やすこと」を選ぶ者が、キリスト教主義学校に通ったことのある者の方に多いというとは照応するのではないだろうかという思いを胸に、本稿を閉じることとしたいじることとしたいが、その前に、今年度限りで学問の世界から定年で引退する筆頭著者以外の、共著者のための今後の課題を付記しておきたい。

本稿 5.5 で少しだけ触れた森本の『異端の時代』でのジェームズに論及する箇所の少しあとの箇所で、森本は「プロテスタント病」という言葉をもちだす。

「先の（「置かれた場所で咲きなさい」という）尊い助言はカトリックのシスターによるものだが、時代はむしろプロテスタント化している。というより、個々人が異議申し立て（プロテスト）を続け、際限なく分裂を繰り返してゆく「プロテスタント病」にかかっている、と言わねばならない」（ 森本 2018 p.201）。この病の典型は「「意志力の崇拝」つまり「やればできる」と信じたがる精神である」という。そして選ぶことが強制された現代は、異端がデフォルトである時代になっている時代であり、「正統の消失とともに、異端もまたホームレスと化し」（ 森本 2018 p.202）ているという。その状況の「典型的な体現者」（ 森本 2018 p.203）の一人がジェームズであるともいう。またこれらは R・N・ベラーのいう「宗教と化した個人主義」（ 森本 2018 p.216）であるともいう。

この「個人主義的宗教の特徴」として、権威とされているものを批判する際、宗教的高揚感をもつが、場合によって「御上たたき」は「不健全でいびつ」となり、その行き着く先はポピュリズムであるという。これは宗教に限らず、イデオロギーや未来の夢といった「信仰システム」の崩壊、信憑性構造の揺らぎが背景にあり、「みずから新たな正統を担おうとする覚悟とある異端だけが、真の異端」（ 森本 2018 p.239）と述べ、真の異端によってのみ正統の復権が可能であるという形で『異端の時代』を〆る。

この『異端の時代』では「寛容」という言葉を意図的に使わなかったという森本は、その後寛容論は不寛容を内在するという問題意識から『不寛容論』(2020)を著す。要するに寛容の敵、つまり己の内集団・社会的属性・イデオロギーに固執して、外集団やその社会的属性・他人の思想を排斥するグループや個人に対して寛容を許容するかという問いを、背景にもつ。『不寛容論』は日本人の不寛容性の問題をほぼ扱わないが、同書の後に書かれた『東洋経済オンライン』の記事「「日本人は多神教だから寛容」通説は本当なのか」で、一見寛容に見える日本人が実は不寛容であると森本は述べる。前章で触れたジェームズと西田の議論を振り返りつつ、トリプルな信仰の寛容性をこの森本の議論を用いて評価すれば、日本人の寛容性の根幹は個人主義的宗教による寛容性に他ならないことになるが、その寛容性の問題点は確固たる不寛容性を内在しないことに起因してしまうこととなる。そして、確固たる不寛容性を内在しない寛容性は、真に寛容性があるとは言えないのではないか、と森本は問いかけるのである。この観点からしても、確固たる不寛容性を内在することで機能的に最も寛容であることに成功したウィリアムズを森本は高く評価する。寛容のためにも不寛容が必要、というパラドキシカルな命題に我々は一瞬面食らうが、理詰めで考えれば説得力があるものであり、日本の価値として寛容性を誇ることを目指すのであれば、この論点から逃れることは許されない。

またこの本の8年前の著書である『アメリカ的理念の身体』（創文社、2012年）の「あとがき」で、森本はつぎのように述べる。「ある学会シンポジウムでの質疑である。「いかなる社会にも絶対的寛容は不可能である」と論じた時、フロアから「絶対的寛容」を契約条件とすれば、絶対的に寛容な社会ができるのではないか、という質問が出た。その時は即答できなかったが、そのような契約は「絶対的な」形容矛盾に陥らずを得ない。なぜなら、その社会は「絶対的寛容」を支持する人しか参加できないわけであるから、「相対的寛容」を支持する人は排除され、非常に不寛容な社会を結果することになるからである」（ 森本 2012 p.272）。

日本の価値として寛容性を誇ることを目指すのであれば、森本の意見に対する是非は一旦横に置いても、この論点から逃れることは許されない。

本研究の研究グループの研究目的は、これらの森本理論を社会調査による実証で批判的に継受することである。要するに前々々段の「寛容論は不寛容を内在するという問題」の前提となる、寛容の対象と対象にならないものの線引きの問題等を考究することによって、森本理論の精緻化、発展を図りたい。

## データ可用性

### 基礎データ

Figshare:キリスト教主義学校に関する調査_ローデータ20200324.xlsx. https://doi.org/10.6084/m9.figshare.21366417.v1 ( Goto, 2022a)

上記プロジェクトには下記の基礎データが含まれている。

・キリスト教主義学校に関する調査_ローデータ20200324.xlsx（ウェブモニター調査の回答）

### 拡張データ

Figshare:キリスト教主義学校に関する調査　調査票画面.pdf. https://doi.org/10.6084/m9.figshare.21367356.v1 ( Goto, 2022b)

上記プロジェクトには下記の拡張データが含まれている。

・キリスト教主義学校に関する調査　調査票画面.pdf（本論文で扱われたウェブモニター調査の調査票）

Figshare:キリスト教主義学校調査票画面についての補足説明.txt. https://doi.org/10.6084/m9.figshare.21367632.v1 ( Goto, 2022c)

上記プロジェクトには下記の拡張データが含まれている。

・キリスト教主義学校調査票画面についての補足説明.txt（調査票の補足説明）

Figshare:キリスト教主義学校　インタビュー調査　調査依頼書.docx. https://doi.org/10.6084/m9.figshare.21367776.v1 ( Goto, 2022d)

上記プロジェクトには下記の拡張データが含まれている。

キリスト教主義学校　インタビュー調査　調査依頼書.docx（インタビュー調査の依頼書）

以上のデータは、クリエイティブ・コモンズ表示 4.0 国際 Creative Commons Attribution 4.0 International license (CC-BY 4.0) の条件下で利用することができる。

なお、本論文のインタビューの回答は、本論文に含まれている部分を除いて、公開することができない。インタビューの内容が調査対象者の私的な情報や対象者を特定できる情報、ライフストーリーや思想・信条上の問題など対象者に不利益を生じうる情報を多く含むために、データを非公開とすることが倫理審査に基づく同意書に書かれていることが、その理由である。同分野の研究者や査読者がデータの閲覧を希望する場合には、データの利用目的と方法を明記の上、責任著者後藤宛て (ygoto@slis.tsukuba.ac.jp) に連絡して頂きたい。調査対象者と逐次相談の上、開示可能な箇所を開示する。

## 謝辞

髙井啓介先生（関東学院大学）には、調査票のキリスト教にかかわる部分を事前にご校閲いただく等、諸般にわたりご教示賜りました。ここに記して深く感謝の意を伝えたいと思います。また、本稿にそのインタビュー記録の一部を引用させていただいた A さん、B さん、C さんはじめ、我々のインタビューに応じて下さり、貴重な話を提供してくださった方々にも、感謝いたします。

## Footnotes

^1^
栄光学園中学・高等学校は以前から神父を兼ねる教諭が同じイエズス会の上智大学に転出するケースはあった。例えばヨゼフ・ピタウ神父（のちの大司教）は若い頃栄光学園中学の教諭を務め、そのあとハーバードで学位を取り上智大学で教え、学長も務めた。もっとも 2016 年に学校法人栄光学園が同じイエズス会の六甲学院、広島学院、上智福岡中学校・高等学校と共に上智学院と法人統合したので、現在では上智大学と同一学校法人の下にある中学・高校となっている。

^2^
例えば我々の調査では Q31 で「あなたの日本のキリスト教主義学校のイメージを形づくった学校の種類」を SA で性別と課程を組み合わせた 9 選択肢から一択で聞いたが、「特にどれとはっきりイメージしていない」を除き、最も多いのは「中学校高校女子校」 17.8% であり、大学ではない。他方、「中学校高校男子校」 4.4% 、「中学校高校共学校」 11.3% ゆえ、中学校高校の限りではイメージとしての女子バイアスはある。しかし大学については、現代日本で男子大学はないという現実の非対称性の問題もあるが、「女子大学」 8.0% に対して「大学・共学」 14.7% で、ここでは女性バイアスもない。

^3^
本科研費研究交付年の 2018 年時点では「現代日本における宗教教育の実証的研究」 (1998~1999；研究代表者井上順孝）で行われた研究会やシンポジウムについてのウェブ情報が多くあって、本研究のウェブ調査の調査票を作るに際して大いに参考にした。ここもその一つである。ただし 2022 年度現在、この 1998~1999 の共同研究参加者の多くが退職されたためか、それら当時閲覧したウェブサイトの大部分が閉じられてしまっている。よってこの研究の報告書（井上順孝（研究代表者）科研費報告書『「現代日本における宗教教育の実証的研究」 (1998~1999) 報告書』 (2000、國學院大學）を 1. で記したように借り出し閲覧したが、ウェブに載っていた多くの情報はここには載っていない。閲覧時にログを保管しそこなった本稿筆頭筆者の怠慢のなせる業である。したがって、ここでは研究名のみに言及し、頁数や URL には言及しない。

^4^
ただこの現象は日本だけというのも早計である。WHAM! の Last Christmas 等、すでにスタンダードと化している洋楽の多くもいわゆるアガペーではなくエロースを扱っている。

^5^
佐藤卓己は京大教育学部に転出する前、日文研助教授として井上の後輩同僚であった。

^6^
ヒンズー教の牛、イスラム教の豚、仏教の僧侶の肉類全般のように、食のタブーは多くの宗教につきものである。

^7^
もっとも以下の記事公開時点で ICU 教授で副学長も務めた牧師の森本あんり（現、東京女子大学学長）は、日本人の寛容性は限定付きのものであるという。そもそも無宗教的で、宗教に無関心故、他者の宗教に寛容ではある。他人事である限り寛容であるが、「寛容の問いが自分自身に及び、深刻な利害が身の回りにひた寄せてくると」、容易に不寛容に転化しうる、という（森本あんり「 「日本人は多神教だから寛容」通説は本当なのか　統計調査をもとに考する「無寛容の正体」 」. 東洋経済オンライン）。このような森本の指摘にも、本研究の限界として、留意が必要である。このネット記事の元となったと称する著書で森本は次のように言う。「日本人は、クリスマスとお正月を一緒に祝い、生まれたときにはお宮参りをし、結婚式を教会で挙げ、葬式は寺に依頼する。だから宗教に寛容だ、というのが通説だが、こうして見る限り、どうやらそれはうわべだけの話のようである」（ 森本 2020 p.6）。一方この著書で森本は「本書が「中世的」と表現してきた寛容理解は、ごく大ざっぱに言うと、寛容論の分野で「伝統的な寛容」と呼ばれるものにあたる。この意味での寛容は、相手をしぶしぶ認めることである。相手を是認せず、その思想や行為に否定的であり続け、できれば禁止したり抑圧したりしたりしたいが、そうもいかないので、しかたなくその存在を認める、という態度である」（ 森本 2020 p.278）。それに対して最近の学説は、相手を承認する「肯定的寛容」「強い寛容」「認知としての寛容」「水平的寛容」であるという。しかし森本は「それらの主張もよくわかるのだが、ふりかえって自分の足元を見れば、それが常に可能というわけではないだろう」（ 森本 2020 p.279）とも述べて、「肯定的寛容」等の現代的な寛容がかえって反動としての「リベラル疲れ」をもたらす懸念も表明する。さらに現代的な寛容は「自分の信念への妥協を強いる」（ 森本 2020 p.280~281）可能性があるともいう。「相手にどういう態度で接するかだけでなく、内心で相手をどう評価するかまで寛容であれと要求するのは、潜在的にはとても不寛容である」（ 森本 2020 p.275）。

^8^
しかも南原の師である内村はまだ禁教が解けて日の浅かった日本の既存のプロテスタント教会からさえ、不敬事件、再臨運動、それぞれの折に、厳しく批判されてきた（ 若松 2018）。

^9^
読者モデルの掲載される誌面は私服紹介のコーナーが多く、2000 年代のファッション誌は今と違いプチプラコーデの記事が稀であったことからも、たとえ金持ちではなくても、基本的に少なくとも金持ちを装うことも、きれいと同時に必要とされたはずである。

^10^
注 (3) と同様。なお、「現代日本における宗教教育の実証的研究」（1998~1999；研究代表者井上順孝）より前の成果ではあるが、 井上ほか (1997) において、田口めぐみが「中等教育における宗教教育の意義」という論文で、宗教系の中等教育機関で行われる宗教教育は、宗教への関心を高める等、宗教教育としての効果はあるが、その学校の宗教を信じさせるという宗派教育としては有効ではないと述べた上で、次のように書く。「ただし、自分が通った学校がもつ宗教には、多少の親近感をもつようだ。仏教系の学校に通った学生は、日本人の宗教は仏教であると答えた者が他の宗教系に通った学生より若干多かった。キリスト教系の学校に通った学生は、「キリスト教をテーマにした小説」には他の宗教系の学校に通った学生よりも高い関心を寄せている。」（ 井上ほか 1997 p.225）。これらの記述はある意味、その宗教のシンパを形成していると見ることはできる。ただし、それを目的としているというよりは結果的にそうなっているということではあるが。

^11^
ここも注 (3) と同様。一方、 井上ほか (1997) において井上順孝は教育基本法の国公立学校での宗教教育の制約を指摘したうえで、「他方、宗教に関する寛容の態度及び宗教の社会生活上における地位は尊重され、また、私立学校においては、軍国主義的教説以外であれば、すべての宗教教育を自由に行えることとなった」（ 井上ほか 1997 p.11）と記す。さらに「宗教教育は、通常、三つのカテゴリーに分けて考えられている。第一は、特定の立場にたった宗教教育、第二は、宗教知識教育、第三は宗教的情操教育である。私立学校においては、児童生徒の信教の自由を認めた上でなら、この三つとも可能である」 （ 井上ほか 1997 p.19）とも記される。

^12^
ただキリスト教主義学校の教職員で自分自身や家族の通学歴等ない者をこの Q12 では漏らしてしまった点は、今後の検討課題である。

^13^
属性項目なので SA で聞いたが、トリプルな信仰の有無という本研究のテーマの性格上、MA と SA の併用もありえた点は、調査票作成上の反省点である。

^14^
本論では、キリスト教主義学校としてカトリックとプロテスタントを分けずに扱っているが、我々の共著者の一人は、近日まで、カトリックの中高一貫校とプロテスタントの中高一貫校にそれぞれ別の娘が通っていた。その共著者から、自分のような弱い当事者の視点からは、カトリック学校とプロテスタント学校には、「ミッション」のとらえ方、その「ミッション」を達成するための方策には違いがあると見受けられる、との意見が示された。プロテスタントとカトリックの「ミッション」概念の違いを視野に入れることは、今後の課題になる。

^15^
キリスト教主義学校以外も含めた学校法人全般においては、そういう疑念をもたれる実例が絶対にないとはいえないが、そうすると理事長には背任の疑いがもたれ、学校法人自体も私学助成金の大幅減額等、文部科学省から大きなペナルティを受ける可能性を伴うことは 2021 年新聞報道等を賑わせた事例からも明らかである。

^16^
なおプロテスタントの学校は佐藤のいうようにキリスト教伝道団体から独立しているが、カトリックの場合、法律上宗教法人と学校法人は別法人であるという点は措いて、それぞれの修道会が事実上系列の学校法人を監督している。したがってプロテスタントの場合ほどにはミッション・スクールでは「ない」ということを強調するモチベーションは、カトリックの場合は存在しないと推察できる。

^17^
ここの本文で「等」というのは 井上ほか (2018) で直接言及はされていないが、キリスト教主義学校卒業生以上に、一高校長（兼東大教授）新渡戸とその友人内村が、一高、東大に進む政財官学界の有力者の卵をプロテスタントに改宗させた部分も大きいはずだからである。新渡戸自身、南部藩の上級藩士の家柄で札幌農学校入学前から階層の高い人間である。内村も士族である。

^18^
なお 佐藤 (2006) は、ミッション・スクール的なリベラル・アーツは、それを学ぶことによって人を自由にするもの（ 佐藤 2006 p.147）で、他方、竹内洋が対象とするような旧制高校の修養主義的な教養主義はむしろその対極にある（ 佐藤 2006 p.156）と論じているが、新渡戸自身、一高というトップの旧制高校の校長と東大教授を務めた人物でもあり、なおかつ代表作の一つに『武士道』という著書もあるので、対比する部分と同時に連続する部分もあるはずで、その点の検討は今後の課題となる。しかし竹内のいう教養主義はデカンショマルクスという大陸合理論、ドイツ観念論と唯物論を中心にしたもので、英米の教養を身につけた新渡戸とは異質な部分も多い。新渡戸における教養とは何かを問うことは、リベラル・アーツ的な教養と修養主義的な教養との連続性と異質性を見る手がかりになると考えられる。

^19^
15~29歳を対象とした調査のため、結婚による階層再生産戦略が検出できていない可能性がある。結婚による階層再生産戦略については、より高い年齢層を対象とした調査が必要だと考える。

^20^
先述のように、井上はファッション誌の読モ（読者モデル）にキリスト教主義学校出身が多いことを数え上げていたが、読モ起用率の高さが若年層向き女性ファッション誌の売れ行き不振や休刊を招いた一因ともされる。読モレベルの容姿の女性の着こなしなら今では無料で SNS で閲覧できるからである。それどころか最近は SNS のインフルエンサーが女性ファッション誌の誌面に登場したり、専属モデルに抜擢されたりすることも多いし、男性向けのグラビア誌でもインフルエンサーの起用率は高い。SNS で人気を博するのに、通常、通学している学校は関係ないので、キリスト教主義学校の通学者が特にそこで目立つわけではない。現代の若い世代は、そのような情報環境に生きているので、このような結果になっている可能性もあると推測される。

^21^
波多野は『基督教の起源』にてまずユダヤ教の特質を律法にみる。「ユダヤ教の基礎は「律法（おきて） 」である。ユダヤ人は「律法」において神の聖なる意志を見た。真の宗教と偽の宗教と、また神の選民と他のすべての民族（異邦人）とを区別するは律法と考えられた」（ 波多野 1979 p.12）。よって「もと無意識的に行われた風俗習慣が神の命令として意識的に守られるべきものとなり、その煩瑣な規定を加うるに至っては、律法の宗教的意義は問題とならざるを得ない」（ 波多野 1979 p.15）。というのも国語さえ失ったユダヤ人にとって、国民の特色を最も表わすものは風俗習慣であるからである。「道徳は異邦人とてもっている。これに反して、風俗習慣において国民の特色は最も目立って現れている」（ 波多野 1979 p.15）。したがって民族に固有の風俗習慣を重んじるユダヤ教は、ユダヤ人にとっての宗教という党派性を脱し得なかった（ 波多野 1979 p.18~19）。そのようなユダヤ教にとって「神の国」の実現とはユダヤ人国家の再現という地上的なものであった。一方、イエスの「神の国」という言葉には、こういうユダヤ的地上的な部分もあったが、排他的ではなくより普遍的なものであった。「彼は政治より宗教を、ユダヤ人より人間を、イスラエルよりも神を重んじた」（ 波多野 1979 p.64）。このイエスの普遍的な側面を強調したのがパウロである。「十字架とメシアとの全く相容れぬ両端は今や相結び附いて、ユダヤ人的感情の誤れると律法の宗教上に全く価値なきとを証明した。それ故にユダヤ人たるはもはや宗教上必要の条件ではない。・・・かくしてパウロは、始めてキリスト教を、ユダヤ教を超越した人間的世界的なる新しい宗教として経験した」（ 波多野 1979 p.134）。また「イエスの神は愛の神であった」（ 波多野 1979 p.100）。然るにユダヤ教においては神は王であり、主であった（ 波多野 1979 p.17）。他方「パウロによれば、イエスは律法のなし得ぬ人類の救いをなし遂げんために世に送られたメシヤ（キリスト）即ち（特別の意味における）神の子である」（ 波多野 1979 p.168）。「パウロにとっては「恵みによってのみ義とせらる」と「信仰によってのみ義とせらる」とは全く同義であった。信仰とは何ぞ。そは人類を救わんとする神意に子供のごとく信頼するの謂である。吾人自身は罪人、何らほこるべき功もない、ただ謙遜と感謝とをもって神の恵みを受くべきのみである。この態度こそ信仰である」（ 波多野 1979 p.172）。このようにパウロは律法ではなく、信仰こそが救いに通じると解した。

^22^
そもそも背教するにしても有島武郎のようにそのこと自体を、大きな思想的宿命、自分の背負っていく枷として考えていた人も一定数いたと考えられるが、そのイメージばかりが従来先行していて、 佐藤 (2006) の指摘する老婦人のような人の存在にはあまり世間の意識が向かなかった可能性も考えられる。

^23^
プロテスタントだとプロテスタントのなかでの宗派自体は親と違うことは割とあるだけでなく、ロバート・ベラーにいわせると合衆国ではそれが子どもの自立、親離れの証として推奨されることもある(ベラー1985=1991 p.72)という。いずれにせよ多くの人はキリスト教の枠内には収まっている・あるいは近年では無宗教が増えているにせよ「キリスト教か無宗教(理神論等も含めて)」の枠内にはある。

^24^
このような津城のいう「自由キリスト教」の影響は、桜美林や玉川のみならず、同志社でもある程度想定されうる。竹中正夫 (1987) はアメリカン・ボードの背景にある思想は、理性主義的なユニテリアンではなく、人間の自由意志を批判的に論じたジョナタン・エドワーズにまで遡れるという（ 竹中 1987 p.131）。その上で日本組合基督教会の特徴として、自治の精神、自給の精神、自由の精神の三つを挙げる。これら三つはあとに挙げたものほど時系列的にも、後になる。新島襄自身が関与したのは一つ目のみである。三つ目の自由の精神についてはこの論文ではその端緒を 1898 年第3回日本組合基督教会での宮川経輝の講演に求める。よって新島の没年 1890 年より後になる。ここで宮川は 1. 精神を先にして形骸を後にする2.日本人の伝道は日本人が当たる 3. 寛容、の三つを唱えた（ 竹中 1987 p.136）。その上でこの論文では日本組合基督教会におけるこの自由の精神の代表格を、海老名弾正であるとする。海老名は三位一体説の解釈を巡って 1902 年福音同盟会から除名された。海老名の解釈は独自ではあったが決してユニテリアン的ではなかったと断った上で、竹中は海老名のキリスト像がキリストの神聖を強調する「高いキリスト論」ではなく、その人間性を強調する「低いキリスト論」であったとする。その上で竹中は「組合教会の中においても、キリスト論に巾があった」（ 竹中 1987 p.138）とし、組合教会は「高いキリスト論」も「低いキリスト論」も併存することを容認する寛容性があったと論じる。このような「低いキリスト論」の代表格であった海老名は同志社の第8代総長 (1920~1928) にもなった。また日本組合基督教会の三元老は上述の宮川、海老名と共に小崎弘道であるとするが、小崎に米国への伝道を勧める松山常次郎に小崎が「自分は同志社社長の時代に宣教師から異端と目されているから北米伝道はできない」と答えた言葉を紹介する（ 竹中 1987 p.143）。同志社「社長」は現在では「総長」に呼びかえられ、同志社の公式ウェブで第2代総長として小崎の名前が出ている。小崎は万国宗教大会で「日本のキリスト教の特徴について語ったが、宣教師との関係については、日本の教会においては宣教師は補助者であり、神学においても教会政治においても主導権は日本のキリスト者が握っている点を紹介した。特に神学について宣教師は正統主義神学を教えているが、日本のキリスト者の中では自由主義神学 (Liberal Theology) が主流である点を指摘した」（ 森 1990 p.16）。このようにユニテリアンではないものの、広義の「自由キリスト教」にその比較的初期の総長からして親和的であったということは言えようである。また戦後ではあるが 14 代総長は住谷悦治である。住谷は本稿筆頭筆者の研究対象中井正一の、戦前における人民戦線運動の庇護者のような存在であり、叔父の住谷天来から洗礼を受けたクリスチャンであり、マルクス主義者でもあった。

^25^
京大哲学科は東大哲学科時代にケーベルの影響を強く受けた人物が多かった。西田もケーベルの思い出を書いているし、本稿筆頭著者の研究対象中井正一の恩師深田康算は、漱石も寺田寅彦もケーベルの寓居を訪問するといつも深田がいたと記すほどの愛弟子であったし、波多野精一は、波多野の弟子の小原国芳の設立した玉川学園の公式ウェブによると、ケーベルが 「おれの弟子は波多野一人だ。外にはニヒツ（無い） 」と語っていたというほど優秀な弟子であった ( https://www.tamagawa.jp/introduction/history/detail_6750.html) （最終閲覧日 2022 年 12 月 4 日）。また和辻哲郎はケーベルに読んでもらうため英文で卒論を書き、ケーベルについての著書も弘文堂のアテネ文庫から出している。ケーベルの「キリスト教的信念は実に確固としていたのである。（波多野）博士は先生の個性におけるこのギリシア的自由とキリスト教的敬虔との総合に強く打たれた」（ 和辻 1962 p.27）。それに反して、東大哲学科を引っ張っていた井上哲次郎は内村の不敬事件を厳しく糾弾するなど、反キリスト教の立場を前面に出していた。反キリスト教的な井上よりもキリスト教的敬虔を示すケーベルに親愛の情を示したスタッフが京大哲学科には多かったといえるが、これは御大西田の意図による部分が大きいのか他の意思（例えば漱石と懇意な京都帝国大学文科大学初代学長狩野亨吉の意向）が強く働いているのかは、今後調べていきたい。なお西田のジェームズ開眼を元良の影響と、本稿本文では書いたが、万国宗教会議に参加した釈宗演の推薦によって米国に長期滞在していた大拙によるものとの説もある（ 橋本 2015 p.137）。なお元良も西田、大拙、漱石同様、円覚寺に参禅し釈宗演の指導を受けている。

^26^
例えば井上順孝は 井上ほか (1997) 所収の「大学生における宗教教育への関心と宗教意識」において宗教系中高卒業生（宗教系の中高の双方あるいはいずれかを卒業し、通っている大学は宗教系・非宗教系を問わない）と宗教系大学在学生卒業生を主に分析する。宗教系中高卒業生は「宗教に関係した学校に通った事で、その宗教を信じようと思ったことがありますか」という設問に対して「信じるようになった」 21.5% 、「興味をもったが信じない」 39% 、「信じない」 30.9% 、「反発を感じた」 4.5% であった（ 井上ほか 1997 p.149）。それらについて井上は次のように記す。「信じるようになった者が約二割、興味を抱いたのが約四割で、約六割に宗教系の学校に通ったことの効果が見られる。（中略）また、とくに信じようと思ったことがない者も約三割いるが、かえって反発を感じた者となると、五%弱である」（ 井上ほか 1997 p.149~150）。このように宗教系中学校高校の宗派教育の効果を一定程度認める記述をする。他方、宗教系大学生の分析に際しては同じ設問を比較していないのであるが、「今通っている大学に宗教的色彩がある」と感じている 66.9% に、どんな点に「宗教的色彩」を感じたかを自由回答で聞いた回答を紹介し、分析する。「肯定的、批判的、中立的な意見のうち、とりわけ目立って多いというものはなかった。ただ特徴的なことを一つ挙げれば、授業、行事の双方とも、強制された形態のものに対しては、強い反発があるということである。（中略）一般的に言って、中学や高校の宗教教育への反応に比べて、大学における宗教的色彩に対しては否定的反応が増える傾向にあると考えていいだろう」（ 井上ほか 1997 p.157）。井上はその理由を「強制への反感」にあると推察する。「やはり、かなりの学生は大学を「自由な学問の府」として捉えていると思われ、これと齟齬するような宗教的な授業や行事は、抵抗が大きいと推測される」（ 井上ほか 1997 p.157）。井上は「自由な学問の府」という部分を強調するが、ここの本稿本文のインタビューにあるように、小中高と上がるにつれて、宗教教育を担える人材が減っているという要因も、中高と大学の宗教教育の効果の差の原因にあるという可能性を我々は考えている。ただこれについても今後事例を積み重ねないといけないことは言うを俟たない。なお同じ著書の中で田口めぐみ「中等教育における宗教教育の意義」は上記の「宗教に関係した学校に通った事で、その宗教を信じようと思ったことがありますか」という設問についてもう少し細かい分析をしている。それによると「男子よりも女子の方が」そこの学校の「宗教に対し好感をもっている」（ 井上ほか 1997 p.215）とし、また「この結果の中で一番興味深いのは、宗教系の学校に中学校三年間または高校三年間しか通っていない学生と六年間通った学生との差である。ここでは、宗教教育が長期にわたってなされれば、その分影響も多いということが明確である。逆に反発を抱く学生の割合も多い」（ 井上ほか 1997 p.215）とも分析する。

## References

[ref41] 青野 太潮 : *パウロ―十字架の使徒.* 岩波書店;2016.

[ref42] 石黒 マリーローズ : *聖書でわかる英語表現.* 岩波書店;2004.

[ref1] 一般社団法人キリスト教学校教育同盟: 設置校数表: 2022年10月9日最終閲覧日. Reference Source

[ref2] 井上 順孝 : *宗教と教育―日本の宗教教育の歴史と現状.* 弘文堂: 國學院大學日本文化研究所編;1997.

[ref3] 井上 順孝 : *科研費報告書「現代日本における宗教教育の実証的研究」(1998~1999)報告書.* 國學院大學;2000.

[ref5] 井上 章一 : *ミッションスクールになぜ美人が多いのか.* 朝日新聞社;2018.

[ref6] 苧阪 良二 : 明治から昭和初期にいたる実験心理学の形成過程.心理学評論.1998;41(3):333–358.

[ref4] 内村 鑑三 :余は如何にして基督信徒となりし乎. 鈴木俊郎（翻訳）. 岩波書店.1958.

[ref43] 遠藤 興一 : 丸山眞男における宗教的実存のゆくえ(2). *明治学院大学キリスト教研究所紀要.* 2016;48:55–95.

[ref7] 学校法人雙葉学園: 教育理念.最終閲覧日2022年10月9日. Reference Source

[ref8] 加藤 周一 木下 順一 丸山 真男 : *日本文化の隠れた形.* 岩波書店;2004.

[ref9] クリーグ 波奈 : キリスト教主義学校の役割とその教育的意義. 東京大学教育学研究科紀要;2017;56:377–387.

[ref10] Goto Y : キリスト教主義学校に関する調査_ローデータ20200324.xlsx. figshare.[Dataset].2022a. 10.6084/m9.figshare.21366417.v1

[ref11] Goto Y : キリスト教主義学校調査票画面についての補足説明.txt. figshare.[Dataset].2022b. 10.6084/m9.figshare.21367632.v1

[ref12] Goto Y : キリスト教主義学校調査票画面についての補足説明.txt. figshare.[Dataset].2022c. 10.6084/m9.figshare.21367632.v1

[ref13] Goto Y : キリスト教主義学校 インタビュー調査 調査依頼書.docx. figshare.[Dataset].2022d. 10.6084/m9.figshare.21367776.v1

[ref14] 佐藤 八寿子 : *ミッション・スクール―あこがれの園.* 中央公論社;2006.

[ref15] 品田典子(講演者): Christian Today: 少子化、信徒教員の減少、迫る教育改革 カトリック学校の現状と将来(2018年11月29日23時05分).2022年10月9日最終閲覧日. Reference Source

[ref44] ジェイムズ W : *桝田啓三郎訳: 宗教的経験の諸相 上.* 岩波書店;1969.

[ref45] ジェイムズ W : *桝田啓三郎訳: 宗教的経験の諸相 下.* 岩波書店;1970.

[ref16] 島本 千也 : *鎌倉別荘物語.* 岩森書店;1994.

[ref17] 白百合学園中学校・高等学校: 宗教教育: 最終閲覧日2022年10月9日. Reference Source

[ref46] 田川 建三 : *イエスという男 第二版.* 作品社;2004.

[ref18] 武田 清子 : *背教者の系譜.* 岩波書店;1973.

[ref19] 武田 清子 : *未来をきり拓く大学―国際基督教大学五十年の理念と軌跡―.* 国際基督教大学出版局;2000.

[ref20] 竹中 正夫 : 日本組合基督教会の歴史と課題:その百年にあたって. *基督教研究.* 1987;48(2):128–172.3632547

[ref21] 田中 裕 : *「解説」西田幾多郎講演集.* 西田幾多郎著. 岩波書店、所収;2020.

[ref22] 玉川学園: 「波多野精一―小原國芳の師、玉川の丘へ」:2014.03.18.最終売閲覧日2022年12月4日. Reference Source

[ref23] 長野清泉女学院: よくある質問.最終閲覧日2022年10月9日. Reference Source

[ref47] 西田 幾多郎 : *善の研究.* 岩波書店;1979.

[ref24] 日本学術会議社会学委員会Web調査の課題に関する検討分科会: 提言 Web 調査の有効な学術的活用を目指して(2020).最終閲覧日2022年10月9日. Reference Source

[ref25] 橋本 満 : 近代日本における「宗教」の発見，あるいは宗教社会学の起点. *日本語・日本学研究.* 東京外大;2015;133–144.

[ref26] 波多野 精一 : *基督教の起源 他一篇.* 岩波書店;1979.

[ref27] 濱田 陽 : 「無宗教」への「対話」一チャペル・ウェディングと、日本のキリスト教一. 宗教と社会;2001;7:23–46.

[ref28] 廣瀬 つぎ子 : 雙葉小学校創立三十年記念誌 小百合(1941). のびのび会

[ref29] フェリス女学院中学校・高等学校: キリスト教活動: 最終閲覧日2022年10月9日. Reference Source

[ref30] ベラー, ロバート :1991. *心の習慣―アメリカ個人主義のゆくえ(原著1985=邦訳1991). 薗進ほか訳.* みすず書房;

[ref31] 堀 孝 : *「内村鑑三」と出会って.* 勁草書房;1996.

[ref32] マートン,ロバート・キング : *社会理論と社会構造(原著1949=邦訳1962). 森東吾ほか訳.* みすず書房;1962.

[ref33] 丸山 真男 : *日本の思想.* 岩波書店;1961.

[ref34] 森 孝彦 : シカゴ万国宗教会議:1893年. 同志社アメリカ研究 1990;26:1–21.

[ref48] Morimoto A : Two Types of Religion: In Search of a Better Foundation for Interreligious Dialogue in the Asian Context. *International Christian University, Humanities: Christianity and Culture.* 2002;33:19–26.

[ref49] 森本 あんり : *アジア神学講義-グローバル化するコンテクストの神学.* 創文社;2004.

[ref50] 森本 あんり : *アメリカ的理念の身体.* 創文社;2012.

[ref51] 森本 あんり : *反知性主義.* 新潮社;2015.

[ref52] 森本 あんり : *異端の時代―正統のかたちを求めて.* 岩波書店;2018.

[ref35] 森本 あんり : *不寛容論―アメリカが生んだ「共存」の哲学.* 新潮社;2020.

[ref36] 森本 あんり : 「日本人は多神教だから寛容」通説は本当なのか 統計調査をもとに考察する「無寛容の正体」(2021年2月8日). 東洋経済オンライン. 2022年10月9日最終閲覧日. Reference Source

[ref37] 立教女学院中学校・高等学校: 立教女学院のキリスト教教育: 最終閲覧日2022年10月9日. 立教女学院中学校・高等学校: 立教女学院のキリスト教教育: よくある質問.最終閲覧日2022年10月9日. Reference Source Reference Source

[ref38] 六十年史編集委員会編: 六〇年の歩み.立教女学院小学校.1997.

[ref39] 若松 英輔 : *内村鑑三.* 岩波書店;2018.

[ref40] 和辻 哲郎 : *和辻哲郎全集第6巻.* 岩波書店;1962.

