# Supplementary material for: Popular image of students enrolled in Christian schools and the founding purpose of Christian schools – A study based on a survey of youth living in Tokyo/Kanagawa Prefectures
Source: F1000Res. 2025 Feb 10;12:973. [Article in Japanese] Originally published 2023 Aug 14. [Version 2] doi: 10.12688/f1000research.127483.2 (PMC12509204; doi:10.12688/f1000research.127483.2)
Supplement: Peer review history

# Reviewer report: Reviewer response for version 2

**DOI:** 10.5256/f1000research.175855.r369842

**Published:** 2025-04-30

**Authors:** Yo Hamada

**Affiliations:** 1 Teikyo University, Tokyo, Japan

評者は、2025年2月10日に公開されたヴァージョン2のみを読み、以下の内容をまとめた。ヴァージョン1への二つの査読結果については、評者の査読プロセスでは参照していない。それは、ヴァージョン2が先の査読を受けて改稿されたものであり、また先行査読者と評者とでは研究アプローチや問題関心が異なるため、先入観なく多角的な議論を行う方が望ましいと判断したためである。

**本研究の意義**

 本研究は、風俗研究、宗教社会学、比較宗教学、キリスト教史、キリスト教思想、神学、宗教哲学、政治思想、哲学など、互いに参照される機会の少ない先行研究を横断的に参照・集積し、量的調査の質問項目と各項目の関係構造をデザインしている。

 そして、キリスト教主義学校通学経験者、その他の関与者、非関与者の三カテゴリーに分け、キリスト教主義学校の設立目的と生徒に対する一般的イメージを、ウェブモニター調査とクロス集計手法によって実証的に分析している。

 さらに、キリスト教式結婚式、クリスマス、お盆、初詣など、多様な質問を組み込み、日本人が神道・仏教と並んでキリスト教にも「宗教的な意義」を見出す傾向があることを示唆する仮説に、一定の根拠を与えることに成功している。（なお、宗教的な意義という表現は本論文筆者によるものではなく、論文中の「信仰」という用語の置き換えである。理由は後述する。）

 本研究の主たる意義は、以下三点にまとめられるだろう。

1. 多様な先行研究を参照・集積し、日本の宗教意識調査においてこれまでになかった独自の質問設計を行った点。
2. キリスト教徒が少数派である日本社会において、キリスト教主義学校が信者比率に比して一定の影響力を持ち得た要因を、東京・神奈川に限定しつつも具体的・実証的に示した点。
3. 現代日本人が、神道・仏教とともにキリスト教にもある種の宗教的意義を認める傾向があるという仮説に、限定的ながら支持を与えうるデータと解釈を提示した点。

**①** **多様な先行研究を踏まえた独自の質問設計**

 たとえば、井上章一ほか『ミッションスクールになぜ美人が多いのか』（2018年）と、井上順孝ほか『宗教と教育―日本の宗教教育の歴史と現状』（1997年）、同『「現代日本における宗教教育の実証的研究」（1998–1999）報告書』（2000年）は、アプローチも刊行年も大きく異なる。前者は風俗史を専門とする研究者がリードし、後者は宗教社会学的な視点に立つ研究であり、両者を一貫して参照するのは容易ではない。

 しかし、本研究はあえてこうした学際的参照を行うことで、これまでの日本の宗教意識調査にはなかった独自の問いの組み合わせを設計している。その上で、日本における宗教意識調査にとって、今後欠かせないツールとなりうるウェブモニター調査の新たな事例を提供している。

 なお、研究代表者も述べている通り、予算や期間に制約があるなか、質問設計に完璧を期することや、調査対象・範囲をすべて網羅することは不可能であった。そのため、本調査は東京・神奈川在住の15–29歳に限定されているが、これは本研究の欠点ではない。むしろ、日本において量的な宗教意識調査の蓄積が不足している現状（真鍋一史、2020年）を考えれば、この先駆的取り組みは意義深い。

 今後、本研究を出発点として、関西圏や他地域、あるいは30歳以上の層に対象を広げた調査・分析が行われれば、次の②③に関してさらに実証的知見が得られるだろう。

**②** **キリスト教主義学校の存在意義の実証的把握**

 研究代表者が述べるように、相対的にキリスト教主義学校が多い東京・神奈川に絞ったうえで、700件程度の回答を得ることで分析の解像度を確保するという選択は、調査予算の制約を考慮すれば現実的なものである。

 もちろん、東京・神奈川における傾向をそのまま全国に敷衍することはできないため、調査結果の解釈や論述においては、地域および年齢層の限定性を常に読者に意識させる配慮が求められる。

 その点を踏まえた上で、本調査は、キリスト教徒が少数派である日本社会において、キリスト教主義学校が相対的に一定の存在感と影響力を持ちうる背景を、具体的かつ実証的に示すことに成功しているといえる。

**③** **現代日本人におけるキリスト教意識の新たな可能性**

 本調査は、東京・神奈川在住の15–29歳を対象に、現代日本人が神道や仏教と並んでキリスト教にもある種の宗教的意義を見出していることを示すデータを得た。これは、日本人がキリスト教的な習俗（たとえば結婚式やクリスマス）は受け入れつつも、キリスト教そのものを排除しているという従来よく語られる見方を修正する必要があることを示唆している。

 また、本研究における質問設計と分析結果は、「信仰」「信者」「宗教」「習俗」「救済（救い）」「ご利益」といった基本概念について、それらの用語が一義的に理解されていない現状、そしてそのことが宗教意識の把握を難しくしている現実を浮かび上がらせている。

 たしかに、本研究における「信仰」などの用語は、厳密な定義のもとで一貫して用いられているわけではない。しかし、むしろその「ゆれ」自体が、現代日本人の宗教意識の多義性・多層性を映し出しているという点において、本研究は重要な研究事例を提供しているといえる。

**本論文の秀逸な分析手法**

 本論文の特筆すべき点は、調査対象を以下の三つに分類したうえで、さらに細かく分析解像度を高めたことである。

1. キリスト教主義学校通学経験者
2. その他の関与者
3. 非関与者

さらに、その他の関与者を細分化し、

- キリスト教主義学校を受験したことがある者
- 受験経験はないが受験を検討したことがある者
- 受験候補に加えたことはないが、オープンキャンパス等に参加意欲を示した者
- 自身は通学していないが、家族に通学経験者がいる者

と分類している。この精緻なカテゴリー設定により、キリスト教主義学校およびその生徒に対する各者のイメージの違いが立体的に把握できるようになっている。

 また、キリスト教式結婚式、クリスマス、お盆、初詣といった宗教文化行事に関する多様な設問を巧みに組み込み、日本社会における宗教意識の重層性を浮かび上がらせた点も秀逸である。

 これらの工夫により、本研究は単なる表面的なイメージ調査にとどまらず、現代日本人の宗教文化への関与様態を立体的に描き出している。以下、本論文が示した注目すべき調査結果を要約、抄録する。

 ［キリスト教主義学校の設立目的に関するイメージ］

- 「信者の獲得」を目的とするとの認識は、キリスト教主義学校への関与度が高い者ほど少ない傾向にある（有意差はないが傾向として認められる）。
- 「信者にならなくても、キリスト教の教えを守ることによって、救われる可能性のある人を増やすこと」が、通学経験者に多く選ばれた。
- 「信者の子弟の教育要求への対応」「教会以外の、キリスト教に触れる敷居の低い舞台としての役割を果たすこと」も、関与度の高い者により多く選択されている。

［キリスト教主義学校の生徒に対する印象（男女不問）］

- 「裕福である」という印象はある程度妥当とされるが、「容姿端麗である」との印象には相関がなかった。
- 「他人を思いやる心が豊かである」については、通学経験者よりも緩やかな当事者の方が選択率が高かった。
- 「外国語が得意である」「教養が豊かである」といったイメージは「該当なし/答えたくない」を除きこの順で多く選ばれ、後者についてはキリスト教主義学校への関与度が高いほど強く共有されている。（女性皇族に関する内容は、複雑になるため再録しないが、たいへん興味深い。）

［キリスト教式結婚式・クリスマスに関する意識］

- キリスト教主義学校の通学経験者ほど、キリスト教式結婚式に肯定的である。
- クリスマスに関しても、通学経験者は聖書を読む、祈る、聖歌を歌う、教会に行くといった行動を取る傾向が見られる（ただし、通学経験者や関与者になかでも行動を取らない者が9割以上を占める点には留意が必要）。

［他宗教行事への関与］

- キリスト教主義学校の通学経験者は、原家族とともに仏教・神道の行事（お盆、除夜の鐘、初詣）にも参加してきた割合が高い。

［キリスト教への関与意識］

- 「入信していなくても教えを守ることによって、救済やご利益（ごりやく） が得られる」と考える者の割合は、通学経験者で高く、次いで緩やかな当事者、非当事者の順で低下する。

**総括的意義と今後の展望**

 本論文は、東京・神奈川の15–29歳を対象とした2020年2月22日〜3月3日の調査に基づいている。このため、現代日本人全体について包括的な結論を導き出すものではないことには留意が必要である。しかし、以上の知見により、現代日本人、とくに都市部若年・青年層において、神道・仏教とともにキリスト教にも一定の宗教的意義を見出す傾向が確かに存在することが示唆される。

 これは、これまでの日本人宗教意識論に新たな視座を提供するものであり、今後のさらなる質的・量的研究に向けた道を拓く重要な貢献である。とりわけ、日本人の宗教的心性において、信仰・習俗・文化的受容がどのように重層的に交錯しているのかを検討するためには、本研究が設計したような多面的な質問設計とウェブモニター調査の手法が、今後いっそう有効なアプローチとなるだろう。

**本研究の特徴とその意義（長所と短所を含む）**

 本研究の意義は、上記①〜③にとどまらない。射程が現代日本人のキリスト教意識全般に及んでいるため、多様な先行研究や知見を積極的に視野に収めようとする姿勢が、論文全体に反映されている。

 この広い射程は、分野ごとに見ると必ずしも整理されていない部分もあり、専門研究の立場からは短所とみなされる可能性もある。しかし、他方で、それゆえに分野を越えた示唆を含み、学際的な研究を志向する立場からはむしろ大きな長所と評価できる。

 たとえば、評者自身の過去の研究である「『無宗教』への『対話』―チャペル・ウェディングと、日本のキリスト教―」（『宗教と社会』7、2001年）も参照されているが、同研究では主として宗教社会学的・社会史的アプローチに立ち、業界紙や各種メディア、当事者インタビューや内部文書の分析を行ったものであり、本研究とは方法を異にしている。

 当時のカトリック教会関係者による、カトリック系学校出身者が教会で結婚式を挙げたがるという趣旨の報告を含む内部文書もあったが、それは関係者の経験的な印象に基づくものであった。

 これに対し、本研究は、時代も方法も異なる複数の研究成果を相互に補完しあいながら量的調査の質問体系をデザインし、制約を抱えつつも人文学研究を生産的に前進させる道筋を一例として示しており、現代（示唆的には近代以降）の日本人のキリスト教意識という困難かつ挑戦的なテーマに対する重要な貢献をなしているといえる。

**評者自身の経験との接点・興味深い比較**

 なお、評者は『日文研叢書17 日本人はキリスト教をどのように受容したか 国際日本文化研究センター共同研究報告』（山折哲雄・長田俊樹編、1998年）にまとめられた共同研究の一部にオブザーバーとして参加した経験を持つ。

 本論文は重要な先行研究として『ミッションスクールになぜ美人が多いのか』（井上章一ほか著、2018年）を評価し、参照しているが、同共同研究の班員であった井上章一自身が、当時の共同研究に対する応答的、あるいは批判的な問題意識を示しているものと推測する。すなわち、本論文の射程は、日文研の同共同研究にも遡行しうるものと考える。

 また、評者は、真鍋一史（関西学院大学教授・当時）が主宰した宗教と価値意識に関する国際比較研究会に参加し、訪問留め置き法による社会調査「価値観と生活（宗教）意識に関する調査」（全国対象、1800名サンプリング、有効回答数882、2007年）の設計をめぐる議論に携わった。

 その際、「天皇」に関する質問項目を加える提案を行い、実際に採用された。

 本論文における女性皇族に関する設問への着目は、そうした経緯を持つ評者にとっても特に関心を引くものであった。

**本研究と従来の量的宗教意識調査との比較・補完性の評価**

 評者は、量的調査を専門とする立場にはないが、日本人の宗教意識研究において、「信仰」や「宗教」という概念の内実や定義をめぐる困難さ、ならびに、数理統計研究所の「日本人の国民性調査」やNHK放送文化研究所による宗教意識調査などにおいても、量的調査の意義と限界が併存している現状を見聞してきた。

 本研究は、これらの大規模調査には直接言及していないが、日本人の宗教意識を論じる際に、キリスト教について十分な知見を得ることが難しいという現実に対する応答とも読み取れる。

 特に、従来の調査では問われにくかった、入信を伴わないキリスト教文化受容の層を可視化しようとした点において、本研究は独自の貢献をなしていると評価できる。

 本研究が示した分析概念、方法、解釈の試みは、今後、日本人の宗教意識をテーマとする質的・量的研究の双方に対して、重要な示唆を与えるものと考える。

**問題点と改稿の提案**

 以上の意義と特徴を踏まえたうえで、評者が考える問題点と改稿に向けた提案を挙げておく。なお、ここでの提案は評者の立場と知見に基づくものであり、問題意識や専門領域によって重要度の感じ方は異なるであろう。

**・キリスト教主義学校の定員比率に関する記述**

 「1.2 本研究の目的」において、「日本のクリスチャンの数は日本の人口比で0.8%前後しかいないにもかかわらず、キリスト教主義学校が日本の私立学校のなかで一定程度以上の定員比率を誇り、また人気を集める学校も多いのはなぜか」と述べられている（PDF版5頁）。しかし、クリスチャン比率が明示されている一方で、キリスト教主義学校の定員比率については、具体的な概算や数値の提示がないため、不明瞭な印象を与えている。

 たしかに、16頁において、一般社団法人キリスト教学校教育同盟の資料に基づきプロテスタント系学校については言及されているが、カトリック系を含む東京・神奈川および日本全体のキリスト教主義学校に関する定員比率が整理されていない。

 クリスチャンの人口比とキリスト教主義学校の定員比率とのギャップを本論文の主題に位置づける以上、可能な限り概算でもよいので、現代日本（特に東京・神奈川）の私立・国公立学校全体に対するキリスト教主義学校生徒の割合を示しておくことが、論旨の明確化に資するだろう。

**・「トリプルな信仰」という用語の扱いについて**

 本論文は、郭南燕の論考「ミッション系大学の成功物語」を参照し、日本人が仏教・神道に加え、キリスト教にも親和的意識を持つことを「トリプルな信仰」と表現している（PDF版7頁）。しかしながら、郭の用いる「信仰」という語の意味が、本論文においてどのような精度で受けとめられているのか、明示的に定義されていないため、読者にとって判然としない印象を受ける。

 一般的に、「信仰」という語は、神仏など人知を超える存在への自覚的な信心や、宗教教団・宗派への帰属意識を伴うものと理解されることが多い。これに対して、郭の用法は、洗礼や入信を伴わず、文化・習俗的な受容を含めた広義の「宗教的心性」を指していると考えられる。したがって、本論文において「トリプルな信仰」という表現を用いるのであれば、「信仰」という語をあえて広義に用いることを、初期段階で作業仮説として明示しておく方がよい。

 たとえば、「本論文における「信仰』とは、洗礼・自覚的信心・教団所属を必須とはせず、人知を超える存在に対する肯定的な意識と、関連する学校・儀礼・行事等への参加・受容を含む広義の宗教的心性を指す」といった定義を示しておくことが望ましいのではないか。こうした整理により、一般通念上の「信仰」と本論文における特定の用法との差異が明確になり、読者に対しても誤解なく論旨を伝えることができるものと思われる。

**・宗教統計と宗教意識調査の違いについて**

 本論文では、神道・仏教・キリスト教・その他宗教に関する信者数の統計が扱われているが、宗派・教団側から提出される統計と、個人への宗教意識調査との間に存在するズレについて、やや整理が不足している印象を受ける。

 宗派・教団による報告データは、教勢を実際以上に大きく見せようとする傾向があり、一人が複数の宗教に関与しているケースも含まれるため、信者数の合計が総人口を大幅に上回ることが通例である。これに対し、個人ベースの宗教意識調査では、厳密な意味で自覚的に「信仰」を有する者の割合は、戦後以降の日本社会においては概ね3分の1程度にとどまるとされる。

 本研究はこの事実を踏まえていると推察できるが、読者に誤解を与えないためにも、序論など早い段階で、

- 宗派提出統計と個人調査との違い
- 本論文が依拠するデータの性格

について簡潔に整理しておくことを提案する。これにより、先行する各種統計・調査の取り扱いに対する本研究のスタンスがより明確になり、宗教意識に関する解釈の前提が読み手に伝わりやすくなるだろう。

**・ウェブモニター調査の詳細記述について**

 本研究では、サーベイリサーチセンターに委託して実施されたウェブモニター調査を用いているが、その実施条件や手続きについての記述が委託者側としての内容にとどまっており、調査方法の実際についての説明が十分でない印象を受ける。たとえば、

- 調査自体の呼びかけ文言と方法
- 調査協力者への謝礼の有無と額
- 回答者層のバイアス（たとえばキリスト教主義学校関係者への偏り）の可能性
- 調査実施にかかった費用の規模感

などについて、可能な範囲で記述を補足することが有益ではないか。

 特に、今回の調査においては、700名中108名（15.4％）がキリスト教主義学校通学経験者であった点が注目される。これは、東京・神奈川の実際のキリスト教主義学校定員比率と比べて高い可能性もあり、回答者層に何らかの自己選択バイアスが生じたのか否かを考察する手がかりにもなりうる。本論文において、調査設計や実施過程に関する透明性がより高まれば、読者は調査データの信頼性と限界をより適切に評価できるようになるだろう。

**・論文全体構成と論述の整理・簡素化について**

 本論文は、量的調査の結果報告に加え、3名に対する質的インタビュー、さらに宗教的寛容等に関する思想的考察（丸山眞男と森本あんりの議論の検討）を盛り込んでおり、これらが論文全体にわたって交錯している。その結果、読者には内容把握に一定の困難が生じ、また必要以上に表現上の繰り返しが見られることで、論文の分量も約10万字と非常に長大なものとなっている。

 量的調査の設計と結果分析を中心とする本論文の性格を踏まえれば、質的調査と思想的考察は、別稿として独立させ、本論文では最低限の関連箇所のみに言及する形式を取ることも可能であっただろう。もっとも、この重層的構成こそが本論文の個性であり、方法論的な意図に基づくものであると位置づけるなら、その旨を論文冒頭で明記しておくことが望ましい。

 また、「5 考察」（PDF版52–65頁）における森本の寛容論に関する展開などは、将来の発展的課題として位置づけ、より整理された形で提示することができれば、論旨全体が一層明瞭になると考えられる。

**・地域・年齢層限定性への留意と表記上の注意点**

 本調査は、あくまで東京・神奈川在住の15–29歳を対象としたものであり、現代日本人全体を代表するものではない。この限定性は、論文の各所で丁寧に言及されてはいるものの、さらに一貫して、本文全体のトーンとして強調する方が望ましいのではないか。とくに、結果の解釈や仮説の提示において、地域・年齢層の限定があることを読者が常に意識できるよう配慮されるべきである。

**・若干の表記修正案**

- 「後輩同僚日本文化センター（当時）の」（PDF版4頁）
  　→ 「後輩同僚である国際日本文化研究センター（当時）の」
- 「本稿を閉じることとしたいじることとしたい」（PDF版69頁）
  　→ 「本稿を閉じることとしたい」

また、井上順孝と井上章一という二人の「井上」が登場するため、本文中ではできるだけ区別して表記（たとえば「井上順孝ほか（1997）」と明記するなど）すると、読者にとって一層読みやすくなるだろう。

**・調査票・データへのアクセス案内の改善について**

 本論文では、「調査票はhttps://doi.org/10.6084/m9.figshare.21367632.v1（Goto, 2022c）にて閲覧でき、そこに調査のローデータも置いてある」（PDF版13頁）と案内されている。しかし、実際にアクセスしたところ、「キリスト教主義学校調査票画面についての補足説明.txt」というファイルは存在するものの、調査票本体およびローデータの具体的な取得方法については分からなかった。読者がスムーズに調査票やデータを確認できるよう、

- 調査票そのものへの直接リンク
- ローデータの具体的なダウンロード方法の案内

など、もう一段わかりやすい説明を加えると、論文の透明性がさらに高まるだろう。

 また、本文中に質問項目の一覧を順番通り簡潔にまとめて提示しておくと、調査設計の全体像（PDF版12頁の概念図）もより直感的に理解しやすくなる。

**総括**

 以上の通り、本研究は、

- 日本の宗教意識調査に新たな問いを開拓したこと
- キリスト教徒が少数派である日本社会において、東京・神奈川の15-29歳に限定した調査でありながら、キリスト教主義学校の存在意義を実証的に分析したこと
- 神道・仏教と並んで、現代日本人がキリスト教にも一定の宗教的意義を見出す可能性を示唆したこと

において、極めて重要な意義を有している。

 量的・質的・思想的アプローチを重層的に組み合わせるという本論文のスタイルは、一読して容易に理解し尽くせるものではないが、今後の人文学的宗教意識研究において、新たな地平を切り拓く契機となりうるだろう。

 本研究は、日本人の宗教意識、特にキリスト教との関わりをめぐる探究において、今後の議論の参照点となるべき成果であるといえるだろう。

**参考文献**

- 山折哲雄・長田俊樹編『日文研叢書17 日本人はキリスト教をどのように受容したか 国際日本文化研究センター共同研究報告』国際日本文化研究センター、1998年
- 濱田陽「『無宗教』への『対話』―チャペル・ウェディングと、日本のキリスト教―」『宗教と社会』7、「宗教と社会」学会、23-46頁、2001年
- 真鍋一史『価値観と生活（宗教）意識に関する調査』（納品CD-R）、中央調査社、2007年
- 濱田陽「インターレリジアス・エクスピアリアンスの学」『宗教多元主義を学ぶ人のために』間瀬啓允編、世界思想社、2008年
  （※真鍋一史「価値観と生活（宗教）意識に関する調査」および数理統計研究所「日本人の国民性調査」に基づき、日本人の宗教意識の三極構造を論じている。）
- 真鍋一史『宗教意識の国際比較―質問紙調査のデータ分析』北海道大学出版会、2020年

（該当する場合は要回答）統計分析および解釈は適切ですか。

はい

本研究は明確かつ正確に提示されたものであり、最新の文献を引用していますか。

はい

方法と分析について第三者による再現が可能となるよう十分な詳細が提示されていますか。

はい

結果の基礎となるソースデータはすべて入手可能で再現性を十全に保証していますか。

はい

結論は結果により妥当な裏付けを得ていますか。

一部該当

研究設計は適切で学術的価値がありますか。

はい

Reviewer Expertise:

比較宗教、日本文化、文明論

I confirm that I have read this submission and believe that I have an appropriate level of expertise to confirm that it is of an acceptable scientific standard, however I have significant reservations, as outlined above.

# Reviewer report: Reviewer response for version 2

**DOI:** 10.5256/f1000research.175855.r372741

**Published:** 2025-04-30

**Authors:** Mutsuko Tendo

**Affiliations:** 1 Miyagi Gakuin Women’s University, Miyagi, Japan

主要コメント

 　本稿はキリスト教主義学校の一般的イメージと、設立目的に焦点を当て、キリスト教主義学校への関与度から、通学経験者、それ以外の関与者、非関与者の3タイプで意識の違いを検討している。ウェブ調査（700名）に若干のインタビュー調査を加えた実証的調査の分析から興味深い知見が導きだされた。

 　先行研究において、キリスト教主義学校教育にかかわる諸研究の丹念な考察がなされ、「トリプルな信仰」、私立学校のミッションの目的、徳育としてのキリスト教などの論述、また社会学、哲学、思想史等の枠を超えた分析の視点を提示されたことは興味深い。

 　調査分析では、全体の作業仮説の提示は明確で設問は良く練られている。本稿ではとくに、キリスト教主義学校の設立目的、学校の生徒・学生イメージを取り上げている。調査項目で「信者獲得を目的としていない」との認識と、キリスト教主義学校への関与度（逆の相関）が示されるなど、データによる新たな知見が説得的に示された。　

 　後半のマートンの顕在機能、潜在機能の視点で、ミッションの再考、内面の感化、質的変化に言及している点も重要な課題を提起した。論文の随所に長年の研究蓄積の成果が結集した論述が光る。

 　課題としては、調査分析と、本稿の全体的論述が一層かみ合うことが望まれる。また応用可能性として、文化資本のジェンダー的特徴に踏み込んだ考察があってもよい。

 総じて、優れた研究論文であり、高く評価する。

 マイナーコメント

 　日本のクリスチャンの人口比の数字の齟齬。1.6％（要旨）, Cf.他所は0.8％。

 　論文としては長編の力作であるが、同様表現の繰り返しが多い。

（該当する場合は要回答）統計分析および解釈は適切ですか。

はい

本研究は明確かつ正確に提示されたものであり、最新の文献を引用していますか。

はい

方法と分析について第三者による再現が可能となるよう十分な詳細が提示されていますか。

はい

結果の基礎となるソースデータはすべて入手可能で再現性を十全に保証していますか。

はい

結論は結果により妥当な裏付けを得ていますか。

はい

研究設計は適切で学術的価値がありますか。

はい

Reviewer Expertise:

ジェンダー論、教育社会学

I confirm that I have read this submission and believe that I have an appropriate level of expertise to confirm that it is of an acceptable scientific standard.

# Reviewer report: Reviewer response for version 1

**DOI:** 10.5256/f1000research.139996.r207591

**Published:** 2024-07-04

**Authors:** Anri Morimoto

**Affiliations:** 1 Tokyo Woman's Christian University, Tokyo, Japan

本論文の要約

 　本論文の課題は、「日本のクリスチャンの数は日本の人口比で1.6%前後しかいないにもかかわらず、キリスト教主義学校が日本の私立学校の中で一定程度の定員比率を誇り、また人気を集める学校も多いのはなぜか」を究明することである。著者たちによると、この課題には二つの論点が含まれている。一つは前半部分「日本のクリスチャンの数が日本の人口比で1.6%前後しかいない」であり、もう一つは後半部分「キリスト教主義学校が日本の私立学校の中で一定程度の定員比率を誇り、また人気を集める学校も多い」である。前半と後半は「にもかかわらず」という相反の関係と捉えられており、本論文の課題はその関係に合理的な説明を与えることにある。

 　本論文の結論は、前半部分については「宗派に帰属しない日本人のトリプル信仰」からすると、「キリスト教に洗礼という手続きが不要であればキリスト教を含めトリプルな信仰をする人は多かったはず」であると推測し、1.6%という数字が「見かけ上の数値にすぎない」可能性を示唆する。

 　後半部分については、キリスト教主義学校のイメージが「教養が豊かである」「外国語が得意である」「他人を思いやる心が豊かである」「話し上手である」などの点において肯定的であること、また「布教という目的を日本のキリスト教主義学校はほとんど強く有していない」ことの二点を挙げ、それらが前半の事実と「矛盾しない」と結論づけている。

 評価

 　本論文の著者たちは、課題究明のためにコロナ禍の下でウェブモニター調査を行い、キリスト教主義学校に関する世間一般のイメージという把捉の難しい対象を統計的に数値化する方法を編み出している。特にその対象者を卒業生や在校生などの「強い当事者」、無関係の「部外者」、そして両者の間の中間的カテゴリーとして家族などの「緩やかな当事者」という三つのコホートに分けて設定し、それぞれにクロス集計をかけることで調査の精密度や信頼度を高めている点は、高く評価することができる。さらに、数値結果に具体的な肉付けをするためのインタビューを行い、ウェブや文献の資料も参看するなど、調査には独自で入念な手順が踏まれている。筆頭著者は思想・哲学系の社会学者であるが、対象領域は宗教学、風俗論、教育社会学、さらには日本思想史など複数の分野に関わっており、課題の設定や手法の選択に伴う困難と限界も十分に自覚されている。

 　調査の副産物として、本論文が一般社会にありがちな偏見の所在を示し、それらに根拠がないことを統計的に明らかにしている点も評価に値する。キリスト教主義学校に通う女子が「きれい」「容姿端麗」であるというイメージは、今日の倫理感覚からすれば調査項目とすること自体がはばかられるが、それをあえて項目化したことで明示的に否定する結果を導くことができている。キリスト教主義学校が「教団組織の資金集め」を目的としている、という印象も、著者たちが「現実にはありえない」選択肢と認識しつつ項目化したことで、それが無根據であることが示される結果となっている。

 　他方、改善が可能な点もある。まず、質問の項目や対象者などの偏差に注意が必要である。実際の質問項目を見ると、プロテスタントとカトリック、女子校・男子校・共学校の違いについては著者たちも留意しつつ調査を進めていることが認められる。しかし、仏教系や神道系の学校は質問項目には含まれているものの、詳細な比較検討がなされていない。たとえば「他者を思いやる心」の涵養などは、仏教系学校でも十分に高い数値となることが予想されるし、他宗教の行事に参加することへの寛容度の比較も分析可能なはずである。日本のキリスト教徒を人口比で1.6%（ないし1.0%）とすることの出典や根拠、東京都と神奈川県の数字を「日本」の数字へと外挿することの是非などにも説明が望ましい。

 　これらの空隙を補填するかのようにいくつかのインタビュー会話が記載されているが、いずれも個的で多義的なエピソードで、客観的なデータを補完するとまでは言えない。文献資料の参看となると、唐突に現代でなく明治時代の内村鑑三や海老名弾正や波多野精一が引用され、あるいはトマス・アクィナスの修道会入会理由が引用されており、本来の文脈を顧慮しない恣意性が散見される。

 　本論文の課題設定そのものの評価については、改善提案に記しておく。

 先行研究

 　前半の課題は、これまで「日本のキリスト教受容」「キリスト教の土着化・文脈化」という問題群で扱われてきたやや古典的な問いである。先行文献も豊富だが、本論文ではわずかに武田清子と丸山眞男が引用されているのみである。本論文で「トリプル信仰」として引用されている信仰の多重性や複相性についても、国内外で議論が重ねられてきた。多くのアジア的な宗教にとり、宗教は意識的な決断の事柄であるよりも儀礼や習慣などの生活様式に密着した事柄である。入信儀礼の位置づけも多様であり得るため、ある宗教の理解を他の宗教の理解にあてはめることには慎重でなければならない。「トリプル信仰」は仏教や神道の視点から論じられたものであるため、キリスト教の視点から論じられた同種の議論を参看することは有益と思われる。森本あんり（2004年）は、特にキリスト教のアジア的な文脈化を類型論とともに論じており、宗教信仰の重層化とりわけ二重信仰のあり方に光を当てている。同書には、ビーヴァンスやシュライターなどこの問題群を扱うのに不可欠と思われる先行研究も紹介されている。他に、南山宗教文化研究所にも豊富な文献の蓄積がある。

 　他方、キリスト教に洗礼が必要か否か、救済に洗礼が不可欠か否かという問題群も、キリスト教神学の歴史において繰り返し問われ続けてきた。著者たちが論文末尾で引用しているロジャー・ウィリアムズはその論者の一人だが、古代中世を通して交わされてきた議論を概観するには、森本あんり（2018年）と同書に付された文献一覧が便利である。

 　後半の問いは、本研究にも引用されている「キリスト教学校教育同盟」に照会すれば、過去の事例研究やデータ資料が得られるはずである。

 改善提案

 　本研究課題前半部の問いについては、「キリスト教に洗礼という手続きが不要であればキリスト教を含めトリプルな信仰をする人は多かったはず」という仮定に基づいた推測をもって結論とされているが、その推測を裏付けるためにはやや異なった質問項目が必要かもしれない。著者たちも一部自覚しているとおり、「関心がある」と「入信する」とは違うし、「内心で信じている」のと「それを公言する」のも別である。実際の質問項目でも「文化資源としての側面」と「純粋な宗教としての側面」という二面が区別されているが、これらの区別は連続的なので数値化するには工夫が求められる。

 　上記推測が正しいかどうかを確認するには、「もし洗礼という手続きが不要だったなら、あなたは自分のことをキリスト教徒だと思いますか」といった趣旨の設問がもっとも直接的だろう。だが、その前提をなす仮定「もし洗礼が不要だったら」は、「キリスト教徒であること」の内容にあらかじめ大きな変更を加えており、設問自体を無効化しかねない。それは「もし野球にバットが不要だったなら、あなたは野球選手になりますか」と尋ねることに似る。

 　宗教にはそれぞれ固有の準拠枠があるので、これを大きく改変することを前提とした上での設問には限界が伴う。日本の仏教や神道では明確な儀式を経ずにも信徒であることが可能であることから、他宗教でもそのような儀式は省略可能だ、と想定されているとすれば、そこには調査者の宗教観が無自覚に前提され作用していることになろう。

 　文中には内村の「無教会主義」も触れられている。無教会では洗礼という儀式が不要なので、著者たちの想定に従うならば、ここには多くの日本人キリスト教徒が集うはずだが、現実には内村の時代から今日に至るまで、無教会の信徒は極端な少数派に留まっている。武田清子の引用によれば、無教会主義はすでに日本で正統の一部と見なされているので、極端な少数派であることの理由を無教会主義への異端視に求めることはできない。とすると、本研究前半部の結論的推測には別の理由づけが必要となるように見える。

 　後半部の結論についてはおおむね分析の結果を信頼できるが、日本のキリスト教主義学校に多くの生徒・学生が集まることは、それらの学校の一般的なイメージが肯定的であることや学校が「布教」を主目的としていないことと「矛盾しない」、という判定だけではやや漠然としている。これも著者たちが自覚的に記していることであるが、キリスト教主義学校を志望する動機には、「進学・就職実績」など教育理念とは無関係の世俗的な利点が作用することが知られている。前半と後半が「矛盾しない」という判定を越えて、より積極的な因果関係ないし相関関係を見出すことに焦点が当てられるならば、本研究はいっそう大きな意義をもつことと思われる。

 参考文献

 森本あんり『アジア神学講義』、創文社、2004年。

 森本あんり『異端の時代』、岩波書店、2018年。

 Bevans, Stephen B. *Models of Contextual Theology*, Revised and Expanded Edition. Maryknoll, New York: Orbis Books, 2002.

 Hesselgrave, David J. and Edward Rommen. *Contextualization: Meaning, Methods, and Models*. Grand Rapids, Michigan: Baker Book House, 1989.

 Morimoto, Anri. "Two Types of Religion," *Humanities: Christianity and Culture* 33 (2002): 19-26.

 Schreiter, Robert J. *Constructing Local Theologies*. Maryknoll, New York: Orbis Books, 1985.

 van der Veer, Peter. "Syncretism, Multiculturalism and the Discourse of Tolerance." Charles Stewart and Rosalind Shaw, eds., *Syncretism/Anti-Syncretism: The Politics of Religious Synthesis*, 196-122. London and New York: Routledge, 1994.

 Yung, Hwa. *Mangoes or Bananas? The Quest for an Authentic Asian Christian Theology*. Oxford: Regnum Books International, 1997.

（該当する場合は要回答）統計分析および解釈は適切ですか。

一部該当

本研究は明確かつ正確に提示されたものであり、最新の文献を引用していますか。

一部該当

方法と分析について第三者による再現が可能となるよう十分な詳細が提示されていますか。

はい

結果の基礎となるソースデータはすべて入手可能で再現性を十全に保証していますか。

はい

結論は結果により妥当な裏付けを得ていますか。

一部該当

研究設計は適切で学術的価値がありますか。

一部該当

Reviewer Expertise:

神学・宗教学

I confirm that I have read this submission and believe that I have an appropriate level of expertise to confirm that it is of an acceptable scientific standard, however I have significant reservations, as outlined above.

# Response

**Published:** 2024-12-18

**Authors:** Yoshihiro Goto

**Affiliations:** University of Tsukuba, Japan

森本先生への回答

 学長としての業務多忙な中、真摯な査読をしてくださり、誠に感謝に堪えません。

 f1000から査読者候補に挙げるように依頼された際、ご多忙な先生を無礼にも候補の一人に挙げさせていただき、一度は辞退されたにもかかわらず、最終的には、幸運にも査読いただけたことは光栄の限りです。

 失礼を顧みずに先生を査読者候補に挙げさせて頂いた主な理由は、先生の『異端の時代』と『不寛容論』を読ませていただき、非常に感銘を受け、後者については本稿の第一稿でも、引用させていただいていたからです。

 特に『異端の時代』と『不寛容論』はわれわれの研究グループが、本稿のもととなった科研費の助成期間が終わり、その次のグラントを応募する際の研究テーマである、日本人の寛容性の問題に直結するだけに、本稿の改訂稿では「今後の課題」の部分でも取り上げさせていただいております。

 そもそも本稿は、寛容の問題をメインの主題として扱っております。特に丸山真男の、≪キリスト教とマルクス主義が首尾一貫性をつらぬくがゆえに、無限抱擁的な日本人から排除させる≫というテーゼについて、令和における若年層にも妥当するか否かの吟味・検証が、主たる課題です。よって上の段落で申し上げた、次なるグラントのテーマとして記したテーマに直結します。その意味でも先生の査読は非常に有難い限りです。

 このような丸山のいうテーゼの検証が課題ですので、本稿の本来の目的は、「日本のキリスト教会やキリスト教学校当局がどう考えているか」ということではありません。「日本のキリスト教会やキリスト教学校当局の考え」そのものではなく、「それらを、日本人がどのように捉えているかを知ること」が、本来の直接の目的です。もちろん意図してこのような限界を設けたと同時に、時間的金銭的コストの限界および調査がコロナ禍のもとでおこなわれたという物理的限界の部分も少なからずございます。しかし、「日本のキリスト教会やキリスト教学校当局の考え」を日本人がどう捉えているか知ること」それ自体が本稿執筆の主たる目的ですし、それがウェーバーのいう知的禁欲にも適うと考えています。

 言い換えると「神仏習合」という日本人の宗教観から由来する、「トリプルな信仰」等の日本人のキリスト教イメージと、丸山のいうような首尾一貫性を重んじるという日本人のキリスト教イメージのどちらかが正しいのかという、イメージの検証を主目的としております。イメージの方であって実態の方ではありません。ただ、筆が走って、イメージだけではなく、実態に及ぶような表現をしている部分も多々あったので、それらはイメージに議論を限定するように改稿いたしました。というのも、日本のキリスト教の実態自体にも興味はあるし、それに至る結果を出したい野心もあり、それゆえやや筆が走った箇所があるものの、我々のグループのバックグラウンドからして、そこに及ぶ能力も素養も到底ないと判断されるからです。なお、「変更履歴稿」の方で二重取り消し線のある部分が、改稿に際して削除させていただいた部分になります。

 よって今回の改稿の方針を巡る共著者の会合で、この主たるテーマの考察が一段落したところで(5.3)、論文を実質〆て、あと「5.4 布教・ミッションの目的と、顕在機能、潜在機能」と「5.5 以上を踏まえた、キリスト教主義学校関係者のミッション、他者救済の形の推測」は「結論と今後の課題」にて、わずかに触れるという案が強く出されました。この方がより禁欲的で、直接の研究目的を越えたことを言わずに済み、いわゆる「スリムな合格答案」に近づくからです。ただ、先生の査読で挙げていただいた、先生の関連著作に言及することは、寛容の問題に関する次のグラントに通じることでもあるので、論文を途中で〆てしまう案はとりあえず保留といたしました。

 なお丸山真男のテーゼの検証を論文の柱のテーマにしていていますので、丸山の恩師は南原繫で、南原の宗教上の恩師は、内村と新渡戸となります。改訂稿の12ぺージ（注：個別にお送りする変更履歴入りのファイル上のページ数です。以下同様）に新たに引用させていただいた以下の遠藤興一論文とその我々の説明が一つの論拠です。以下の「」部分は、改訂稿の原稿でその中の『』部分は、新たに引用した遠藤の論文からの引用部分です（個別にお送りする変更履歴入りのファイル上では以下の「」は括弧なし、『』が「」となります。）。

 「もっとも遠藤 (2016)）では、丸山のキリスト教からの影響を次のように記す。『南原、三谷は内村鑑三から薫陶を受けた無教会キリスト者であり、丸山は両者から高校時代には法律哲学（法制）を、大学に入ってからは政治哲学を学ぶ経験は持ったものの、その後、キリスト教を介して、同信となることはなかった。しかし、丸山にとってこのドストエフスキーは、一方で自らの社会主義観とも深くつながっており、その意味で重要な作家であったが、他方キリスト教の理解という点でも、深く実存的な影響を受ける作家となった』。したがって、内村、南原を通じてキリスト理解の下地を得て、さらにドストエフスキーを通じて丸山のキリスト教観は形成されているといえる。」。

 よって、二人に頻繁に言及することは、申し訳ございませんが、文脈を無視した恣意的引用ではなく、研究目的に添った引用と考えさせていただきたいと思います。よって先生の査読ペーパーの「文献資料の参看となると、唐突に現代でなく明治時代の内村鑑三や海老名弾正や波多野精一が引用され、あるいはトマス・アクィナスの修道会入会理由が引用されており、本来の文脈を顧慮しない恣意性が散見される」というご指摘のうち、内村以外は対応させて頂きますが、内村に限っては、引用を原則的に控えない方針で、今回の改稿の段階では、進めさせていただいています。また波多野に関しては、旧稿での注での説明は「唐突に現代でなく明治時代の」とあるので、一部削除させていただきますが、本文（改訂稿の75ページ）はパウロについての記述で、「唐突に現代ではなく」には該当しないと考えて、残しております。またパウロについてこの段落及びそれ以下5.2の終わりにかけて追加もさせていただきました。

 つぎに「しかし、仏教系や神道系の学校は質問項目には含まれているものの、詳細な比較検討がなされていない。たとえば「他者を思いやる心」の涵養などは、仏教系学校でも十分に高い数値となることが予想されるし、他宗教の行事に参加することへの寛容度の比較も分析可能なはずである」というご指摘についてですが、「仏教系や神道系の学校は質問項目には含まれている」ものの、キリスト主義学校のように、学校の設立目的や学生のイメージの設問は付けませんでした。これは質問のスペース上の問題以上に、類似の質問は、ほぼ似た回答の分布になる傾向があるということがあります。またそれ以上に元日本宗教学会長の井上順孝グループの一連の研究である程度結果が出ているということもあります、たとえば改訂稿の62～63頁を、4パラグラフと少し長くなりますが、次の段落以下で≪≫で引かせて頂きます。まず最初の二段落は我々の調査結果の分析で、三段落目の「なお」から井上順孝グループの本からの補強となっております。なおこれは査読前の原稿にもあったものです。

 ≪これは（ 表3-3-8）カイ二乗<0.01 で有意差がある。キリスト教主義学校に通ったことのある者ほどお盆の迎え火を「今でも原家族と行っている」人が多く、キリスト教主義学校と関わりがない者は「原家族と行った記憶はない」者が多い。これも（ 表3-3-9）カイ二乗<0.01 で有意差がある。実数は多くはないが、キリスト教主義学校の当事者性が高い者ほど、原家族と除夜の鐘の鐘付きに今でも行っている。

 この初詣についても（ 表3-3-10）カイ二乗<0.01 で有意差がある。「キリスト教主義学校に通ったことのある」者と「キリスト教主義学校に通ったことはないが、関わったことがある・関りのある人がいる」者の間には差があまりないが、それら何らかの意味でのキリスト教主義学校当事者と、キリスト教主義学校への関わりのない者との間では、差がある。

 なお、『「現代日本における宗教教育の実証的研究」(1998~1999) 報告書』では、「あなたは今年の初詣はどうしましたか」という問いに「家族と行った」「家族とは別に行った」「行った家族もいるが自分は行かなかった」「家族の誰も行かなかった」「その他」の選択肢を用意してある。宗教系大学生のみについてであるが、大学の宗教別のクロス集計をしている（ 井上ほか 2000 p.44）。宗教系大学の合計だと「家族と行った」24.5%、「家族と別に行った」22.4%、「行った家族もいるが自分は行かなかった」17.6%、「家族の誰も行かなかった」23.1% である。カトリック系大学生は「家族と行った」38.3%、「家族とは別に行った」21.7%、「行った家族もいるが自分は行かなかった」13.5%、「家族の誰も行かなかった」21.7% で、宗教系大学の合計の選択肢の 1，2 の合計が 46.9% であるのに対して、カトリック系大学では 60.0% である。プロテスタント系大学ではそれほど宗教系大学の合計と差はなかった。因みに神道系大学では「家族と行った」23.0%、「家族とは別に行った」25.4%、「行った家族もいるが自分は行かなかった」23.3%、「家族の誰も行かなかった」22.3%である。選択肢 1，2 の合計が 48.4% で宗教系大学の合計より少し多いだけで、カトリック系は神道よりも初詣に積極的という結果が出ている。同書の分析では「カトリック系が約 6 割、天理大と仏教系他学科（注記；この調査報告書では仏教系大学について一般学科と仏教学科を分けた集計を行っている）と神道系とプロテスタント系が約 5 割であるのに対して、仏教学科 2 割 5 分、創価大 1 割強とあとの 2 者が比較的割合が少ないことが理解できる」（ 井上ほか 2000 p.44）と記される。 当然、これはキリスト教主義学校のみに焦点を当てた研究ではないので、この分析は妥当ではあるが、カトリック系が神道系を超えているというある意味非常に意外な結果については特にこだわっている印象はない。しかし我々の視点からこの 20 年前の研究を見返すと、「トリプルな信仰」に通じる寛容性の涵養をカトリック系の大学はこの時期既に行っていた可能性があるとも評せて、我々の調査結果に通じる結果がすでに出ていたともいえる。

 以上の3つの我々の調査による表から、キリスト教主義学校当事者の方が（通学経験者と緩やかな当事者との差はケースバイケースではあるが）、幼少期に原家族とお盆や除夜の鐘や初詣など、キリスト教「以外の」宗教行事に積極的に触れてきていることが分かる。つまり、この結果は排他的なキリスト教像とは逆とも評せるし、またキリスト教主義学校への期待も、キリスト教という特定宗教への期待というよりは宗教教育による情操の涵養への期待があることが想定できる≫。

 この上の引用部分の下から2パラグラフ目のアンダーライン部分、「当然、これはキリスト教主義学校のみに焦点を当てた研究ではないので、この分析は妥当ではあるが、カトリック系が神道系を超えているというある意味非常に意外な結果については特にこだわっている印象はない。しかし我々の視点からこの 20 年前の研究を見返すと、『トリプルな信仰』に通じる寛容性の涵養をカトリック系の大学はこの時期既に行っていた可能性があるとも評せて、我々の調査結果に通じる結果がすでに出ていたともいえる」という我々の井上グループへの評言部分から考えて、森本先生の仰る、「しかし、仏教系や神道系の学校は質問項目には含まれているものの、詳細な比較検討がなされていない。たとえば「他者を思いやる心」の涵養などは、仏教系学校でも十分に高い数値となることが予想されるし、他宗教の行事に参加することへの寛容度の比較も分析可能なはずである」というご指摘のうちの、後半部分「他宗教の行事に参加することへの寛容度の比較も分析可能なはずである」については先行研究で解決済みといえると考えました。もちろん、調査票並びに論文の紙幅があれば、20年後の変化にも論及すべきではあることは当然でありますが。

 また「日本のキリスト教徒を人口比で1.6%（ないし1.0%）とすることの出典や根拠、東京都と神奈川県の数字を「日本」の数字へと外挿することの是非などにも説明が望ましい。」というご指摘ですが、前半のご指摘の出典は井上章一の本でしたが、その本の中でのソースの出典はなかったので、公的なデータとして令和5年度版文化庁『宗教年鑑』を使うことにしました。それは0.8%でした。よって「日本のクリスチャンの数は日本の人口比で0.8%しかいない」と改訂稿の5頁に記し、それ以降のページでは、「1.6％」を「0.8％」に置換させていただきました。

 前段のご指摘の後半の「東京都と神奈川県の数字を「日本」の数字へと外挿することの是非」に関しては査読前の原稿にもあったものですが、改訂稿の20ページの以下の≪≫部分の記述が答えになると考えております。

 ≪東京都と神奈川県に居住地域を絞ったのは、これらの地域にキリスト教主義学校が多いからである。特に神奈川県はフェリス女学院等明治時代初期からキリスト教主義学校があっただけでなく、戦後GHQの政策で、旧海軍用地や農地改革等で接収した土地を横須賀の進駐軍との絡みで、キリスト教主義学校の校地のために優先的に払い下げたとの説もある（島本 1994）。以下の数字にカトリックは含まれないし、プロテスタントでも漏れはあるものの、一般社団法人キリスト教学校教育同盟の一覧では東京都23学校法人、神奈川県11、愛知県4、大阪府7、兵庫県8、福岡県4で、他の都道府県は3学校法人以下である（最終確認日2022年10月17日）。

 キリスト教主義学校が身近にない調査対象者だとイメージを浮かべにくい点が、これらの地域の住民に限定した理由である。さらにそのことと関連するが、それ以上に、本調査全体の枠組みにもこの限定は関わっている。再三申し上げているように本調査ではキリスト教主義学校に通ったことがある・あるいはいま通っている当事者をいわばキリスト教主義学校当事者として、それらの者の保証人等家族や志望したことがある者、兄弟姉妹に通学者のいる者、オープンキャンパスに行ったことのある者などを、当事者の周辺の者・緩やかな当事者とし、これらに該当しない者を部外者の者として、これら三者の間での意識の差の有無をみようとしている。これらの差を本調査の他の質問とのクロス集計の分析の軸として想定した。よって当事者や当事者周辺の者（緩やかな当事者）を一定数以上確保するためには、キリスト教主義学校が通学圏にほとんどない地域の調査対象者を減らすことが、有効と考えた≫。

 当然「当事者や当事者周辺の者（緩やかな当事者）を一定数以上確保」できない場合、サンプル数をもっと増やさないと、「当事者や当事者周辺の者（緩やかな当事者）」と「当事者以外の者」との違いに、統計的有意差が出なくなってしまいます。

 なお、「「トリプル信仰」は仏教や神道の視点から論じられたものであるため、キリスト教の視点から論じられた同種の議論を参看することは有益と思われる。森本あんり（2004年）は、特にキリスト教のアジア的な文脈化を類型論とともに論じており、宗教信仰の重層化とりわけ二重信仰のあり方に光を当てている。」ということでしたので、再提出稿でそれについて引用させていただきました。少し長くなりますが、以下≪≫で囲った部分を加筆しています（再提出稿84頁以降）。（なお、これより5パラ前からの記述も先生のご著書からの説明です）。

 ≪例えば森本の『アジア神学講義』（p.211）では、「二重信仰」の問題が取り上げられる。「従来のキリスト教的な理解では、信仰は唯一的であって、一つの宗教をもつということは他の一切を棄てるということである」。しかし「近年第二版が発行された『世界キリスト教年鑑』には、「キリストを信ずるが洗礼を受けていない信徒」ないし「キリストを信ずる非キリスト教徒」というカテゴリーが設けられている」。この年鑑の編者によると「「ヒンドゥー教、イスラム教、仏教などの信徒が、自分の第一の信仰としてイエス・キリストを信じていながら、キリスト教会には属さずに自分の文化的な伝統に留まることを選び、その宗教の内部でキリストを証しつづける」場合である」という。

 さらに森本はアジアで深刻なのは、「「宗教」概念の根本的な理解の差に由来する二重性である」として、次のように述べる。「多くのアジア人にとり、宗教は意識的な決断の事柄である以前に、儀礼や習慣などの生活様式に密着した事柄である。これらの「道」を「宗教」 として捉えようとする前提そのものが、実はかなり一面的なものである公算が大きい。つまり、アジア的な「二重信仰」の場合には、キリスト教と他宗教が同一人格において 「並在」するといっても、それはキリスト教的な宗教理解から見た場合であって、他「宗教」の側からすれば、リンゴとミカンのようなカテゴリー内部の衝突ではなく、リンゴとハチミツのように別種の組み合わせとして矛盾なく受け止められていることがある。そもそも人がある宗教を「選ぶ」という理解は、 複数の宗派や教派が手近に混在し、あたかも市場で買い物をするようにその中から自分に適したものを選び取るという市場経済型自由教会型の社会構造を前提している。アジアの大多数の人々は、ある「宗教」の中へと生まれ落ちるのであって、選択や決断を基盤としたこのような「宗教」観を共有してはいない」と述べる。要は、宗教Aと宗教Bは論理学的に排他的であるという従来の発想は、アジアでは必ずしも自明ではないということになる。もっとも、ヨーロッパ自身の自己解釈、あるいは丸山が整理したヨーロッパの特徴とされる「首尾一貫したドグマとしてのキリスト教」でさえ、キリスト教受容後のヨーロッパでも、ギリシャやローマ、あるいはゲルマンや北欧の神話が変わらずに親しまれてきたことを勘案すれば、実際のところは二重信仰性の実態があり続けてきたものと解釈する可能性さえ充分にある。

 以上の議論を我々の文脈に即していえば、「選択や決断を基盤としたこのような「宗教」観を共有してはいない」ということは、「二重信仰」さらに「トリプルな信仰」も否定しきれないということになる。さらに『アジア神学講義』の211ページでは「仮にもある文化に福音が伝えられ受容され意義を保ち続けているならば、そこには必ず何らかの変容が認められるはずである」と述べる。もちろん「まったく宿主文化に埋没してその存在すら気づかれないようでは、そもそも文脈化の意義が失われてしまう」が、「「二重信仰」の文化を背景として考えるならば、それも優れて文脈化の一形態であるかもしれない」といい、山本七平の「日本教キリスト派」に言及し、それを「人間論型」の文脈化理解からすると、むしろ賞賛されるべき文脈化の成功例ということになるであろう」と評する。『アジア神学講義』のこの「二重信仰」から「日本教」のくだりのみを取り上げれば、この本も「トリプルな信仰」を否定していないとも読みうる。このことは同書の前の方で武田清子や森岡清美らの社会学者が、原型なり祖型がどこかにあるという前提があるという点を批判することにも通じる。『アジア神学講義』で紹介されたアジアの神学者たちの議論をニーチェによるパウロ以降のキリスト教批判と同じ論理構造を持つことを指摘する森本も、同様に、土着化される以前の「純粋なキリスト教」というものはどこにも存在しないと語る（p.7）。イエス当人も当時のご当地的なユダヤ教理解をしていたものであり（田川建三『イエスという男　第二版』作品社、2004）、宗教がどれほど高度に論理的に鍛えられて普遍化を行ってても、土着的な要素が皆無であることは不可能である。

 しかし、ただ神学は、文脈化を越える努力を求められる。「神学はそれゆえ、自己の文脈を越えたより広い文脈に無関心であることはできない」（p.9）。「本来的な意味でのテキストは、時にコンテキストを超越する「権威」をもち、おのずと受け手のコンテキストをある「審判」のもとに置くはずである」（p.10）。そしてこの森本の書の全体は文脈化をこえる志向を持ち続けつつ、文脈しようとしたアジアの神学者へも敬意も示すという矛盾した姿を、あえて示しているように思われる。むしろ、普遍化と文脈化という真逆のベクトルを両方とも必要な相補的なものと見なしたうえで、両者のサイクルを上手く回すことを主張していると解釈する方が整合性がある。森本は『アジア神学講義』で触れたアジア人の神学者たちの問題意識や意義は否定していないが、普遍化の不足と思える個所には随所で批判を加えており、一方でその目的は彼らアジア人神学者たちの問題意識がこのままでは埋もれてしまうという危機感に根差しているからである≫。

 なお、先生から頂いた査読ペーパーの「改善提案」の項の前半３パラグラフは、調査が終わったあとゆえ、残念ながら本稿では改善しようがありません。今後の調査研究の際に検討したいと思います。

 それに続けて査読ペーパーにて「文中には内村の「無教会主義」も触れられています。「無教会では洗礼という儀式が不要なので、著者たちの想定に従うならば、ここには多くの日本人キリスト教徒が集うはずだが、現実には内村の時代から今日に至るまで、無教会の信徒は極端な少数派に留まっている。武田清子の引用によれば、無教会主義はすでに日本で正統の一部と見なされているので、極端な少数派であることの理由を無教会主義への異端視に求めることはできない。とすると、本研究前半部の結論的推測には別の理由づけが必要となるように見える」と指摘されています。

 確かに我々は再提出稿81～82頁で無教会主義にふれ、異端から正統への変化を武田に依拠して触れていますが、ここのくだりでは、われわれは洗礼を必要としていないにも関わらず、なぜ日本で無教会主義の信者が増えないのかの理由を検討することをテーマには一切していません。日本で無教会主義の信者が増えないことに関しては様々な理由があると考えられますが、それを明らかにするためには別の考察が必要とされると思います。「「しかし、無教会グループは、今日、日本のキリスト教界の「正統」と一応考えられるキリスト教諸教派、諸グループの中に位置を占めつつある」という武田の文から、無教会派が、実際の少数派か否かは措いて、日本の一般人のイメージにおいて、キリスト教の一般的な姿と映る可能性の方にこそ、われわれの関心の中心はあります。続けて（再提出稿82頁の）次段落のように再臨運動に言及させて頂いております。

 「また内村は再臨運動をする中で、キリスト再臨の日には福音を信じない者にも『贖いの恩寵が光のごとく、万人にあまねくそそがれる』（若松 2018 p.180）と考えていた。あるいは堀孝彦は次のように内村の再臨運動を記す。『再臨を、ただ「教会問題」とみなしキリスト信者にのみかかわるものと見るのは大きな誤りだと言って、それが「宇宙問題、世界問題」であるから、万人に訴えて注意を喚起すべきだとしている。その根底には・・・万人救済論がある』（ 堀ほか 1996 pp.140-141 〔・・・は中略〕）。そのような内村の考え方がキリスト教の一般的姿として、日本のクリスチャンの内外から受け入れられ、それにあわせて 武田 (1973) のいうように、プロテスタントの聖職者・研究者界隈でも当初は異端でありつつ次第に受け入れられてきたとするならば、『信者にならなくても、キリスト教の教えを守ることによって、救われる可能性のある人を増やすこと』という回答は選ばれやすいといえる。」

 要するに、無教会主義をキリスト教会が次第に受け入れてきたこと自体が、寛容なキリスト教という像を一般の日本人に与えているということが、ここの箇所の趣旨です。洗礼というイニシエーションがないにもかかわらず、無教会主義への入信者が増えない理由に関しては、ここでは検討しません。実際にキリスト教会がどう考えるかは措いて、無教会主義を正統として受け入れた事実自体が、一般の人々にトリプルな信仰を否定はしない、キリスト教のイメージを形成させるというのが、ここの趣旨です。

 なお、このような誤解が生じていることの一つの理由としては、当該箇所で、「ミッション」の話から「他者救済の形の推測」（日本人がキリスト教の他者救済の形として何をイメージしているか）の話に移っていることが見えにくいということが考えられますので、ミッションの話と無教会主義の話のつなぎに以下の短い段落を挿入しました。

 「ここまで「トリプルな信仰」という見方にあらわれる、キリスト教の土着化の一つとして、「使命」としてのミッションの話に言及した。つぎに無教会主義が「トリプルな信仰」のいう見方を支える、日本人全般のキリスト教へのイメージ醸成に役立った点を論じたい。」

 なお、私事で恐縮ですが、数ある内村鑑三研究のうち、この研究を特に引用しているのは、研究代表者後藤の父親とこの本の編者の堀教授が名古屋学院大の同僚でかつ東大倫理学科の同期で、一緒に「内村鑑三研究会」を立ち上げたのに、暫くして父親が急逝いたため、この本の端書きで「畏友後藤宏行に捧ぐ」と書かれているので、これは何度も読んでいるということもあります。

 さて、頂いた査読ペーパーの最後は次のようになっています。

 「後半部の結論についてはおおむね分析の結果を信頼できるが、日本のキリスト教主義学校に多くの生徒・学生が集まることは、それらの学校の一般的なイメージが肯定的であることや学校が「布教」を主目的としていないことと「矛盾しない」、という判定だけではやや漠然としている。これも著者たちが自覚的に記していることであるが、キリスト教主義学校を志望する動機には、「進学・就職実績」など教育理念とは無関係の世俗的な利点が作用することが知られている。前半と後半が「矛盾しない」という判定を越えて、より積極的な因果関係ないし相関関係を見出すことに焦点が当てられるならば、本研究はいっそう大きな意義をもつことと思われる。」

 「それらの学校の一般的なイメージが肯定的であることや学校が「布教」を主目的としていないことと「矛盾しない」、という判定だけではやや漠然としている。」というご指摘の一つのお答えは、「学校が「布教」を主目的にしていないことに加えて、生徒に各人の「使命」にきづかさせる」点にあると考えています。ただ、今回はこれに関する記述は、現状の本稿ではカトリック女子中高校の公式ウェブの記述からの小結論に過ぎないことに加え、基本的に本稿はキリスト教徒やキリスト教学校自身がどう考えるかではなく、普通の若年層がキリスト教やキリスト主義学校にどういうイメージを抱くかに主眼があるので、このことを結論に大書することはできません。なお、本稿の筆頭著者もメンバーで本稿のメンバーの全員が共著者であるものの、本稿の私以外の共著者が筆頭著者を務める別の論文でこのことは扱っていますが、英語論文で査読結果待ちです。

 最後に、インタビュー調査についてですが、査読ペーパーの「評価」の前半箇所では、「数値結果に具体的な肉付けをするためのインタビューを行い、ウェブや文献の資料も参看するなど、調査には独自で入念な手順が踏まれている。」という形で、一応前向きに評価していただいたものの、同じ査読ペーパーの後半部分では「東京都と神奈川県の数字を「日本」の数字へと外挿することの是非などにも説明が望ましい。これらの空隙を補填するかのようにいくつかのインタビュー会話が記載されているが、いずれも個的で多義的なエピソードで、客観的なデータを補完するとまでは言えない」とやや後ろ向きの評価です。よって、インタビュー結果提示部分は、初回提出稿のうち、厳選されたもののみを残す方針にさせていただきました。

 具体的には「変更履歴稿」の方で二重取り消し線のある、インタビュー部分が削除された部分になります。これはこの先生への回答の２ページ目の1パラの「「変更履歴稿」の方で二重取り消し線のある部分が」という部分も同様です。

 また削除ではなく逆に加筆された部分は修正稿の赤線部分になりますが、先生のご著書をあらたに引用させていただく際等は、赤字にするのを忘れて、黒字のままのところもございますかも知れません。その点お許しください。

 以上よろしくお願い申し上げます。

 後藤

# Reviewer report: Reviewer response for version 1

**DOI:** 10.5256/f1000research.139996.r270385

**Published:** 2024-06-07

**Authors:** Shuhei Fujii

**Affiliations:** 1 Tokyo Kasei University, Tokyo, Japan

**論文の要約**

 　本論文は、日本のキリスト教徒は人口の約1.6％しか存在しないにもかかわらず、キリスト教主義学校が日本で人気を集めている理由を究明するために、東京都・神奈川県在住の 15～29 歳を対象としたウェブモニター調査を実施し、その結果の分析を行っている。

 　その際の作業仮説として、「（1）キリスト教主義学校は何を目的として設立されているか」「（2）キリスト教主義学校の生徒・学生イメージを、人びとがどのように捉えているか」を明らかにすることによって、日本のキリスト教徒は人口の1.6％しか存在しないにもかかわらず、キリスト教主義学校が日本で人気を集めている理由が解明されると想定されている。また、日本人は仏教と神道に加え、キリスト教も緩やかに信仰しているのではないかという「トリプルな信仰」説を踏まえ、（3）そのような信仰の有無を明らかにすることもまた、第3の作業仮説として設定されている。

 　質問票における質問項目は多岐にわたっているが、本論文では主に3つの作業仮説と関連する質問の結果が分析されている。作業仮説1については、キリスト教主義学校への関与度が低い回答者は布教や資金集めをキリスト教主義学校の目的と考えやすかったが、関与度が高い回答者はその割合が少なく、「信者にならなくても、キリスト教の教えを守ることによって、救われる可能性のある人を増やすこと」の回答が多かった。こうした点から、結果は日本にキリスト教徒が少ないにもかかわらずキリスト教主義学校が人気を集めている状況と矛盾しないと述べられている。

 　作業仮説2については、キリスト教主義学校に通う生徒が「みめうるわしい」というイメージはあまり抱かれておらず、一方で「外国語が得意である」「教養が豊かである」「品がよい」などの肯定的イメージが目立ち、これがキリスト教主義学校の人気の理由とみなしうるとしている。

 　作業仮説3については、キリスト教主義学校の通学経験者は仏教や神道の宗教的行事にもより参加している傾向があり、必ずしも信者にならないとしても、キリスト教の教えを守ったり、宗教的情操を涵養したりすることがキリスト教主義学校に期待されていることから、この仮説も裏付けられたとしている。

 　この結果を踏まえ、そのような複合的な信仰の形態がありうることを考慮すると、日本のキリスト教徒が1.6％だということは、見かけ上低い数値であるにすぎないという可能性を指摘している。

**本論文の評価**

 　本論文は、キリスト教主義学校の目的やその生徒について人々が抱いている意識を実証的に明らかにしている点だけでなく、「トリプルな信仰」という視点から、特定宗教を信仰する／信仰しないという従来の二者択一的な宗教観を見直し、明確に信仰せずとも、教えに共感をもつ、宗教的情操を涵養するといった形での宗教への関与のあり方を提示している点もまた有意義である。

 この視点は、現代の宗教の状況についての他の研究とも整合的である。米国では「Believing without Belonging」という言葉がしばしば用いられている。たとえばタナカ（2010）は米国には300万人の仏教徒がいるだけではなく、250万人ほどの仏教同調者もおり、さらに約2500万人が仏教から「重要な影響」を受けているとしている。

 　同時に、宗教組織への明確な所属を伴わない「スピリチュアル」な活動に関心を持つ人も増加しており、アメリカ心理学会の第36部門は2010年に「宗教とスピリチュアリティ学会」へと改名しているほか、日本でもパワースポット巡りや占い、ヒーリングなどが人気を集めている。

 　そうした状況において、日本のキリスト教についても同調者という形で信者数以上の関係者が存在しており、その同調者を増やすためにキリスト教主義学校が寄与しているという仮定は妥当なものだといえ、本研究結果でもその仮定が裏付けられている。

 　他方で、本論文にはいくらか不足していると思われる点が見受けられる。それらについて以下に指摘していきたい。

**先行研究の扱いについて**

 　全体として、本論文が参照している先行研究は不十分である。金児（2011）が指摘しているように、戦後の宗教心理学的研究においてはキリスト教系学校を対象とした質問紙調査が多数行われており、決して不足しているわけではない。

 　例として、寺川（1983）はキリスト教を含む宗教系学校に質問紙を送り、教育理念や実践について尋ねている。真野（1984）はプロテスタントのクリスチャン・スクールに宗教教育の目的・現状・成果などを尋ねている。生徒を対象としたものとしては、真野（1986）がキリスト教主義高等学校の女子生徒に対し、本論文でも扱っているような自校のイメージについて尋ねた例がある。

 　より新しい研究としては、現在進行中のものであるが、清泉女子大学の森本真由美氏が上智大学教育イノベーションプロジェクトとして、カトリック大学の伝統をどのように学生に継承していくかという課題から、面接調査を実施している。その成果の一部は、日本心理学会第87回大会の公募シンポジウム「神学・宗学は実証的宗教心理学と連携・協働することができるのか――実証的宗教心理学の挑戦（2）」として発表されている。

 　確かに、一般の回答者に対してキリスト教主義学校のイメージを尋ねた研究は管見の限りでは見当たらないが、キリスト教系高校出身者と非キリスト教系高校出身者のキリスト教への態度の差などを調査した中村（1976）などもあり、本論文の目的からして十分参照に値するものである。

 　また、「2. ウェブモニター調査の概要」ではキリスト教徒の割合の比較先として、井上らの2000年の文献が参照されているが、より新しい文献を用いるべきだろう。ISSPの結果を用いている小林（2019）ではキリスト教を信仰していると答えた割合は1.2％、國學院大學研究開発推進機構日本文化研究所（2022）の結果はキリスト教系大学生を多く含むものにもかかわらず、約1.4％となっている。総じて近年になるほど数値は減少する傾向にあるので、これらと比較すれば本論文の3.0％という数値はさらに際立つといえる。

 　さらに、前述の小林（2019）に加え庭野平和財団（2020）はキリスト教を含む宗教への肯定感やイメージについて尋ねており、こちらの結果も本論文にとって有意義なものとなるだろう。

**結果の取り扱いについて**

 　それぞれの結果の分析は概ね妥当と思われるが、事前の仮説に反する結果の取り扱いにやや不十分な点が感じられる。たとえば、Q34のキリスト教主義学校に対する設置母体のメリットとして、「教団組織の資金集め」が最も多く選ばれている。これは現実とは乖離しているということであるが、それにもかかわらずなぜそのようなイメージが持たれているかは検討の余地があるのではないだろうか。そしてこの回答は、決してキリスト教主義学校に対してよいイメージをもっていることを示すものではないだろう。このようなネガティブな選択肢がもっと含まれていた場合、いかなる結果になったのかは気になる点である。

 　同様に、Q29のキリスト教主義学校の学生のイメージとして、「外国語が得意である」が最多となった点もより重視するべきであるように思われる。井上ほか（2018）においても、「女子アナが多い」など、実利的な面が大いに強調されているし、実際に有名なキリスト教系大学は、英語教育などに力を入れていることは確かであろう。そのため人格面に加え、「就職に有利である」「高収入である」などの実利的な質問を増やし、人格面とどちらが重視されるかを検討することも可能と思われる。

 　こうした点は問題点というよりは、今後の課題とみなしうるものだろう。一足飛びに仮説が検証されたとみなすのではなく、予期していなかった結果に目を向けることで、ますます多くの事実を明らかにすることができるように思われる。

**改善の提案**

 　本論文の改善点として提案できることは、第一に先行研究の参照を増やすことである。先行するキリスト教主義学校の調査や世論調査を十分に参照することによって、本論文がいかに新しい知見をもたらしたかという点をより明確にすることができるだろう。

 　第二の点は、議論を整理することである。本論文は分量が非常に多く、途中にインタビューの記述なども差し挟まれ、把握しづらいものとなっている。「3. 調査結果とその分析」においても、それぞれの節が3つの作業仮説といかに対応しているかがわかりづらい。それぞれの節の冒頭にナビゲーションとなる解説を付加し、個々の質問項目の検討はいかなる疑問を明らかにするために行っているのかを明確にし、取り上げる質問項目を取捨選択することによって、この点は改善されるであろう。

 　また「5. 考察」においても、キリスト教の布教のあり方やキリスト教主義学校の目的はどのようであるべきかといった点に議論が迂回しているように思われる。もしこの主題を論じることが元来の目的であるならば、その点を「1. 先行研究と問題の所在」に明記すべきである。

**参考文献**

 井上 章一ほか（2018）．ミッションスクールになぜ美人が多いのか――日本女子とキリスト教　朝日新聞出版

 金児 暁嗣（監修）松島 公望・河野 由美・杉山 幸子・西脇 良（編）（2011）．宗教心理学概論　ナカニシヤ出版

 小林 利行（2019）．日本人の宗教的意識や行動はどう変わったか　放送研究と調査， 69(4), 52-72.

 國學院大學研究開発推進機構日本文化研究所（編）（2022）．第13回学生宗教意識調査報告―改訂増補版―　國學院大學研究開発推進機構日本文化研究所

 真野 一隆（1984）．私立学校の中・高一貫教育の原理と内容に関する理論的並びに実践的研究――プロテスタントのクリスチャン・スクールに焦点をあてて　日本私学教育研究所紀要， 20(1), 89-134.

 真野 一隆（1986）．私立学校の女子教育の歴史と現実――プロテスタントのクリスチャン・スクールに焦点をあてて　日本私学教育研究所紀要， 22(1), 37-72.

 中村 陽三（1976）．青年期における宗教意義に関する実証的比較研究――公立校生徒とキリスト教学校生徒との比較 日本私学教育研究所紀要， 11(1), 157-173.

 庭野平和財団（2020）．日本人の宗教団体への関与・認知・評価に関する世論調査報告書　庭野平和財団　https://www.npf.or.jp/pdf/2020_research.pdf

 タナカ ケネス（2010）．アメリカ仏教――仏教も変わる、アメリカも変わる　武蔵野大学出版会

 寺川 幽芳（1983）．私立大学における宗教教育の現状をめぐって 仏教文化研究所研究紀要， 13, 13-55.

（該当する場合は要回答）統計分析および解釈は適切ですか。

はい

本研究は明確かつ正確に提示されたものであり、最新の文献を引用していますか。

一部該当

方法と分析について第三者による再現が可能となるよう十分な詳細が提示されていますか。

はい

結果の基礎となるソースデータはすべて入手可能で再現性を十全に保証していますか。

はい

結論は結果により妥当な裏付けを得ていますか。

一部該当

研究設計は適切で学術的価値がありますか。

はい

Reviewer Expertise:

宗教学　宗教心理学　宗教認知科学

I confirm that I have read this submission and believe that I have an appropriate level of expertise to confirm that it is of an acceptable scientific standard.
